# The rs17782313 polymorphism near MC4R gene confers a high risk of obesity and hyperglycemia, while PGC1α rs8192678 polymorphism is weakly correlated with glucometabolic disorder: a systematic review and meta-analysis

**DOI:** 10.3389/fendo.2023.1210455

**Published:** 2023-08-09

**Authors:** Youjin Zhang, Shiyun Li, Haiyan Nie, Xue Wang, Xuanxuan Li, Jinhui Wen, Mengxi Li, Yongyan Song

**Affiliations:** ^1^Central Laboratory, Clinical Medical College and Affiliated Hospital of Chengdu University, Chengdu, Sichuan, China; ^2^Department of Endocrinology, Clinical Medical College and Affiliated Hospital of Chengdu University, Chengdu, Sichuan, China; ^3^Clinical Medical College of Chengdu University, Chengdu, Sichuan, China

**Keywords:** MC4R, PGC1α, rs17782313, rs8192678, obesity, hyperglycemia

## Abstract

**Background:**

The relationships of the rs17782313 polymorphism near melanocortin 4 receptor gene (MC4R) and the rs8192678 polymorphism in peroxisome proliferator-activated receptor gamma coactivator 1 alpha gene (PGC1α) with metabolic abnormalities have been explored in many populations around the world, but the findings were not all consistent and sometimes even a bit contradictory.

**Methods:**

Electronic databases including Medline, Scopus, Embase, Web of Science, CNKI and Google Scholar were checked for studies that met the inclusion criteria. Data were carefully extracted from eligible studies. Standardized mean differences (SMDs) were calculated by using a random-effects model to examine the differences in the indexes of obesity, glucometabolic disorder and dyslipidemia between the genotypes of the rs17782313 and rs8192678 polymorphisms. Cochran’s Q-statistic test and Begg’s test were employed to identify heterogeneity among studies and publication bias, respectively.

**Results:**

Fifty studies (58,716 subjects) and 51 studies (18,660 subjects) were respectively included in the pooled meta-analyses for the rs17782313 and rs8192678 polymorphisms. The C-allele carriers of the rs17782313 polymorphism had a higher average level of body mass index (SMD = 0.21 kg/m^2^, 95% confidence interval [95% CI] = 0.12 to 0.29 kg/m^2^, *p* < 0.001), waist circumference (SMD = 0.14 cm, 95% CI = 0.06 to 0.23 cm, *p* < 0.001) and blood glucose (SMD = 0.09 mg/dL, 95% CI = 0.02 to 0.16 mg/dL, *p* = 0.01) than the TT homozygotes. Regarding the rs8192678 polymorphism, no significant associations with the indexes of obesity, glucometabolic disorder and dyslipidemia were detected. However, significant correlations between the rs8192678 polymorphism and multiple glucometabolic indexes were observed in subgroup analyses stratified by sex, age, ethnicity and health status.

**Conclusion:**

The meta-analysis demonstrates that the C allele of the MC4R rs17782313 polymorphism confers a higher risk of obesity and hyperglycemia, and the PGC1α rs8192678 polymorphism is weakly correlated with glucometabolic disorder. These findings may partly explain the relationships between these variants and diabetes as well as cardiovascular disease.

**Systematic review registration:**

https://www.crd.york.ac.uk/prospero/, identifier CRD42022373543.

## Introduction

Cardiovascular disease (CVD) remains the number one killer among the world’s population, and the mortality and severe disability rates are still on the increase in some countries such as China (1–3). Metabolic disorders have been recognized as the most important risk factors for CVD, accounting for approximately 50% of the population-attributable risk (4). Genetic variations play important roles in the occurrence and progression of both metabolic disorders and CVD (5–7). In recent years, more and more susceptibility genes as well as genetic polymorphisms to metabolic disorders and CVD have been explored and confirmed *in vitro* and *in vivo* studies. The genes and their polymorphisms from the melanocortin system and peroxisome proliferator activated receptor signaling pathway have been extensively investigated with regard to their relations with obesity, metabolic abnormalities and CVD (8–12).

Melanocortin 4 receptor (MC4R), a member of the G protein-coupled receptor family and with alpha-melanocyte-stimulating hormone (a-MSH) as its ligand, is mainly distributed in the ventromedial hypothalamus and is a crucial modulator of food intake and energy homeostasis (13). Human MC4R gene is situated at the 18q21 region of chromosome 18. The rs17782313 polymorphism, located approximately 190 kilobases downstream of the MC4R gene, is a single nucleotide variation changed from thymine (T) to cytosine (C). The C allele of the rs17782313 variant was widely reported to be linked to metabolic disorders such as obesity, hyperglycemia and dyslipidemia in children (14, 15) and adults (16–24). Peroxisome proliferator-activated receptor gamma coactivator 1 alpha (PGC1α) is a coactivator of peroxisome proliferator-activated receptor-γ (PPARγ) and possibly of several other transcriptional factors such as estrogen-related receptor, nuclear respiratory factor 1 and hypoxia inducible factor 1 alpha (25–28). PGC1α plays a crucial role in mitochondrial biogenesis, fatty acid oxidation and thermogenesis (29–31). A missense variant rs8192678 (also known as +1564G/A or p.Gly482Ser), located in exon 8 of PGC1α gene, is formed by a single-nucleotide variation from guanine (G) to adenine (A), leading to the amino acid substitution from glycine to serine. A series of studies have demonstrated that the A allele of the rs8192678 polymorphism is associated with an elevated risk of adiposity (32–34) and type 2 diabetes mellitus (T2DM) (35–37).

However, there are many inconsistencies and contradictions in the published data on these two polymorphic sites and metabolic abnormalities. Some researchers reported that C allele of the rs17782313 polymorphism is correlated with a higher level of body mass index (BMI) (38–43), waist circumference (WC) (40, 44), waist-to-hip ratio (WHR) (23), glucose (GLU) (16–18), insulin (INS) (14), triglyceride (TG) (19–21), total cholesterol (TC) (21) or low-density lipoprotein cholesterol (LDL-C) (21), and a lower level of high-density lipoprotein cholesterol (HDL-C) (45), while the experimental data obtained by other investigators could not support these conclusions (46–53). There were also lots of disagreements on the relations between the PGC1α rs8192678 variant and the indexes of obesity, glucometabolic disorder and dyslipidemia. Some researchers claimed that the individuals carrying the A allele of the rs8192678 variant had a higher level of BMI (54), WHR (33, 54), GLU (55–57), INS (58–61) and homeostasis model assessment of insulin resistance (HOMA-IR) (58–61), TG (62–66), TC (67) or LDL-C (66, 67), and a lower level of HDL-C (55, 65, 66) than the noncarriers, but other scientists failed to replicate these findings in their well-designed studies and thereby had to report no associations of the rs8192678 variant with the metabolic indexes (68–74).

The systematic review and meta-analysis was conducted to clarify the relationships between the rs17782313 and rs8192678 variants and the indexes of obesity, glucometabolic disorder and dyslipidemia. The obtained findings can help clarify the interrelationships among the rs17782313 and rs8192678 variants, metabolic abnormalities and CVD.

## Methods

### Literature search for eligible studies

This meta-analysis has been registered and supported by PROSPERO International Prospective Register of Systematic Reviews (identifier CRD42022373543), following the Preferred Reporting Items for Systematic Reviews and Meta-analysis (PRISMA) statement. Electronic databases such as Medline, Scopus, Embase, Web of Science, CNKI and Google Scholar were thoroughly retrieved from 1980 to February 2023. The literature search keywords were as follows: (“melanocortin 4 receptor” or “MC4R”) and (“peroxisome proliferator-activated receptor gamma coactivator 1 alpha” or “PGC1α” or “PPARGC1A”) and (“rs17782313” or “rs8192678” or “variant” or “polymorphism” or “variance” or “mutation”) and (“body mass index” or “BMI” or “waist circumference” or “WC” or “waist-to-hip ratio” or “WHR”) and (“glucose” or “GLU” or “insulin” or “INS” or “homeostasis model assessment of insulin resistance” or “HOMA-IR”) and (“lipid” or “triglyceride” or “TG” or “total cholesterol” or “TC” or “low-density lipoprotein cholesterol” or “LDL-C” or “high-density lipoprotein cholesterol” or “HDL-C”). The parameters assessed in the meta-analysis include three obesity indexes (WHR, WC and BMI), three glucometabolic disorder indexes (HOMA-IR, INS and GLU), and four dyslipidemia indexes (HDL-C, LDL-C, TC and TG). Any studies that explored the relations of the rs17782313 and rs8192678 variants with the ten indexes were screened and reviewed.

### Inclusion and exclusion criteria

Inclusion criteria: 1) The genotype distribution and sample size were presented clearly and accurately; 2) One or more of the ten variables (BMI, WC, WHR, GLU, INS, HOMA-IR, TG, TC, LDL-C, and HDL-C) were reported; 3) Data format of the variables were shown as mean/standard deviation or mean/standard error. Exclusion criteria: 1) Repeatedly published data of the variables; 2) Low quality data; 3) Case reports or conference abstracts.

### Data extraction

Data were carefully checked and verified after extraction from the enrolled articles. The data extracted from each article were as follows: title, year of publication, author’s names, age, gender, ethnicity, health status, sample size, and means as well as standard deviations/standard errors by genotypes. Standard deviation was calculated if standard error was given. Units used for the variables included kg/m^2^ (BMI), cm (WC), μmol/μL (INS) and mg/dL (TG, TC, LDL-C, HDL-C and GLU), and units were converted if other units were used.

### Meta-analysis

The STATA software package (College Station, TX, USA) was employed in this systematic review and meta-analysis. Most of the included studies presented their research results in a dominant way, therefore the present meta-analysis also adopted the dominant model (i.e., [CC + CT] vs. TT for the rs17782313 polymorphism and [AA + AG] vs. GG for the rs8192678 polymorphism). Subgroup analyses were conducted according to gender, age, ethnicity and health condition. Each subgroup was defined as a comparison in the meta-analysis, and subgroup analyses were performed with three or more comparisons to ensure statistical power. Standardized mean difference (SMD) was employed to express the differences in the indexes of obesity, glucometabolic disorder and dyslipidemia between the genotypes of the rs17782313 and rs8192678 polymorphisms. The random effects model usually provides a more reliable analysis result, so this study selected the random effects model for analysis. Cochran’s Q-statistic test was used to assess heterogeneity among the studies, and Galbraith plot was drawn to discover the potential sources of heterogeneity. Subgroup analyses were performed, and the subgroups were classified by gender (males and females), age (adults and children/adolescents), ethnicity (European Caucasians, South Americans, East Asians and West Asians), and health condition (overweight/obesity, T2DM, polycystic ovary syndrome, hypertension and general/control subjects). Begg’s test was conducted to evaluate publication bias, and the trim and fill method was used to assess the potential influence of the missing studies on the pooled result if there was a publication bias. All analyses were two-tailed, and *p* ≤ 0.05 was considered as statistically significant.

## Results

### Characteristics of the included studies

**Figure 1:**
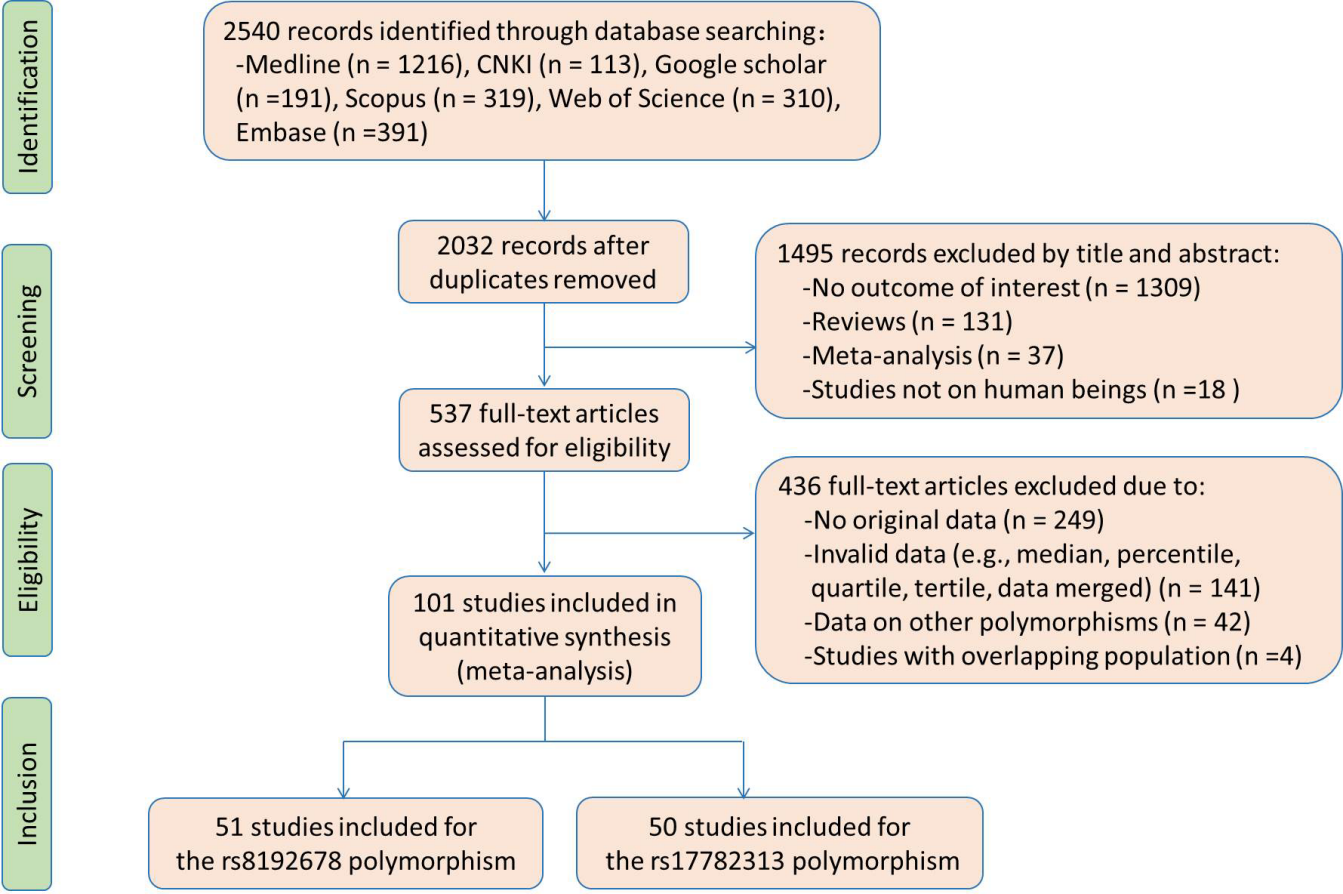
The flow diagram of the literature search.

Flow diagram of the literature search is displayed in **Figure 1**. Fifty studies and 51 studies were respectively enrolled for the rs17782313 and rs8192678 polymorphisms, and the reference list of the included studies is presented in **Table S1**. Characteristics of the studies included for the rs17782313 variant are shown in **Table S2**. The included studies were published from 2009 to 2022, and written either in Chinese (2 articles) or English language (48 articles). Twenty-three studies, 12 studies, 7 studies and 2 studies involved European Caucasians, East Asians, West Asians and South Asians, respectively. Twenty-seven studies, 12 studies, 5 studies, and 3 studies involved overweight/obesity, T2DM, hypertension and hyperlipidemia, respectively. One study only involved males, 9 studies only involved females, and the rest studies involved both genders. The study populations in 14 studies were divided into subgroups based on gender and health condition, and each subgroup served as an independent comparison in the analysis. Original data of the indexes by genotypes of the rs17782313 polymorphism are shown in **Tables S3**-**S5**. Forty-five studies, 24 studies, 12 studies, 26 studies, 15 studies, 14 studies, 22 studies, 22 studies, 21 studies and 22 studies presented the data for BMI, WC, WHR, GLU, INS, HOMA-IR, TG, TC, LDL-C and HDL-C, respectively. A total of 65 comparisons were distinguished among the 50 studies included for the rs17782313 polymorphism, and 56 comparisons, 30 comparisons, 15 comparisons, 30 comparisons, 18 comparisons, 16 comparisons, 27 comparisons, 27 comparisons, 27 comparisons and 28 comparisons were included to analyze the differences in BMI, WC, WHR, GLU, INS, HOMA-IR, TG, TC, LDL-C and HDL-C, respectively. Fifty-eight thousand seven hundred and sixteen subjects were enrolled in the meta-analysis for the rs17782313 polymorphism. Fifty-nine percent of these subjects had the TT genotype, and the rest had the CT or CC genotype.

Characteristics of the studies included for the rs8192678 variant are shown in **Table S6**. The included studies were published from 2001 to 2022, and written either in Chinese (13 articles) or English language (38 articles). Thirty studies, 12 studies, 3 studies and 4 studies involved European Caucasians, East Asians, South Americans and West Asians, respectively. Eleven studies, 13 studies, 4 studies, 2 studies, 2 studies and 2 studies involved overweight/obesity, T2DM, polycystic ovary syndrome, nonalcoholic fatty liver disease, hypertension and metabolic syndrome, respectively. Four studies only involved males, 6 studies only involved females, and the other studies involved both genders. There were 17 studies in which the study populations were divided into subgroups based on gender and health condition, and each subgroup was served as an independent comparison in the analysis. Original data of the indexes by genotypes of the rs8192678 polymorphism are shown in **Tables S7**-**S9**. Forty-seven studies, 14 studies, 12 studies, 33 studies, 22 studies, 18 studies, 36 studies, 34 studies, 28 studies and 34 studies provided the data for BMI, WC, WHR, GLU, INS, HOMA-IR, TG, TC, LDL-C and HDL-C, respectively. Seventy-one comparisons were distinguished among the 51 studies included for the rs8192678 polymorphism, and 64 comparisons, 16 comparisons, 21 comparisons, 43 comparisons, 30 comparisons, 25 comparisons, 47 comparisons, 45 comparisons, 36 comparisons and 45 comparisons were respectively included to analyze the differences in BMI, WC, WHR, GLU, INS, HOMA-IR, TG, TC, LDL-C and HDL-C. Eighteen thousand six hundred and sixty subjects were enrolled in the meta-analysis for the rs8192678 variant. Thirty-nine percent of these subjects had the GG genotype, and the rest subjects had the AA or AG genotype.

### Associations of the MC4R rs17782313 polymorphism with the indexes of obesity, glucometabolic disorder and dyslipidemia

**Figure 2:**
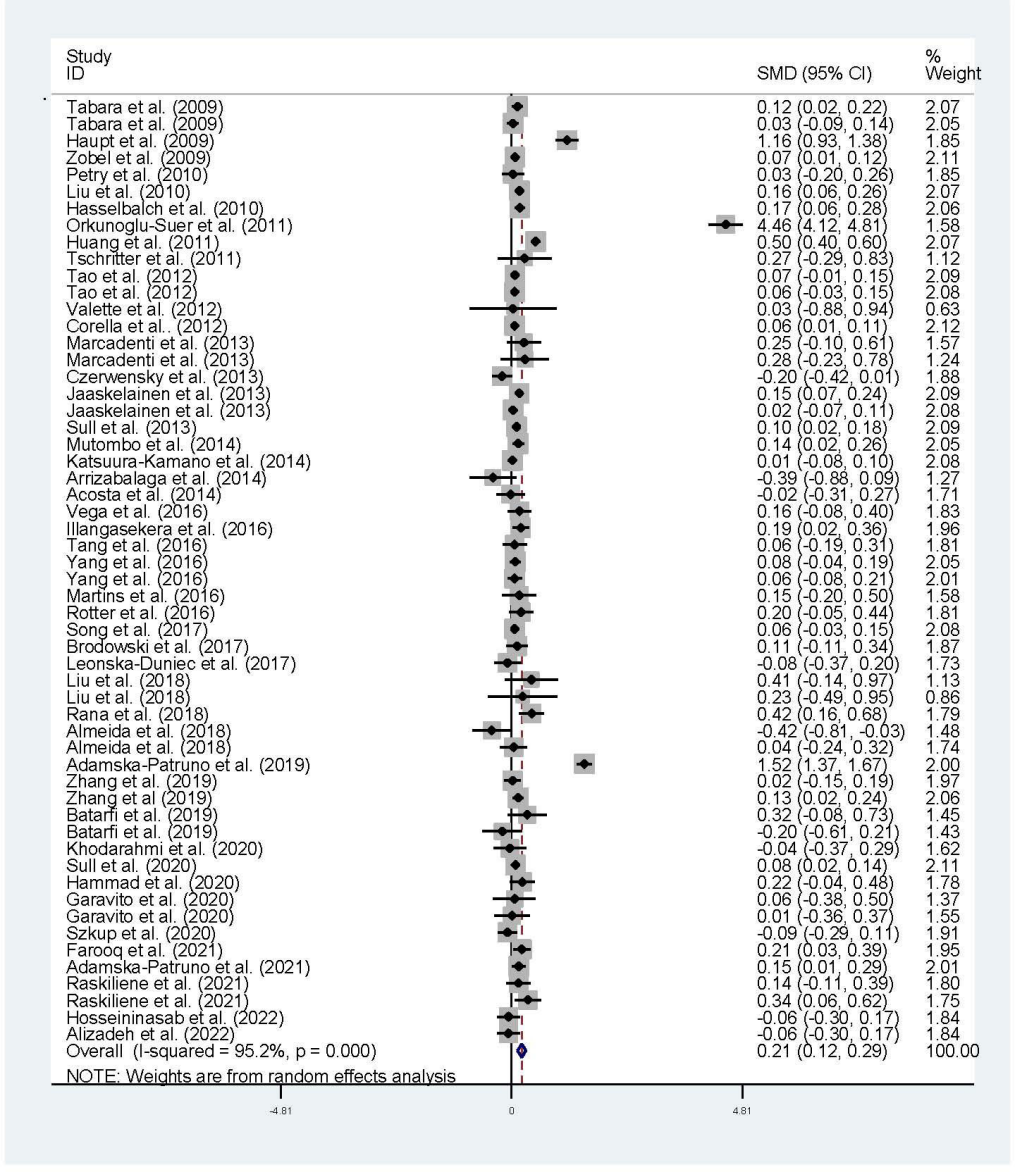
Forest plot of the association analysis between the MC4R rs17782313 polymorphism and BMI. MC4R, melanocortin 4 receptor, SMD, standardized mean difference: 95% CI, 95% confidence interval; BMI, body mass index.

**Figure 3:**
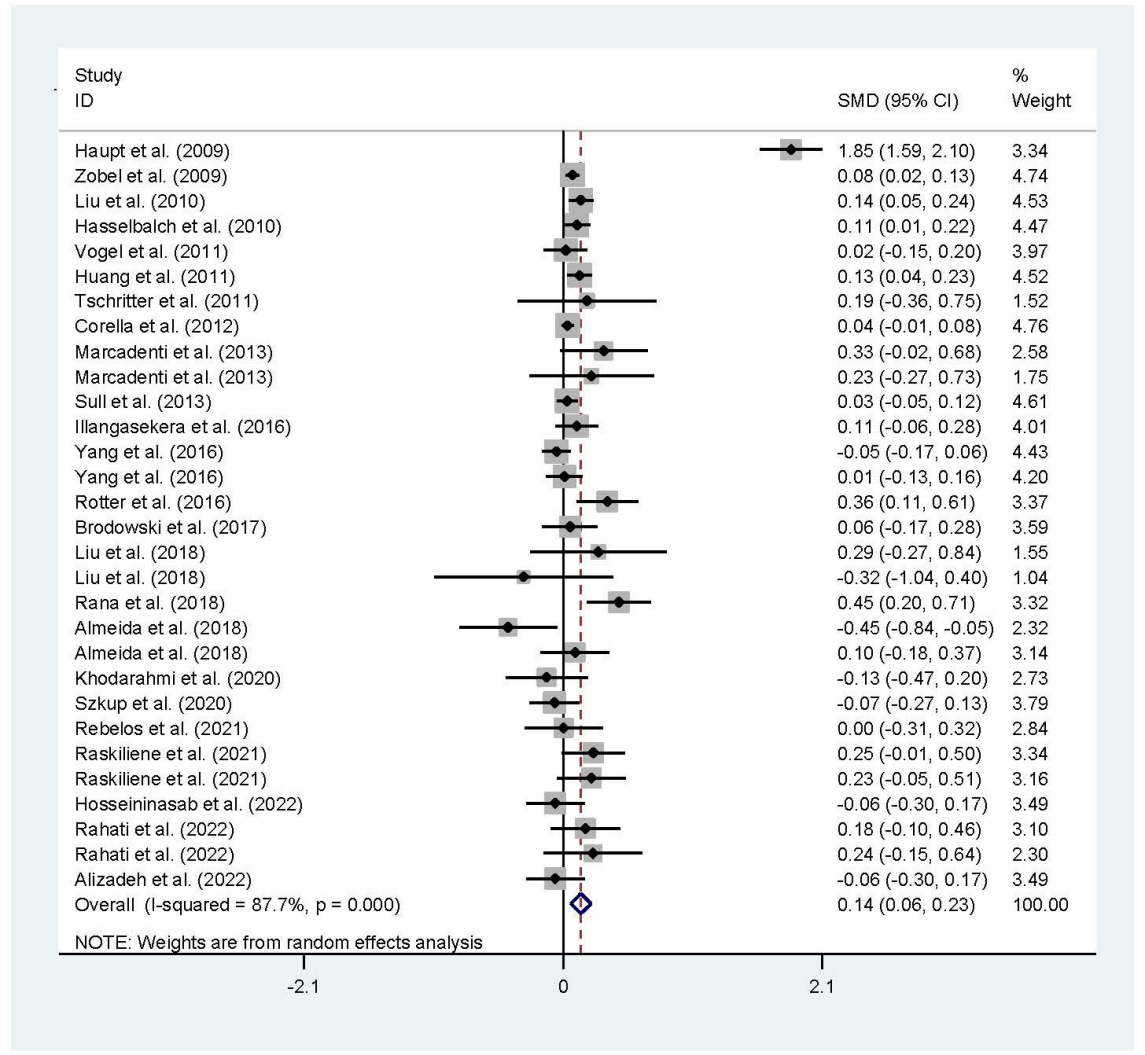
Forest plot of the association analysis between the rs177782313 polymorphism and WC. MC4R, melanocortin 4 receptor; SMD, standardized mean difference: 95%, CI, 95% confidence interval; WC, waist circumtances.

**Figure 4:**
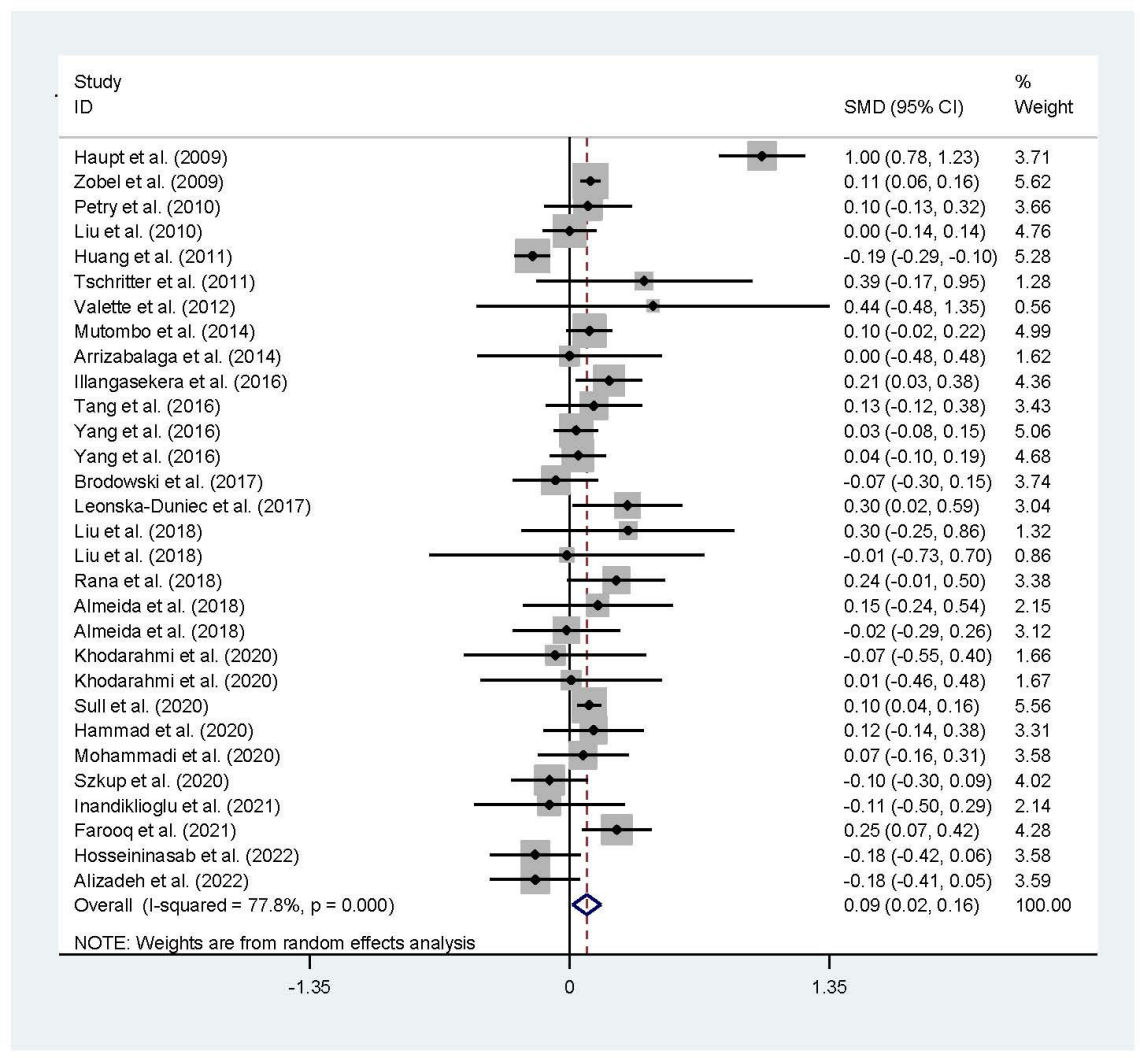
Forest plot of the association analysis between the MC4R rs17782313 polymorphism and blood glucose. MC4R, melanocortin 4 receptor; SMD, standardized mean difference; 95% CI, 95% confidence interval.

The associations of the rs17782313 polymorphism with the indexes of obesity, glucometabolic disorder and dyslipidemia are shown in **Tables 1**–**3**; **Figures 2**–**4**, **S1**–**S7**. The pooled meta-analyses displayed that individuals carrying the C allele of the rs17782313 variant had a higher level of BMI (SMD = 0.21 kg/m^2^, 95% confidence interval [95% CI] = 0.12 to 0.29 kg/m^2^, *p* < 0.001) (**Table 1**; **Figure 2**), WC (SMD = 0.14 cm, 95% CI = 0.06 to 0.23 cm, *p* < 0.001) (**Table 1**, **Figure 3**) or blood glucose (SMD = 0.09 mg/dL, 95% CI = 0.02 to 0.16 mg/dL, *p* = 0.01) (**Table 2**; **Figure 4**) than the TT homozygotes in the overall population. No significant associations of the rs17782313 variant with the levels of WHR, INS, HOMA-IR, TG, TC, LDL-C and HDL-C were detected in the overall population (**Tables 1****–** 3; **Figures S1**–**S7**).

**Table 1:** Meta-analyses between the MC4R rs17782313 polymorphism and obesity indexes.

Groups or subgroups	Comparisons (Subjects)	SMD (95% CI)	*P*_Heterogeneity_	*P*_SMD_
BMI (CC + CT vs. TT)				
All subjects	56 (55015)	0.21 (0.12, 0.29)	< 0.001	< 0.001
Males	7 (4792)	0.07 (0.001, 0.14)	0.33	0.048
Females	16 (8157)	0.33 (0.01, 0.65)	< 0.001	0.046
Adults	48 (45748)	0.24 (0.14, 0.34)	< 0.001	< 0.001
Children/adolescents	8 (9267)	0.08 (0.01, 0.14)	0.06	0.03
European Caucasian	23 (26277)	0.18 (0.05, 0.31)	< 0.001	0.01
South American	8 (1573)	0.67 (-0.39, 1.73)	< 0.001	0.22
East Asian	17 (25299)	0.11 (0.05, 0.17)	< 0.001	< 0.001
West Asian	6 (1174)	0.02 (-0.12, 0.16)	0.25	0.76
Overweight/obesity patients	16 (4353)	0.08 (-0.08, 0.25)	< 0.001	0.32
T2DM patients	5 (9340)	0.08 (0.01, 0.16)	0.19	0.03
Hypertensive patients	6 (9132)	0.08 (0.04, 0.13)	0.41	< 0.001
General/control subjects	19 (25287)	0.33 (0.17, 0.50)	< 0.001	< 0.001
WC (CC + CT vs. TT)				
All subjects	30 (27259)	0.14 (0.06, 0.23)	< 0.001	< 0.001
Males	5 (1355)	0.15 (-0.04, 0.34)	0.10	0.11
Females	8 (3068)	0.02 (-0.07, 0.12)	0.19	0.63
Adults	26 (24785)	0.17 (0.08, 0.26)	< 0.001	< 0.001
Children/adolescents	4 (2474)	0.02 (-0.16, 0.20)	0.03	0.83
European Caucasian	16 (19522)	0.19 (0.06, 0.32)	< 0.001	0.004
West Asian	5 (1106)	0.01 (-0.12, 0.13)	0.38	0.93
East Asian	6 (6144)	0.04 (-0.04, 0.11)	0.16	0.31
Overweight/obesity patients	14 (4586)	0.07 (-0.04, 0.18)	0.003	0.20
T2DM patients	3 (7916)	0.09 (-0.10, 0.27)	0.02	0.36
Hypertensive patients	4 (7708)	0.21 (-0.01, 0.42)	0.03	0.06
General/control subjects	9 (14047)	0.24 (0.05, 0.42)	< 0.001	0.01
WHR (CC + CT vs. TT)				
All subjects	15 (10291)	0.05 (-0.11, 0.22)	< 0.001	0.53
Females	4 (1038)	-0.05 (-0.67, 0.57)	< 0.001	0.88
Adults	13 (9965)	0.11 (-0.08, 0.29)	< 0.001	0.25
European Caucasian	8 (8975)	-0.13 (-0.39, 0.13)	< 0.001	0.32
West Asian	4 (965)	0.17 (0.03, 0.30)	0.41	0.02
Overweight/obesity patients	9 (1855)	0.10 (-0.25, 0.45)	< 0.001	0.59
General/control subjects	3 (6828)	0.03 (-0.18, 0.23)	0.002	0.80

MC4R, melanocortin 4 receptor; SMD, standardized mean difference; 95% CI, 95% confidence interval; BMI, body mass index; WC, waist circumference; WHR, waist-to-hip ratio; T2DM, type 2 diabetes mellitus.

**Table 2:** Meta-analyses between the MC4R rs17782313 polymorphism and glucometabolic disorder indexes.

Groups or subgroups	Comparisons (Subjects)	SMD (95% CI)	*P*_Heterogeneity_	*P*_SMD_
GLU (CC + CT vs. TT)				
All subjects	30 (20990)	0.09 (0.02, 0.16)	< 0.001	0.01
Females	9 (3008)	-0.03 (-0.13, 0.07)	0.17	0.59
Males	3 (836)	0.04 (-0.10, 0.18)	0.98	0.58
Adults	24(19168)	0.10 (0.02, 0.19)	< 0.001	0.02
Children/adolescents	6 (1822)	0.04 (-0.06, 0.13)	0.85	0.47
European Caucasian	14 (9335)	0.15 (0.01, 0.29)	< 0.001	0.03
East Asian	8 (9570)	0.04 (-0.07, 0.14)	< 0.001	0.51
West Asian	6 (1273)	-0.05 (-0.16, 0.07)	0.40	0.42
Overweight/obesity patients	15 (2412)	0.01 (-0.08, 0.09)	0.39	0.91
T2DM patients	4 (3640)	-0.03 (-0.21, 0.15)	0.001	0.76
General/control subjects	10 (12508)	0.15 (0.03, 0.27)	< 0.001	0.01
INS (CC + CT vs. TT)				
All subjects	18 (12277)	-0.05 (-0.34, 0.24)	< 0.001	0.73
Females	4 (856)	0.05 (-0.15, 0.24)	0.19	0.64
Adults	12 (9960)	0.05 (-0.38, 0.48)	< 0.001	0.81
Children/adolescents	6 (2317)	0.004 (-0.08, 0.09)	0.51	0.93
European Caucasian	10 (8969)	-0.06 (-0.19, 0.07)	< 0.001	0.39
West Asian	4 (711)	0.07 (-0.11, 0.25)	0.26	0.47
Overweight/obesity patients	10 (2334)	0.01 (-0.09, 0.11)	0.24	0.85
General/control subjects	6 (7610)	-0.09 (-0.30, 0.11)	< 0.001	0.38
HOMA-IR (CC + CT vs. TT)				
All subjects	16 (12541)	0.02 (-0.02, 0.07)	0.28	0.29
Females	5 (1199)	-0.07 (-0.18, 0.05)	0.76	0.27
Adults	11 (10566)	0.05 (-0.004, 0.10)	0.30	0.07
Children/adolescents	5 (1975)	-0.05 (-0.14, 0.04)	0.68	0.25
European Caucasian	9 (8629)	-0.01 (-0.05, 0.04)	0.64	0.79
West Asian	4 (709)	0.09 (-0.06, 0.25)	0.37	0.25
Overweight/obesity patients	10 (2468)	0.02 (-0.06, 0.10)	0.75	0.66
T2DM patients	3 (3358)	0.08 (-0.004, 0.17)	0.23	0.06
General/control subjects	4 (7140)	-0.04 (-0.13, 0.05)	0.19	0.36

MC4R, melanocortin 4 receptor; SMD, standardized mean difference; 95% CI, 95% confidence interval; GLU, glucose; INS, insulin; HOMA-IR, homeostasis model assessment of insulin resistance; T2DM, type 2 diabetes mellitus.

**Table 3:** Meta-analyses between the MC4R rs17782313 polymorphism and dyslipidemia indexes.

Groups or subgroups	Comparisons (Subjects)	SMD (95% CI)	*P*_Heterogeneity_	*P*_SMD_
TG (CC + CT vs. TT)				
All subjects	27 (17652)	-0.003 (-0.06, 0.06)	< 0.001	0.92
Females	9 (3008)	-0.11 (-0.31, 0.08)	< 0.001	0.26
Males	4 (1108)	-0.002 (-0.16, 0.16)	0.29	0.98
Adults	22 (16253)	-0.001 (-0.07, 0.06)	< 0.001	0.98
Children/adolescents	5 (1399)	-0.03 (-0.23, 0.18)	0.02	0.79
European Caucasian	11 (8769)	-0.02 (-0.15, 0.11)	< 0.001	0.80
East Asian	8 (7346)	< 0.001 (-0.07, 0.07)	0.10	0.99
West Asian	6 (1273)	-0.05 (-0.21, 0.11)	0.13	0.54
Overweight/obesity patients	15 (3139)	-0.06 (-0.21, 0.08)	< 0.001	0.39
T2DM patients	5 (3912)	0.06 (-0.06, 0.18)	0.04	0.35
Hypertensive patients	3 (1696)	0.06 (-0.06, 0.18)	0.29	0.34
General/control subjects	8 (10767)	0.05 (-0.02, 0.11)	0.07	0.18
TC (CC + CT vs. TT)				
All subjects	27 (20988)	0.00 (-0.05, 0.05)	< 0.001	0.99
Females	8 (2957)	0.05 (-0.08, 0.17)	0.04	0.47
Males	3 (1078)	0.02 (-0.10, 0.14)	0.93	0.73
Adults	22 (19588)	-0.01 (-0.07, 0.04)	< 0.001	0.66
Children/adolescents	5 (1400)	0.09 (-0.02, 0.20)	0.59	0.09
European Caucasian	12 (8791)	0.02 (-0.03, 0.07)	0.40	0.49
East Asian	7 (10660)	-0.02 (-0.11, 0.08)	< 0.001	0.72
West Asian	6 (1273)	0.03 (-0.13, 0.19)	0.12	0.72
Overweight/obesity patients	14 (3080)	0.05 (-0.03, 0.13)	0.37	0.19
T2DM patients	4 (2770)	0.01 (-0.17, 0.19)	0.01	0.93
General/control subjects	8 (10767)	0.03 (-0.05, 0.11)	0.01	0.44
LDL-C (CC + CT vs. TT)				
All subjects	27 (14942)	0.01 (-0.05, 0.07)	< 0.001	0.79
Females	8 (2931)	0.06 (-0.08, 0.21)	0.01	0.40
Males	4 (1108)	0.08 (-0.04, 0.20)	0.42	0.22
Adults	22 (13413)	0.01 (-0.06, 0.07)	< 0.001	0.81
Children/adolescents	5 (1529)	-0.01 (-0.15, 0.13)	0.23	0.88
European Caucasian	9 (3015)	0.01 (-0.11, 0.13)	0.02	0.85
East Asian	9 (9848)	0.03 (-0.06, 0.12)	0.001	0.50
West Asian	6 (1273)	-0.01 (-0.18, 0.16)	0.07	0.89
Overweight/obesity patients	14 (3192)	0.02 (-0.09, 0.13)	0.03	0.72
T2DM patients	5 (3912)	0.03 (-0.03, 0.10)	0.64	0.33
Hypertensive patients	3 (1696)	0.02 (-0.08, 0.12)	0.83	0.74
General/control subjects	6 (2925)	0.09 (-0.03, 0.21)	0.08	0.15
HDL-C (CC + CT vs. TT)				
All subjects	28 (20998)	-0.01 (-0.06, 0.03)	0.04	0.51
Females	8 (2931)	-0.05 (-0.14, 0.04)	0.27	0.26
Males	4 (1108)	0.05 (-0.07, 0.17)	0.40	0.42
Adults	23 (19464)	-0.02 (-0.06, 0.02)	0.17	0.32
Children/adolescents	5 (1534)	0.01 (-0.20, 0.22)	0.01	0.91
European Caucasian	10 (8827)	0.002 (-0.08, 0.08)	0.10	0.96
East Asian	9 (10092)	-0.05 (-0.10, < 0.001)	0.20	0.052
West Asian	6 (1273)	0.06 (-0.06, 0.17)	0.78	0.34
Overweight/obesity patients	14 (3197)	0.04 (-0.03, 0.11)	0.49	0.25
T2DM patients	4 (2121)	-0.07 (-0.16, 0.02)	0.94	0.11
Hypertensive patients	3 (1696)	-0.08 (-0.18, 0.02)	0.84	0.14
General/control subjects	8 (10767)	-0.04 (-0.13, 0.05)	0.003	0.40

MC4R, melanocortin 4 receptor; SMD, standardized mean difference; 95% CI, 95% confidence interval; TG, triglyceride; TC, total cholesterol; LDL-C, low-density lipoprotein cholesterol; HDL-C, high-density lipoprotein cholesterol; T2DM, type 2 diabetes mellitus.

The interactions of the rs17782313 polymorphism with age, ethnicity and health condition on the indexes of obesity and glucometabolic disorder were observed (**Tables 1**, **2**). The C allele of the rs17782313 variant is correlated with a higher level of WC (SMD = 0.17 cm, 95% CI = 0.08 to 0.26 cm, *p* < 0.001) or GLU (SMD = 0.10 mg/dL, 95% CI = 0.02 to 0.19 mg/dL, *p* = 0.02) in adults, but not in children/adolescents. The C allele of the rs17782313 polymorphism is associated with a higher level of BMI in European Caucasians (SMD = 0.18 kg/m^2^, 95% CI = 0.05 to 0.31 kg/m^2^, *p* = 0.01) and East Asians (SMD = 0.11 kg/m^2^, 95% CI = 0.05 to 0.17 kg/m^2^, *p* < 0.001), but not in South Americans and West Asians. Similarly, the C allele of the rs17782313 polymorphism is associated with higher levels of WC (SMD = 0.19 cm, 95% CI = 0.06 to 0.32 cm, *p* = 0.004) and GLU (SMD = 0.15 mg/dL, 95% CI = 0.01 to 0.29 mg/dL, *p* = 0.03) in European Caucasians, but not in any other ethnicities. The C allele of the rs17782313 polymorphism is associated with a higher level of WHR (SMD = 0.17, 95% CI = 0.03 to 0.30, *p* = 0.02) in West Asians, but not in the whole population or any other ethnicities. The C allele of the rs17782313 polymorphism is associated with a higher level of BMI in T2DM patients (SMD = 0.08 kg/m^2^, 95% CI = 0.01 to 0.16 kg/m^2^, *p* = 0.03), hypertensive patients (SMD = 0.08 kg/m^2^, 95% CI = 0.04 to 0.13 kg/m^2^, *p* < 0.001) and general/control subjects (SMD = 0.33 kg/m^2^, 95% CI = 0.17 to 0.50 kg/m^2^, *p* < 0.001), but not in overweight/obesity patients. Notably, the C allele of the rs17782313 variant is correlated with a higher level of WC (SMD = 0.24 cm, 95% CI = 0.05 to 0.42 cm, *p* = 0.01) or GLU (SMD = 0.15 mg/dL, 95% CI = 0.03 to 0.27 mg/dL, *p* = 0.01) in general/control subjects, but not in any of the patient populations.

### Associations of the PGC1α rs8192678 polymorphism with the indexes of obesity, glucometabolic disorder and dyslipidemia

As indicated in **Tables 4**–**6** and **Figures S8**-**S17**, there are no significant correlations being detected between the rs8192678 polymorphism and any of the ten variables of obesity, glucometabolic disorder and dyslipidemia in the pooled meta-analyses in the overall population. However, significant associations between the rs8192678 polymorphism and glucometabolic disorder indexes as well as LDL-C were found in the subgroup analyses (**Tables 5**, **6****)**. The A-allele carriers of the rs8192678 polymorphism had a higher level of GLU in East Asians (SMD = 0.11 mg/dL, 95% CI = 0.01 to 0.20 mg/dL, *p* = 0.03) and T2DM patients (SMD = 0.17 mg/dL, 95% CI = 0.04 to 0.29 mg/dL, *p* = 0.01), a higher level of INS in South Americans (SMD = 0.20 μU/mL, 95% CI = 0.01 to 0.39 μU/mL, *p* = 0.04) and overweight/obesity patients (SMD = 0.11 μU/mL, 95% CI = 0.02 to 0.20 μU/mL, *p* = 0.02), and a higher level of HOMA-IR in children/adolescents (SMD = 0.14, 95% CI = 0.01 to 0.26, *p* = 0.03), overweight/obesity patients (SMD = 0.09, 95% CI = 0.001 to 0.18, *p* = 0.049) as well as T2DM patents (SMD = 0.19, 95% CI = -0.001 to 0.38, *p* = 0.05) than the GG homozygotes. Somehow, the A-allele carriers of the rs8192678 polymorphism had a lower level of INS than the GG homozygotes in females (SMD = -0.35 μU/mL, 95% CI = -0.55 to -0.15 μU/mL, *p* = 0.001) and European Caucasians (SMD = -0.07 μU/mL, 95% CI = -0.13 to 0.001 μU/mL, *p* = 0.05). Notably, the A-allele carriers of the rs8192678 polymorphism were found to have a higher level of LDL-C (SMD = 0.12 mg/dL, 95% CI = 0.04 to 0.20 mg/dL, *p* < 0.01) than the GG homozygotes in East Asians.

**Table 4:** Meta-analyses between the PGC1α rs8192678 polymorphism and obesity indexes.

Groups or subgroups	Comparisons (Subjects)	SMD (95% CI)	*P*_Heterogeneity_	*P*_SMD_
BMI (AA + AG vs. GG)				
All subjects	64 (15755)	0.01 (-0.04, 0.05)	0.01	0.78
Males	6 (1319)	0.005 (-0.11, 0.12)	0.43	0.93
Females	10 (1810)	-0.03 (-0.13, 0.07)	0.63	0.53
Adults	58 (13125)	0.001 (-0.05, 0.05)	0.01	0.97
Children/adolescents	6 (2630)	0.04 (-0.05, 0.13)	0.33	0.40
European Caucasian	15 (6272)	0.04 (-0.04, 0.11)	0.06	0.35
South American	3 (430)	-0.03 (-0.22, 0.16)	0.80	0.74
East Asian	37 (7218)	-0.005 (-0.08, 0.07)	0.004	0.89
West Asian	6 (1447)	-0.06 (-0.17, 0.05)	0.99	0.30
Overweight/obesity patients	12 (2474)	0.04 (-0.05, 0.12)	0.88	0.40
T2DM patients	12 (1613)	0.03 (-0.09, 0.16)	0.25	0.63
PCOS patients	4 (536)	-0.14 (-0.34, 0.06)	0.72	0.16
General/control subjects	28 (9622)	0.03 (-0.03, 0.10)	0.02	0.27
WC (AA + AG vs. GG)				
All subjects	16 (6543)	0.02 (-0.03, 0.07)	0.96	0.39
Males	3 (164)	-0.05 (-0.38, 0.28)	0.98	0.76
Adults	13 (4773)	-0.01 (-0.06, 0.05)	0.99	0.88
Children/adolescents	3 (1770)	0.10 (-0.002, 0.19)	0.58	0.06
European Caucasian	6 (3297)	0.02 (-0.05, 0.09)	0.43	0.64
South American	3 (430)	-0.05 (-0.24, 0.14)	0.70	0.59
East Asian	5 (2546)	0.04 (-0.04, 0.13)	0.99	0.32
Overweight/obesity patients	8 (1639)	-0.01 (-0.11, 0.09)	0.94	0.84
General/control subjects	6 (3537)	0.03 (-0.04, 0.10)	0.58	0.40
WHR (AA + AG vs. GG)				
All subjects	21 (5067)	-0.01 (-0.07, 0.05)	0.44	0.80
Adults	20 (4286)	-0.001 (-0.07, 0.07)	0.40	0.98
South American	3 (430)	-0.07 (-0.28, 0.14)	0.31	0.53
East Asian	15 (3665)	0.01 (-0.07, 0.09)	0.28	0.89
Overweight/obesity patients	6 (1716)	-0.05 (-0.25, 0.15)	0.01	0.63
T2DM patients	5 (847)	-0.04 (-0.30, 0.23)	0.03	0.79
General/control subjects	9 (2323)	-0.02 (-0.11, 0.07)	1.00	0.69

PGC1α, peroxisome proliferator activated receptor gamma coactivator 1 alpha; SMD, standardized mean difference; 95% CI, 95% confidence interval; BMI, body mass index; WC, waist circumference; WHR, waist-to-hip ratio; T2DM, type 2 diabetes mellitus; PCOS, polycystic ovary syndrome.

**Table 5:** Meta-analyses between the PGC1α rs8192678 polymorphism and glucometabolic indexes.

Groups or subgroups	Comparisons (Subjects)	SMD (95% CI)	*P*_Heterogeneity_	*P*_SMD_
GLU (AA + AG vs. GG)				
All subjects	43 (10642)	0.01 (-0.01, 0.12)	< 0.001	0.86
Females	3 (521)	-0.05 (-0.39, 0.29)	0.07	0.78
Males	3 (210)	-0.01 (-0.31, 0.29)	0.77	0.96
Adults	39 (9220)	0.02 (-0.11, 0.14)	< 0.001	0.80
Children/adolescents	4 (1422)	-0.06 (-0.24, 0.13)	0.09	0.56
European Caucasian	10 (4450)	-0.05 (-0.16, 0.06)	0.02	0.36
South American	4 (677)	-0.05 (-0.30, 0.20)	0.07	0.68
East Asian	22 (4380)	0.11 (0.01, 0.20)	0.01	0.03
West Asian	5 (865)	-0.64 (-1.72, 0.44)	< 0.001	0.24
Overweight/obesity patients	10 (2045)	0.03 (-0.13, 0.20)	0.02	0.69
T2DM patients	9 (1220)	0.17 (0.04, 0.29)	0.56	0.01
General/control subjects	18 (6389)	0.04 (-0.06, 0.14)	< 0.001	0.45
INS (AA + AG vs. GG)				
All subjects	30 (9109)	0.05 (-0.04, 0.13)	< 0.001	0.29
Females	3 (491)	-0.35 (-0.55, -0.15)	0.70	0.001
Adults	28 (8042)	0.04 (-0.06, 0.13)	< 0.001	0.45
European Caucasian	4 (3552)	-0.07 (-0.13, 0.001)	0.89	0.05
South American	3 (430)	0.20 (0.01, 0.39)	0.98	0.04
East Asian	20 (4552)	0.03 (-0.10, 0.15)	< 0.001	0.67
West Asian	3 (575)	0.21 (-0.17, 0.58)	0.05	0.28
Overweight/obesity patients	8 (2073)	0.11 (0.02, 0.20)	0.94	0.02
T2DM patients	6 (796)	-0.02 (-0.25, 0.22)	0.07	0.88
General/control subjects	13 (5788)	0.02 (-0.10, 0.15)	< 0.001	0.71
HOMA-IR (AA + AG vs. GG)				
All subjects	25 (7706)	0.04 (-0.05, 0.13)	< 0.001	0.34
Adults	22 (6531)	0.03 (-0.07, 0.13)	< 0.001	0.53
Children/adolescents	3 (1175)	0.14 (0.01, 0.26)	0.73	0.03
South American	3 (430)	0.14 (-0.05, 0.33)	0.79	0.16
East Asian	17 (4266)	0.01 (-0.11, 0.12)	0.001	0.93
West Asian	3 (575)	0.25 (-0.08, 0.58)	0.08	0.14
Overweight/obesity patients	8 (2073)	0.09 (0.001, 0.18)	0.97	0.049
T2DM patients	3 (513)	0.19 (-0.001, 0.38)	0.74	0.05
General/control subjects	10 (4560)	-0.04 (-0.16, 0.09)	0.01	0.58

PGC1α, peroxisome proliferator activated receptor gamma coactivator 1 alpha; SMD, standardized mean difference; 95% CI, 95% confidence interval; GLU, glucose; INS, insulin; HOMA-IR, homeostasis model assessment of insulin resistance; T2DM, type 2 diabetes mellitus.

**Table 6:** Meta-analyses between the PGC1α rs8192678 polymorphism and dyslipidemia indexes.

Groups or subgroups	Comparisons (Subjects)	SMD (95% CI)	*P*_Heterogeneity_	*P*_SMD_
TG (AA + AG vs. GG)				
All subjects	47 (10685)	-0.04 (-0.17, 0.09)	< 0.001	0.53
Females	3 (521)	-0.15 (-0.34, 0.05)	0.45	0.14
Males	3 (210)	0.07 (-0.23, 0.37)	0.81	0.63
Adults	43 (9402)	-0.06 (-0.20, 0.08)	< 0.001	0.37
Children/adolescents	4 (1283)	0.16 (-0.07, 0.39)	0.05	0.18
European Caucasian	11 (4269)	-0.01 (-0.07, 0.05)	0.77	0.84
South American	4 (538)	0.38 (-0.31, 1.08)	< 0.001	0.28
East Asian	25 (4743)	0.08 (-0.02, 0.17)	0.001	0.13
West Asian	5 (865)	-1.20 (-2.52, 0.12)	< 0.001	0.07
Overweight/obesity patients	12 (2236)	0.10 (-0.12, 0.32)	< 0.001	0.37
T2DM patients	11 (1686)	0.11 (-0.04, 0.26)	0.06	0.16
General/control subjects	17 (5200)	-0.11 (-0.33, 0.11)	< 0.001	0.34
TC (AA + AG vs. GG)				
All subjects	45 (10625)	0.01 (-0.12, 0.14)	< 0.001	0.90
Females	3 (521)	-0.23 (-0.52, 0.06)	0.13	0.12
Adults	42 (9450)	0.04 (-0.01, 0.17)	< 0.001	0.61
Children/adolescents	3 (1175)	-0.34 (-0.81, 0.13)	< 0.001	0.16
European Caucasian	10 (4245)	-0.04 (-0.16, 0.08)	0.01	0.49
South American	3 (430)	-0.07 (-0.26, 0.12)	0.75	0.46
East Asian	25 (4815)	0.02 (-0.09, 0.13)	< 0.001	0.68
West Asian	5 (865)	0.27 (-1.21, 1.75)	< 0.001	0.72
Overweight/obesity patients	11 (2212)	-0.03 (-0.13, 0.08)	0.33	0.64
T2DM patients	11 (1686)	0.06 (-0.08, 0.21)	0.09	0.39
General/control subjects	16 (5092)	-0.02(-0.22, 0.17)	< 0.001	0.81
LDL-C (AA + AG vs. GG)				
All subjects	36 (8010)	0.08 (-0.04, 0.20)	< 0.001	0.18
Adults	35 (7902)	0.08 (-0.04, 0.20)	< 0.001	0.18
European Caucasian	7 (3292)	0.27 (-0.16, 0.71)	< 0.001	0.22
South American	4 (538)	0.02 (-0.15, 0.19)	0.98	0.84
East Asian	18 (3045)	0.12 (0.04, 0.20)	0.38	< 0.01
West Asian	5 (865)	-0.12 (-0.46, 0.23)	< 0.001	0.52
Overweight/obesity patients	10 (1431)	-0.05 (-0.16, 0.07)	0.47	0.44
T2DM patients	9 (1531)	0.02 (-0.09, 0.13)	0.62	0.75
General/control subjects	13 (4001)	0.18 (-0.01, 0.37)	< 0.001	0.07
HDL-C (AA + AG vs. GG)				
All subjects	45 (10317)	-0.03 (-0.10, 0.04)	< 0.001	0.36
Females	3 (521)	0.22 (-0.19, 0.64)	0.02	0.29
Males	3 (210)	0.01 (-0.40, 0.42)	0.19	0.98
Adults	42 (9142)	-0.04 (-0.11, 0.04)	< 0.001	0.34
Children/adolescents	3 (1175)	0.05 (-0.30, 0.40)	0.01	0.78
European Caucasian	9 (3807)	0.02 (-0.09, 0.12)	0.13	0.78
South American	3 (430)	-0.02 (-0.24, 0.20)	0.29	0.87
East Asian	26 (4945)	-0.05 (-0.17, 0.06)	< 0.001	0.35
West Asian	5 (865)	-0.09 (-0.24, 0.06)	0.59	0.22
Overweight/obesity patients	12 (2236)	-0.02 (-0.15, 0.11)	0.12	0.76
T2DM patients	11 (1686)	-0.08 (-0.20, 0.04)	0.27	0.20
General/control subjects	16 (5128)	-0.07 (-0.19, 0.04)	0.001	0.21

PGC1α, peroxisome proliferator activated receptor gamma coactivator 1 alpha; SMD, standardized mean difference; 95% CI, 95% confidence interval; TG, triglyceride; TC, total cholesterol; LDL-C, low-density lipoprotein cholesterol; HDL-C, high-density lipoprotein cholesterol; T2DM, type 2 diabetes mellitus.

### Heterogeneity analysis

Significant heterogeneity was found in the pooled meta-analyses for the rs17782313 and rs8192678 polymorphisms in the overall population (**Tables 1****-** **6**). Sources of heterogeneity were successfully identified by using Galbraith plots. The heterogeneity was effectively removed or decreased after excluding the outlier studies, while the results of the pooled meta-analyses were not altered significantly except the association of the rs17782313 polymorphism with WHR, which became significant when the outlier studies were excluded (SMD = 0.15, 95% CI = 0.09 to 0.22, *P*_Heterogeneity_ = 0.24, *P*_SMD_ < 0.001).

### Publication bias

Publication bias was observed in the association analysis between the rs17782313 variant and BMI, as well as between the rs8192678 polymorphism and GLU or TG. However, no significant changes were found for all after adjustment by the trim-and-fill method.

## Discussion

MC4R and PGC1α play critical roles in energy homeostasis, and are implicated in the pathogenesis of metabolic diseases including obesity, diabetes, hepatic steatosis and CVD (73–80). A number of scientific reports have suggested that the MC4R rs17782313 and PGC1α rs8192678 polymorphisms are associated with the onset of T2DM and CVD (81–86), which prompted us to conduct this meta-analysis to clarify the relationships of the two polymorphic variants with the indexes of obesity, glucometabolic disorder and dyslipidemia since all of these indexes are closely related to T2DM and CVD. The present meta-analysis demonstrates that the C allele of the rs17782313 polymorphism near MC4R is associated with a higher level of BMI, WC or GLU, but not with blood lipids, which is in agreement with the previous findings that the C allele of the rs17782313 variant is strongly correlated with an elevated risk of obesity and T2DM (87–89), but weakly with CVD (90). In addition, we found that the PGC1α rs8192678 polymorphism is associated with glucometabolic disorder indexes in some specific populations such as East and West Asians, Europeans, and overweight/obesity and T2DM patients although this variant is not correlated with the indexes of obesity, dyslipidemia and glucometabolic disorder in the pooled meta-analyses in the overall population.

Although the rs17782313 polymorphism is just localized near, but not within the MC4R gene, its impact on obesity risk seems to be greater than those polymorphic loci located in the MC4R gene. There were many more studies in the literature reporting the effect of the rs17782313 polymorphism on obesity risk compared to several MC4R exonic polymorphisms such as V103I, I251L, S127L and K73R. Nearly 90% of the studies reported that the rs17782313 polymorphism has a significant impact on obesity risk, while only 50% of the studies on average reported that MC4R exonic polymorphisms are significantly related to obesity risk (data not shown). Interestingly, another polymorphic site near the MC4R gene, rs12970134, was also found to be strongly associated with obesity risk, with 24 studies out of 25 studies reporting a significant impact of the rs12970134 polymorphism on obesity risk (data not shown).

The mechanisms underlying the correlations of the rs17782313 polymorphism with obesity as well as hyperglycemia may be that this variant leads to abnormal expression of MC4R as it is located downstream of the MC4R gene without any amino acid alteration in MC4R receptor. Indeed, Tang and colleagues (91) conducted MeQTL and eQTL analyses to explore the effects of the rs17782313 polymorphism on DNA methylation and MC4R expression, and found that the C allele of the rs17782313 polymorphism is associated with a decreased methylation state in the promoter region of MC4R (*p* = 1.7 × 10^-4^) and an increased mRNA expression level of MC4R (*p* = 1.9 × 10^-3^). We used GTEx Analysis Release V8 (dbGaP Accession phs000424.v8.p2) to assess the impact of the rs17782313 polymorphism on the mRNA expression pattern of MC4R, and the results displayed that the MC4R mRNA level increases orderly with the TT, TC and CC genotypes in brain nucleus accumbens and putamen, but decreases orderly in cerebellum (data not shown, *p* < 0.05 for all). However, Lauria et al. (92) did not detect a significant link between the rs17782313 polymorphism and the MC4R mRNA expression level in peripheral blood cells. The relation between the rs17782313 polymorphism and MC4R expression pattern remains to be examined. Several studies have shown that MC4R can promote the expression of brain-derived neurotrophic factor (BDNF), and activate the AMPK-SIRT1-PGC-1α pathway (93–95). Therefore, BDNF and sirtuin 1 may mediate the association between the MC4R rs17782313 polymorphism and obesity as well as hyperglycemia, which requires further investigation to confirm.

The PGC1α rs8192678 polymorphism has also been shown to affect the expression and function of PGC-1α. Taghvaei et al. (96) demonstrated that the substitution of glycine with serine at the rs8192678 polymorphism has a destabilizing effect on PGC-1α by computational analysis. The influences of multiple genetic and environmental factors on PGC-1α expression in skeletal muscles were investigated in Swedish twins, and the researchers observed that PGC-1α gene expression decreased with age and was modulated by the rs8192678 polymorphism (97). Chen et al. (98) explored the direct role of the rs8192678 polymorphism in altering the actions of PGC-1α on hepatocyte fat deposition and found that the hepatocytes with the Ser/Ser genotype exhibited a significant elevation in palmitate-induced fat deposition compared to the hepatocytes with the Gly/Gly genotype. In addition, the Ser/Ser genotype of the rs8192678 polymorphism caused a significant decrease in the expression of carnitine palmitoyl transferase 1a, a rate-limiting enzyme in fatty acid oxidation that transfers long-chain fatty acids into mitochondria for oxidation (98).

Significant heterogeneity was existed in the pooled meta-analyses of the rs17782313 polymorphism with BMI, WC and GLU. The outlier studies were figured out by observing Galbraith plots. No significant alterations in SMDs as well as 95% CIs were observed after removing the outlier studies, which indicates that the associations between the rs17782313 variant and these indexes are very robust. WHR, an indicator of abdominal obesity, was shown to be significantly associated with the rs17782313 polymorphism after exclusion of the outlier studies. It suggests that the significant correlation between the rs17782313 polymorphism and WHR has been masked by the extensive heterogeneity among studies. Significant heterogeneity was also detected in the association analyses between the rs8192678 polymorphism and the indexes of obesity, glucometabolic disorder and dyslipidemia. The outlier studies were identified and excluded, but there were still no significant correlations between the rs8192678 polymorphism and all of these indexes in the whole population, which indicates that the associations of the rs8192678 polymorphism with obesity, diabetes and dyslipidemia are weak, although PGC1α works as an activator of PPARγ, an important modulator of glucose and lipid metabolism (7).

There are several limitations to current study. Firstly, this meta-analysis only included the studies that published in Chinese or English as it is difficult to obtain the full articles written in various languages. Secondly, the interactions of the rs17782313 and rs8192678 polymorphisms with other genetic variants or non-genetic factors other than age, gender, ethnicity and health condition on metabolic abnormalities were not investigated in this meta-analysis due to lack of original data from the enrolled studies. A large number of genes, diets, physical activities and environmental factors are implicated in the pathogenesis of metabolic disorders such as dyslipidemia, diabetes and obesity. More precise results could be obtained if gene-gene interactions were explored, or stratification analyses according to diets, physical activities and environmental factors were conducted.

In conclusion, the C allele of the rs17782313 polymorphism near MC4R confers a higher risk of obesity and hyperglycemia, and the A allele of the rs8192678 polymorphism is associated with glucometabolic disorder in some specific populations. These findings can provide a direct link between the rs17782313 and rs8192678 polymorphisms and T2DM as well as CVD.

## Data availability statement

The original contributions presented in the study are included in the article/**Supplementary Material**. Further inquiries can be directed to the corresponding author.

## Author contributions

YS, YZ and SL conceived of the systematic review and meta-analysis, participated in the design, analyzed the data, and drafted the manuscript. HN, XW, XL, JW and ML carried out the literature search, collected the data, and revised the manuscript critically for important intellectual content. All authors contributed to the article and approved the submitted version.
