# Supplementary material for: The rs17782313 polymorphism near MC4R gene confers a high risk of obesity and hyperglycemia, while PGC1α rs8192678 polymorphism is weakly correlated with glucometabolic disorder: a systematic review and meta-analysis
Source: Front Endocrinol (Lausanne). 2023 Aug 9;14:1210455. doi: 10.3389/fendo.2023.1210455 (PMC10445758; doi:10.3389/fendo.2023.1210455)
Supplement: Supplementary file 1 [file Image_1.pdf]

Supplementary Figures S1-S17

Figure S1 Forest plot of the association analysis between the MC4R rs17782313 polymorphism and waist-to-hip ratio (WHR).

Figure S2 Forest plot of the association analysis between the MC4R rs17782313 polymorphism and blood levels of insulin (INS).

Figure S3 Forest plot of the association analysis between the MC4R rs17782313 polymorphism and homeostasis model assessment of insulin resistance (HOMA-IR).

Figure S4 Forest plot of the association analysis between the MC4R rs17782313 polymorphism and blood levels of triglycerides (TG).

Figure S5 Forest plot of the association analysis between the MC4R rs17782313 polymorphism and blood levels of total cholesterol (TC).

Figure S6 Forest plot of the association analysis between the MC4R rs17782313 polymorphism and blood levels of low-density lipoprotein cholesterol (LDL-C).

Figure S7 Forest plot of the association analysis between the MC4R rs17782313 polymorphism and blood levels of high-density lipoprotein cholesterol (HDL-C).

Figure S8 Forest plot of the association analysis between the rs8192678 polymorphism and body mass index (BMI).

Figure S9 Forest plot of the association analysis between the PGC1α rs8192678 polymorphism and waist circumference (WC).

Figure S10 Forest plot of the association analysis between the PGC1α rs8192678 polymorphism and waist-to-hip ratio (WHR).

Figure S11 Forest plot of the association analysis between the PGC1α rs8192678 polymorphism and blood levels of glucose (GLU).

Figure S12 Forest plot of the association analysis between the PGC1α rs8192678 polymorphism

and blood levels of insulin (INS).

Figure S13 Forest plot of the association analysis between the PGC1α rs8192678 polymorphism and homeostasis model assessment of insulin resistance (HOMA-IR).

Figure S14 Forest plot of the association analysis between the PGC1α rs8192678 polymorphism and blood levels of triglycerides (TG).

Figure S15 Forest plot of the association analysis between the PGC1α rs8192678 polymorphism and blood levels of total cholesterol (TC).

Figure S16 Forest plot of the association analysis between the PGC1α rs8192678 polymorphism and blood levels of low-density lipoprotein cholesterol (LDL-C).

Figure S17 Forest plot of the association analysis between the PGC1α rs8192678 polymorphism and blood levels of high-density lipoprotein cholesterol (HDL-C).

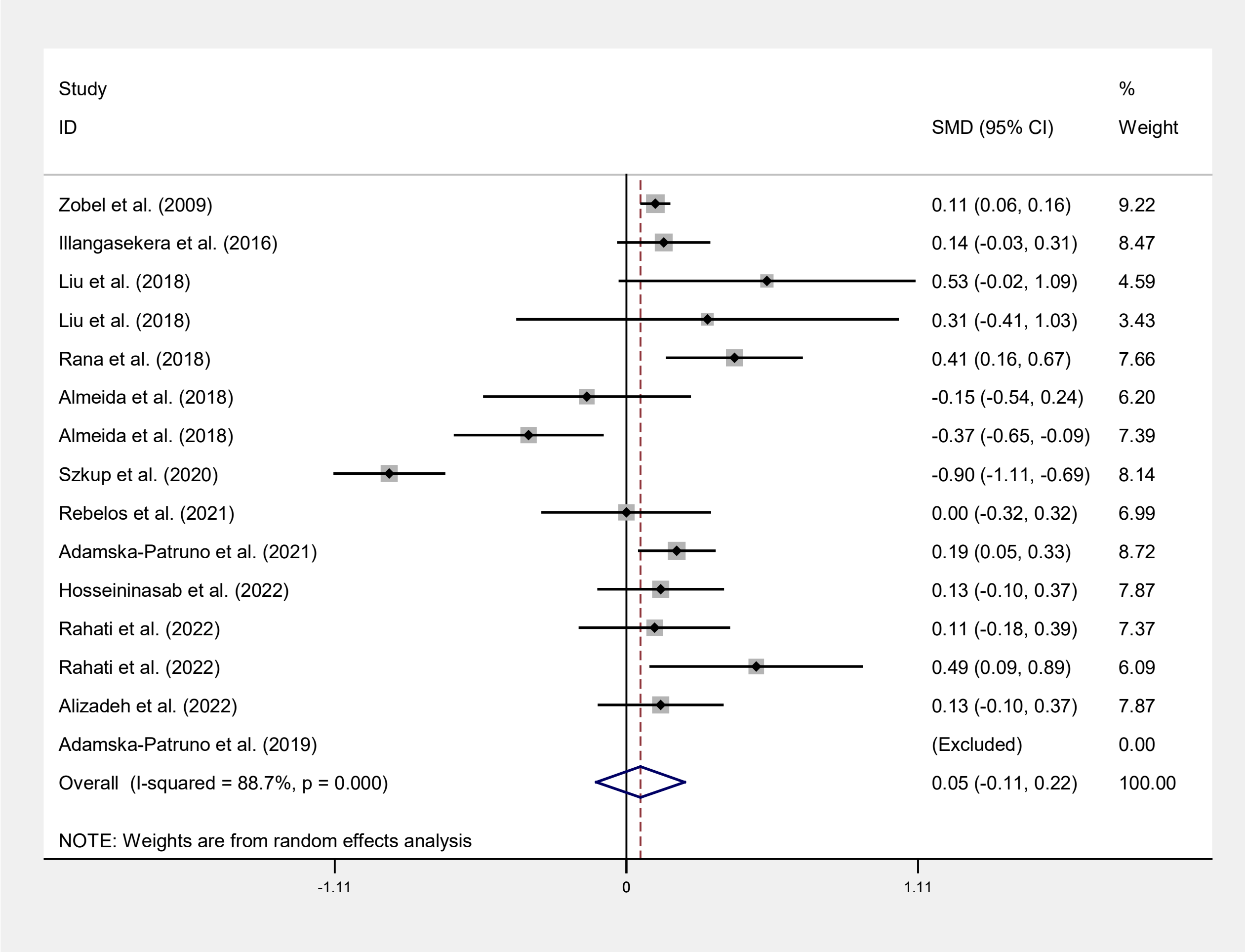

Figure S1. Forest plot of the association analysis between the MC4R rs17782313 polymorphism and waist-to-hip ratio (WHR).

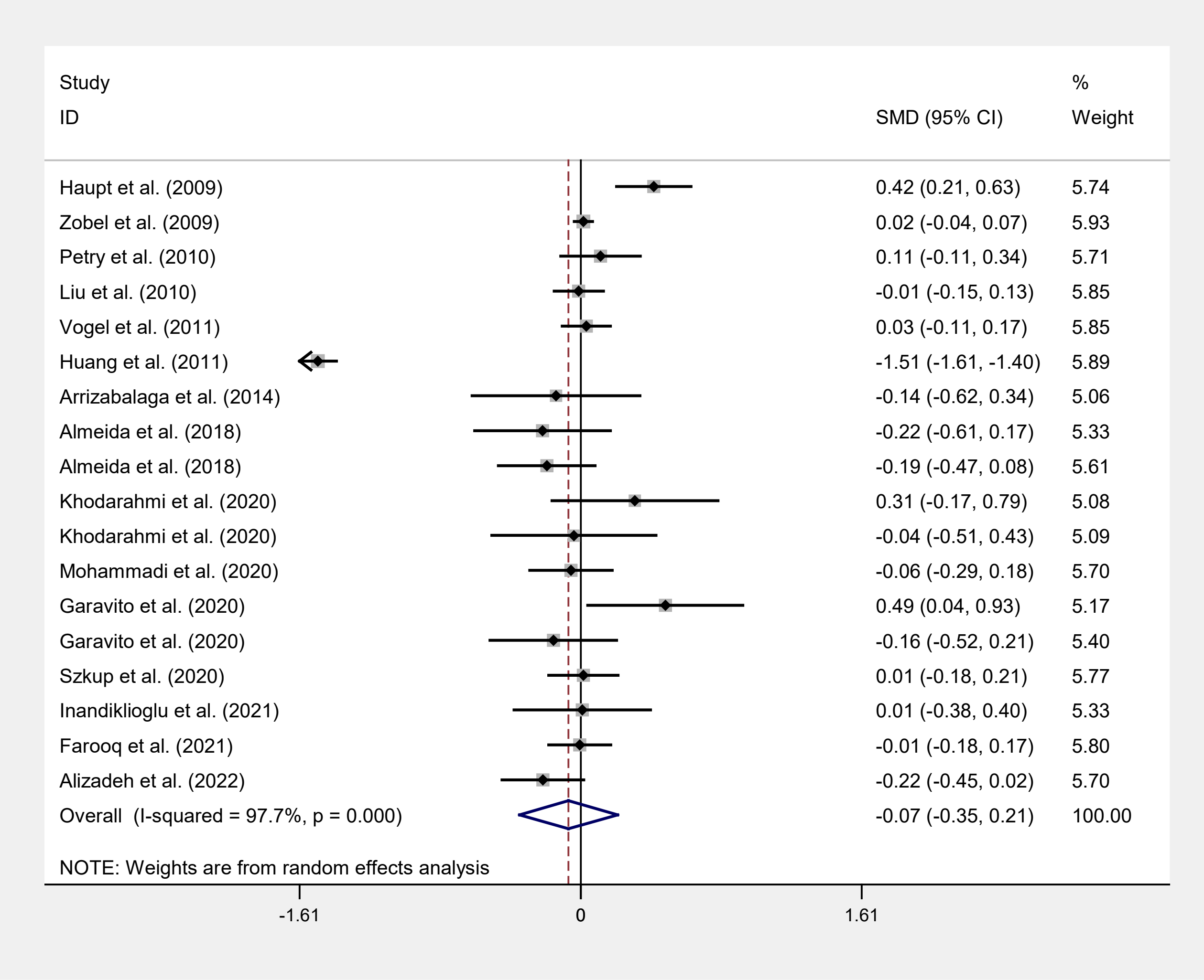

Figure S2. Forest plot of the association analysis between the MC4R rs17782313 polymorphism and blood levels of insulin (INS).

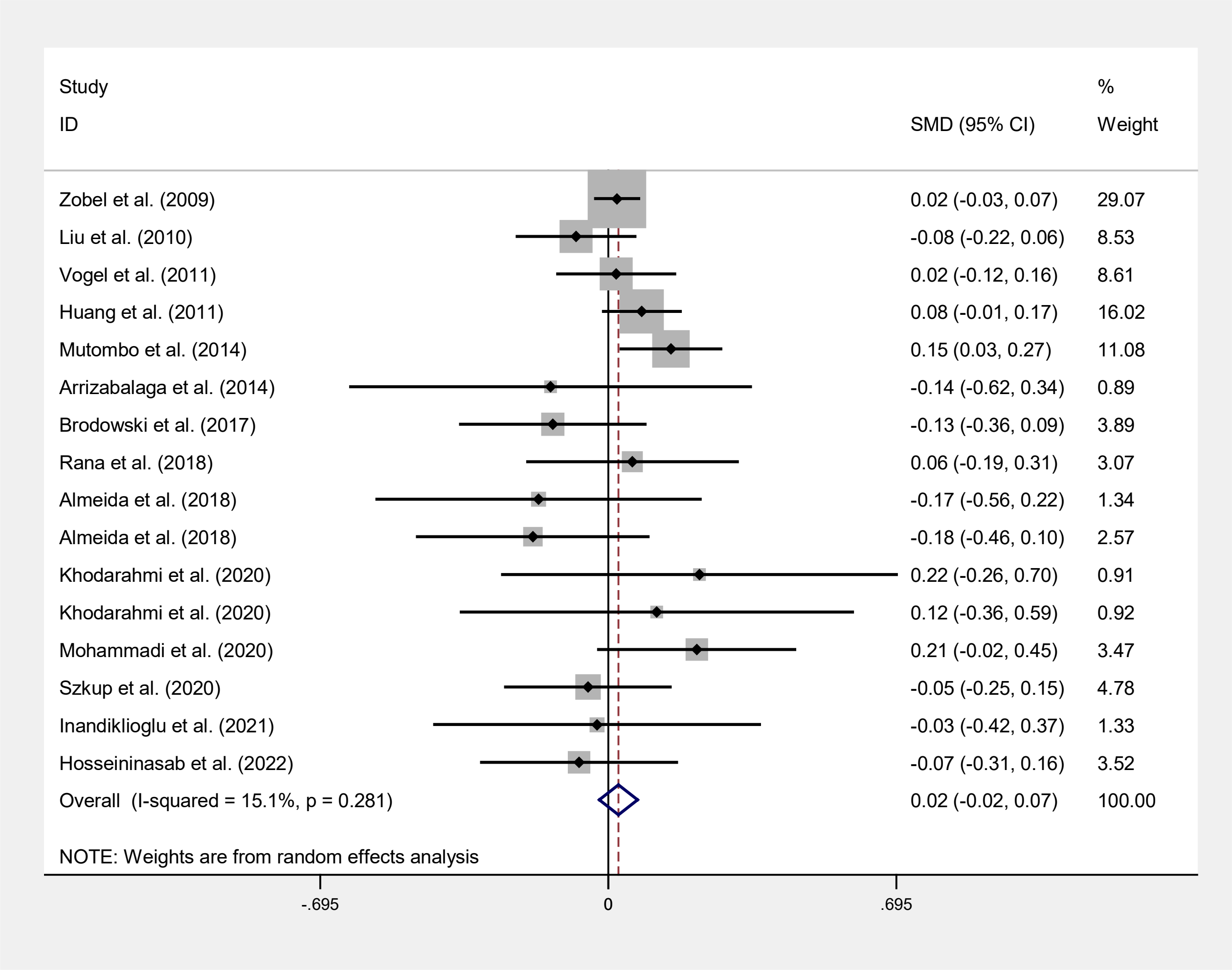

Figure S3. Forest plot of the association analysis between the MC4R rs17782313 polymorphism and homeostasis model assessment of insulin resistance (HOMA-IR).

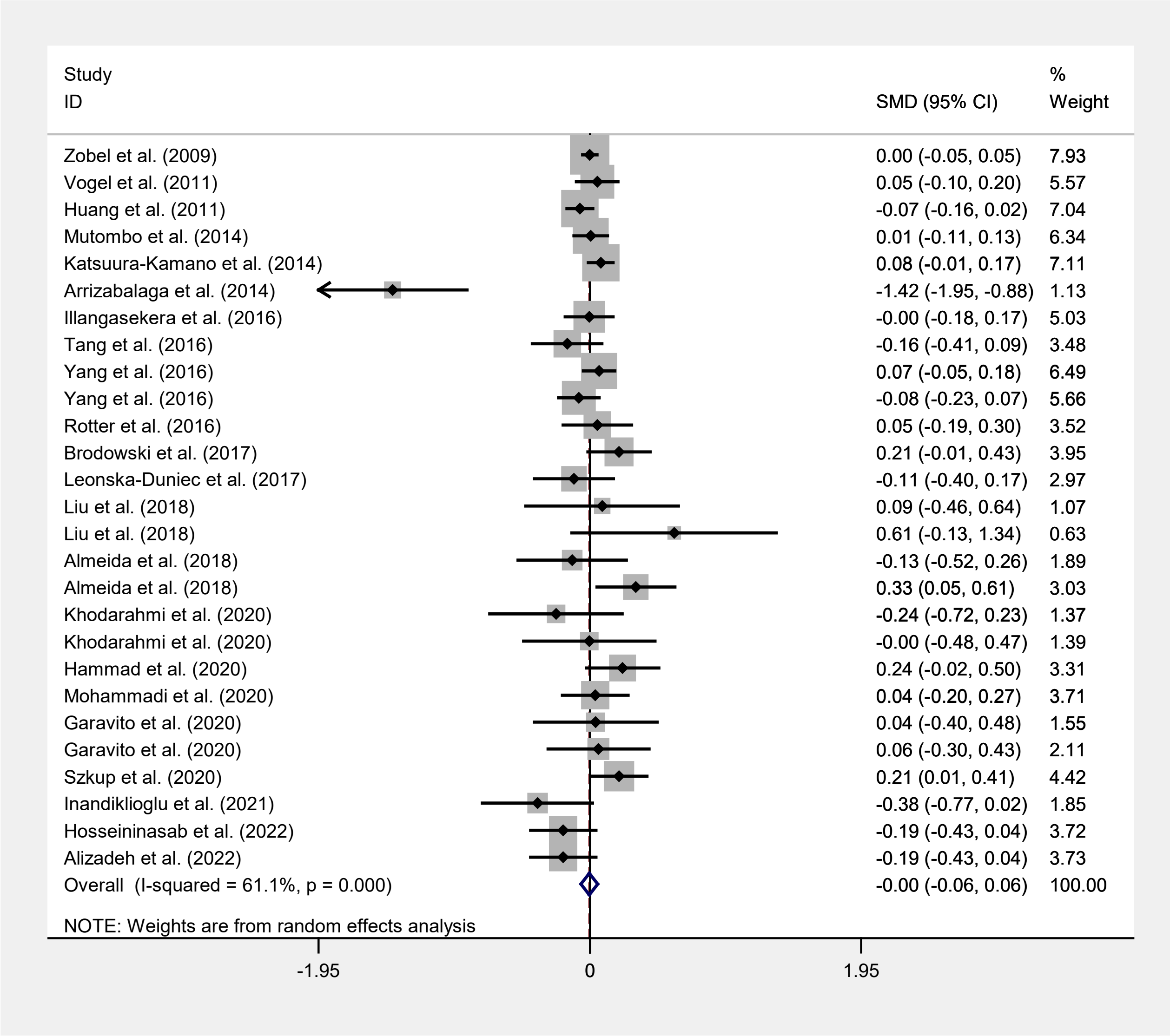

Figure S4. Forest plot of the association analysis between the MC4R rs17782313 polymorphism and blood levels of triglycerides (TG).

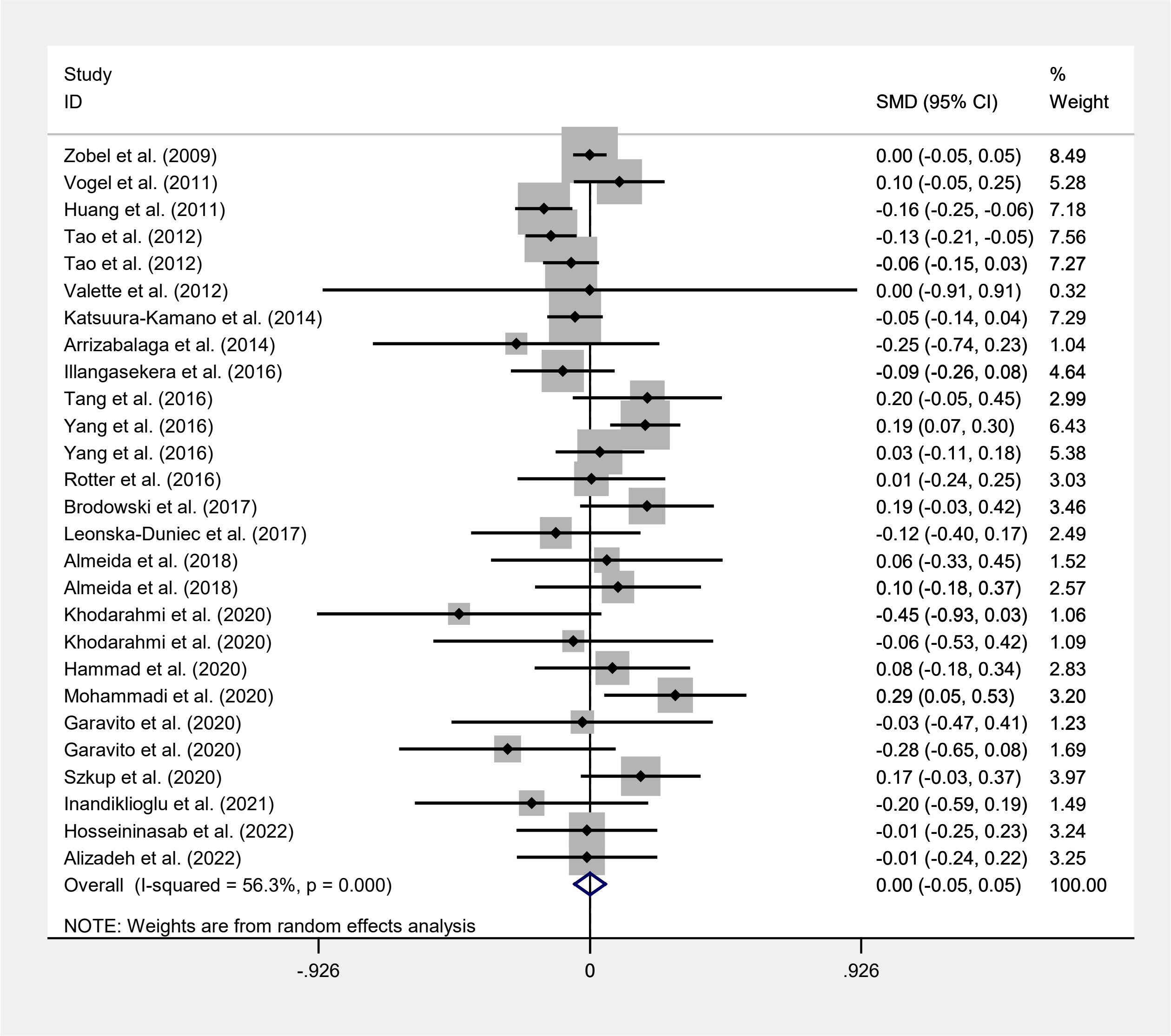

Figure S5. Forest plot of the association analysis between the MC4R rs17782313 polymorphism and blood levels of total cholesterol (TC).

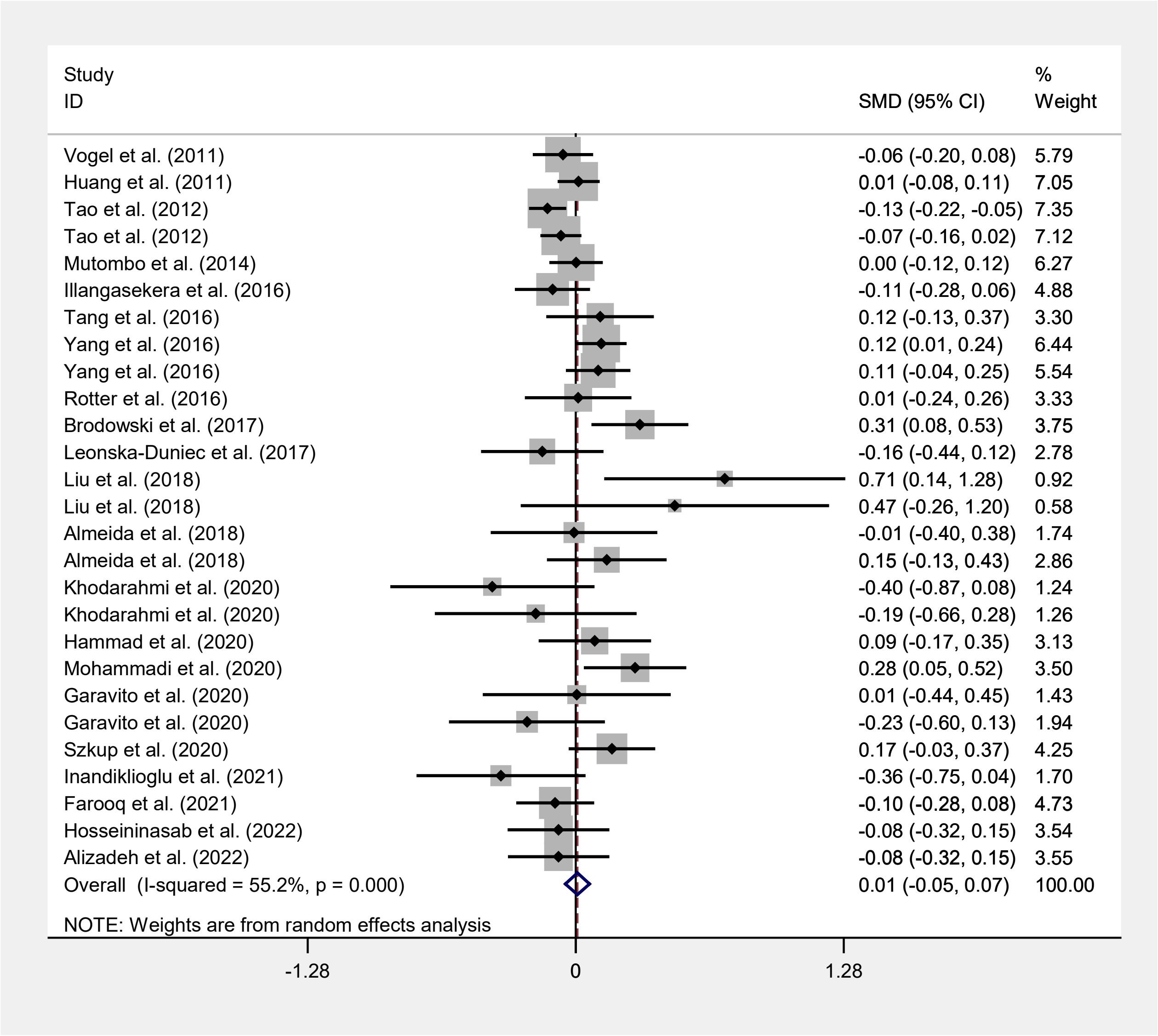

Figure S6. Forest plot of the association analysis between the MC4R rs17782313 polymorphism and blood levels of low-density lipoprotein cholesterol (LDL-C).

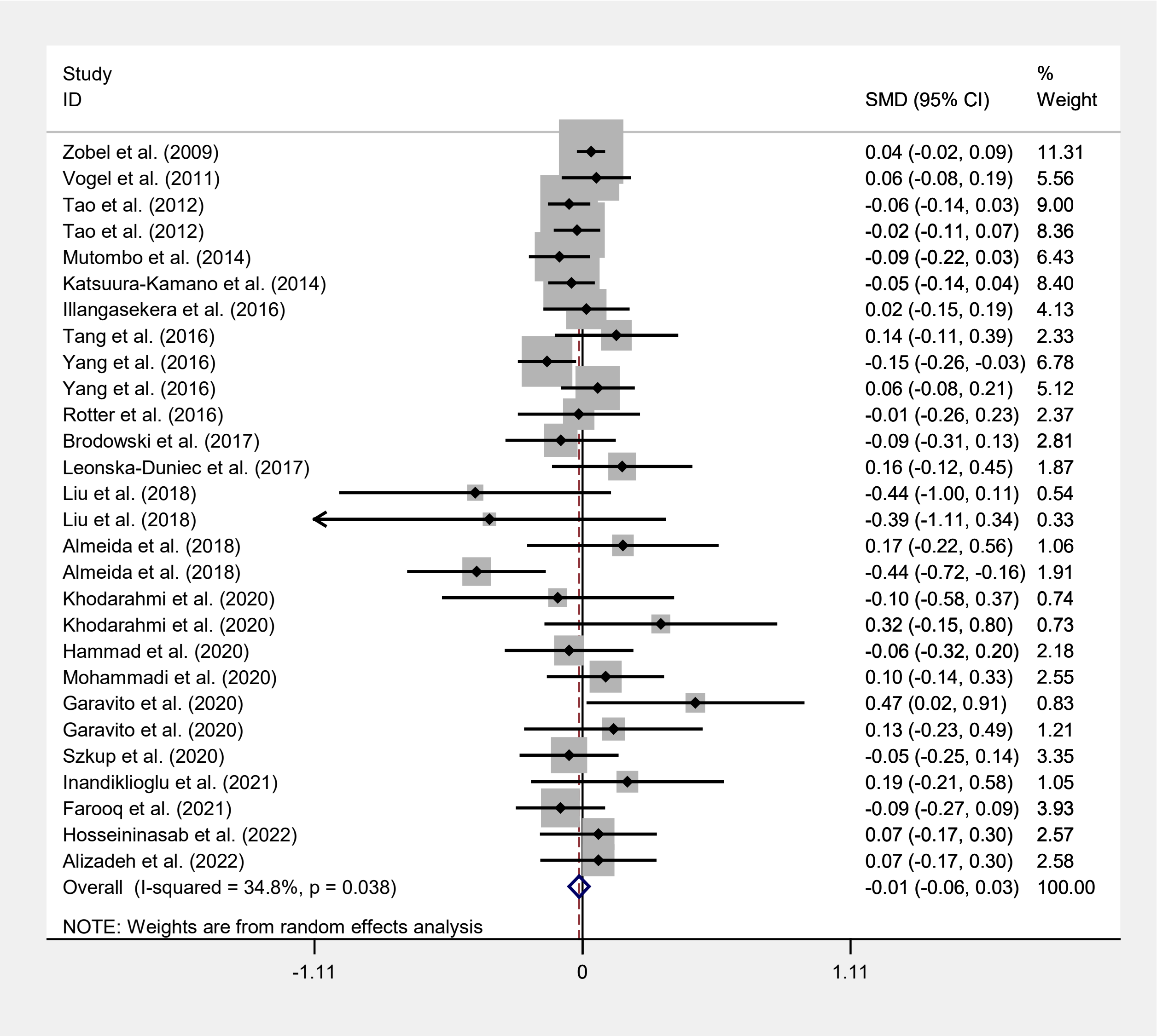

Figure S7. Forest plot of the association analysis between the MC4R rs17782313 polymorphism and blood levels of high-density lipoprotein cholesterol (HDL-C).

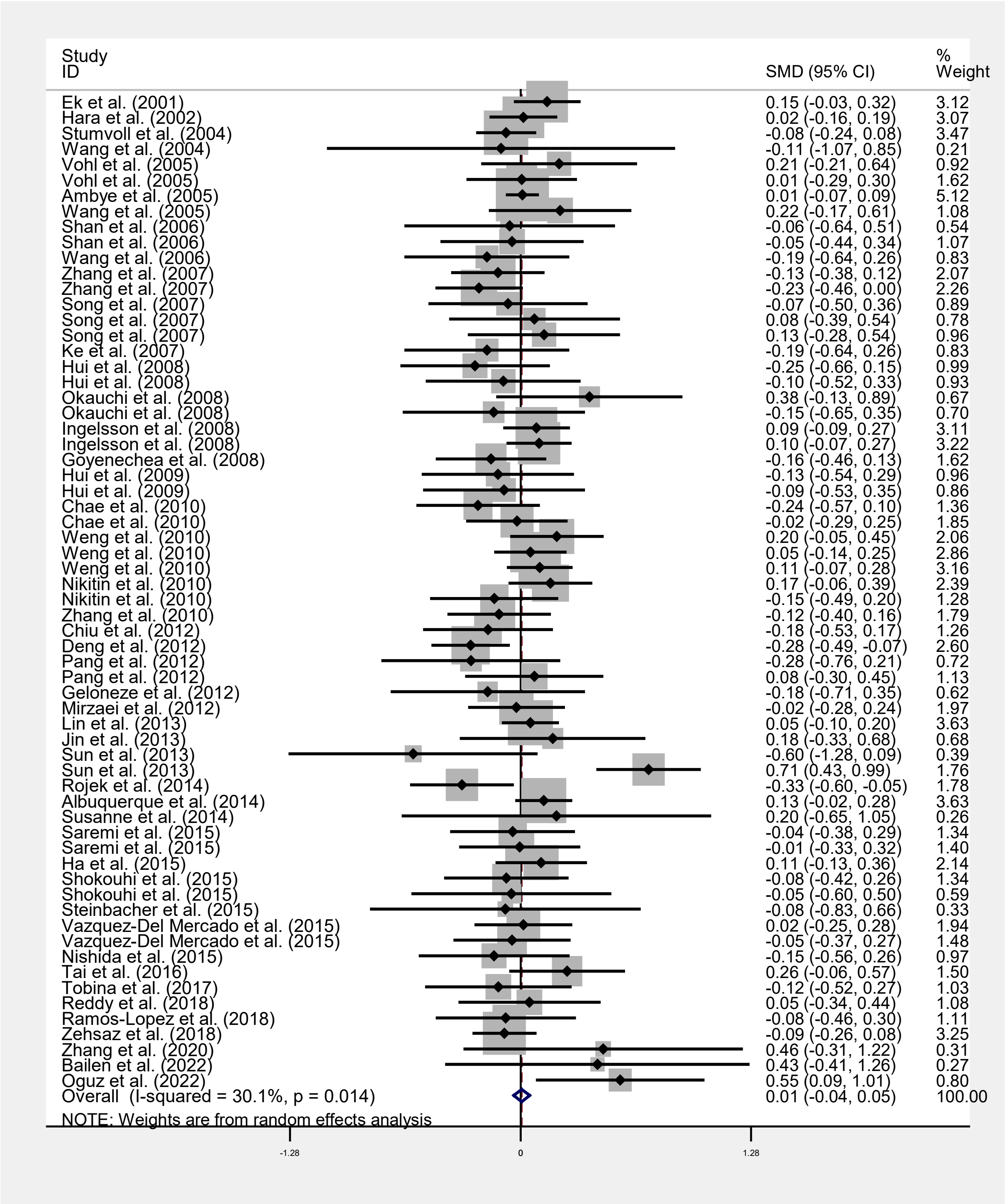

Figure S8. Forest plot of the association analysis between the PGC1α rs8192678 polymorphism and body mass index (BMI).

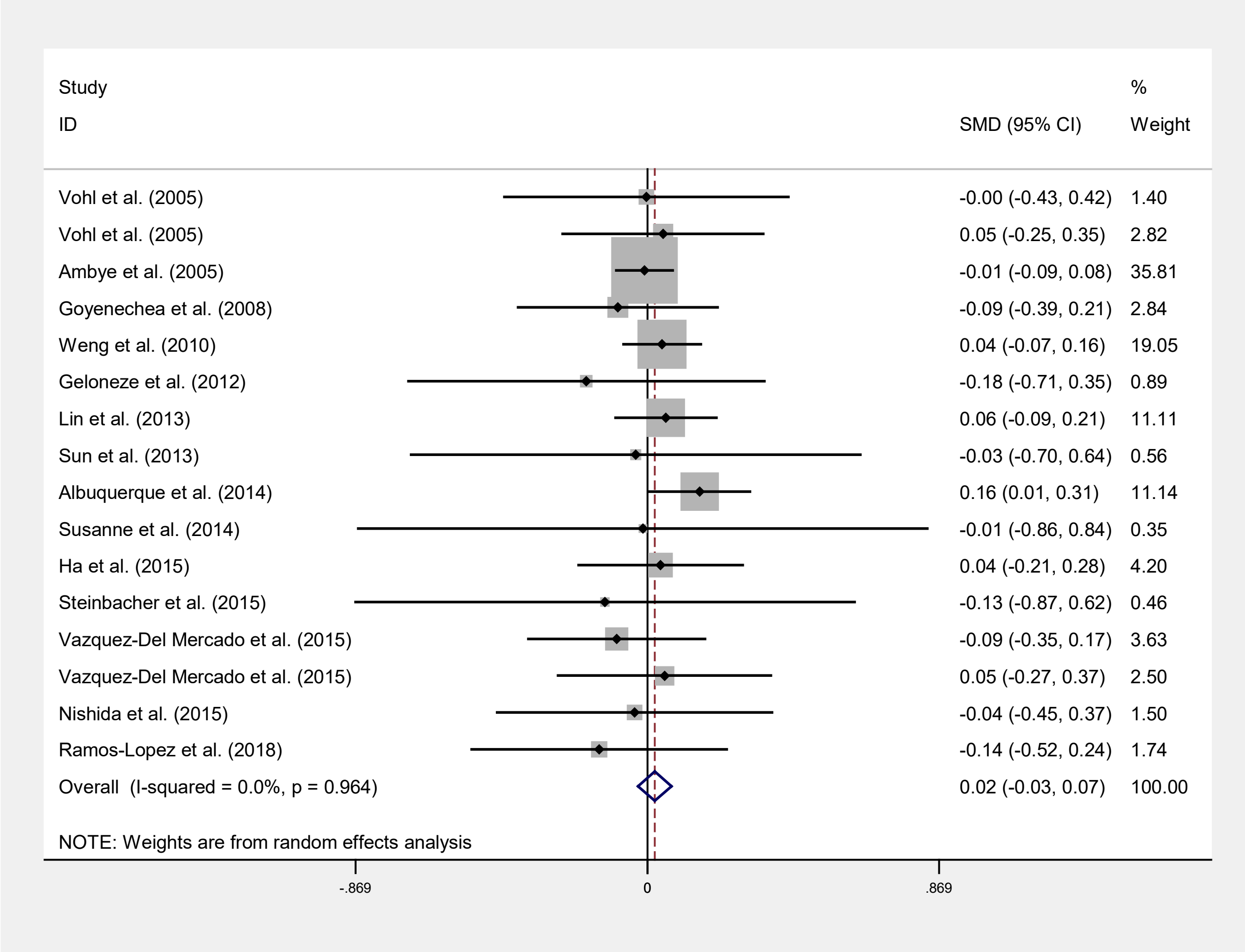

Figure S9. Forest plot of the association analysis between the PGC1α rs8192678 polymorphism and waist circumference (WC).

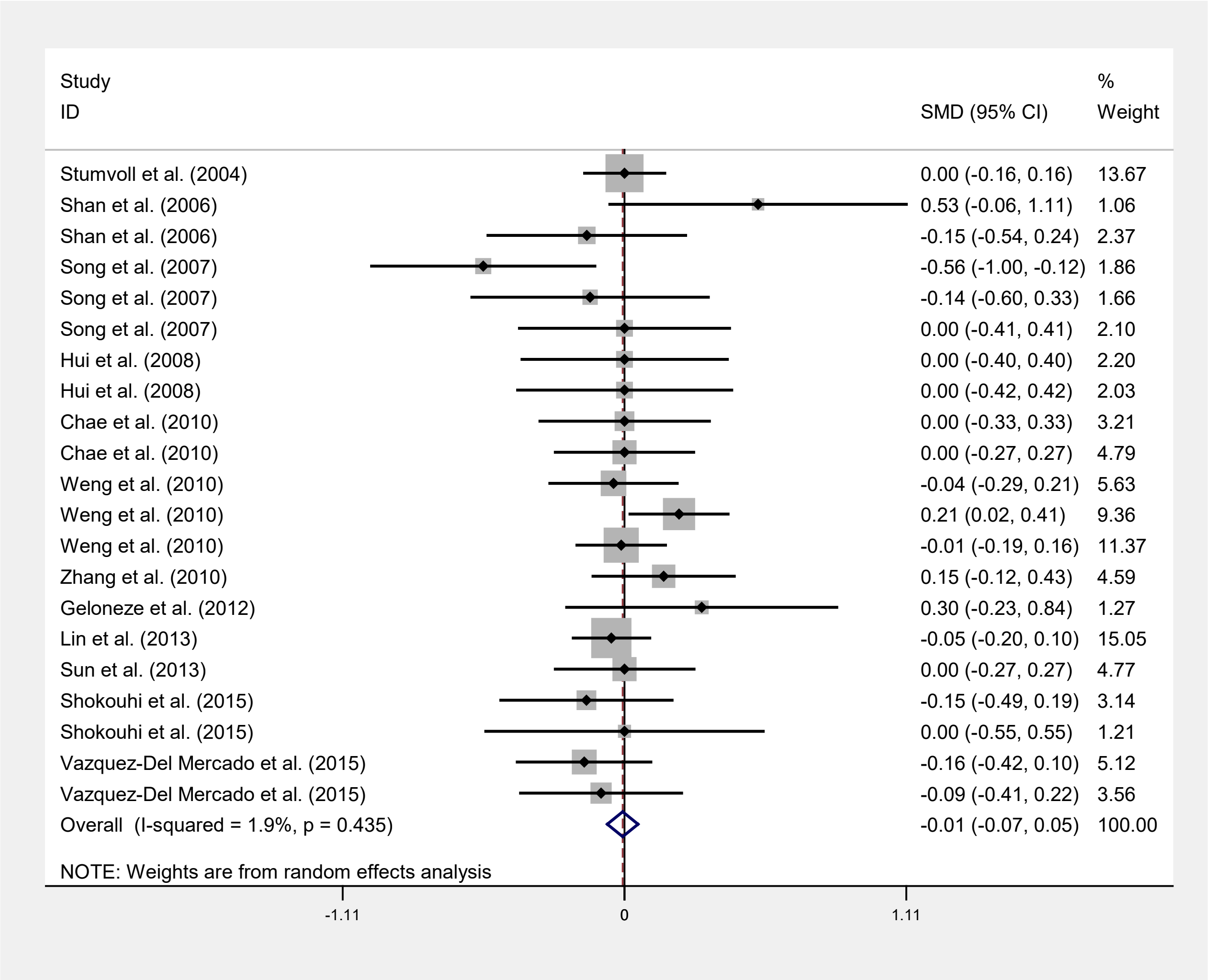

Figure S10. Forest plot of the association analysis between the PGC1α rs8192678 polymorphism and waist-to-hip ratio (WHR).

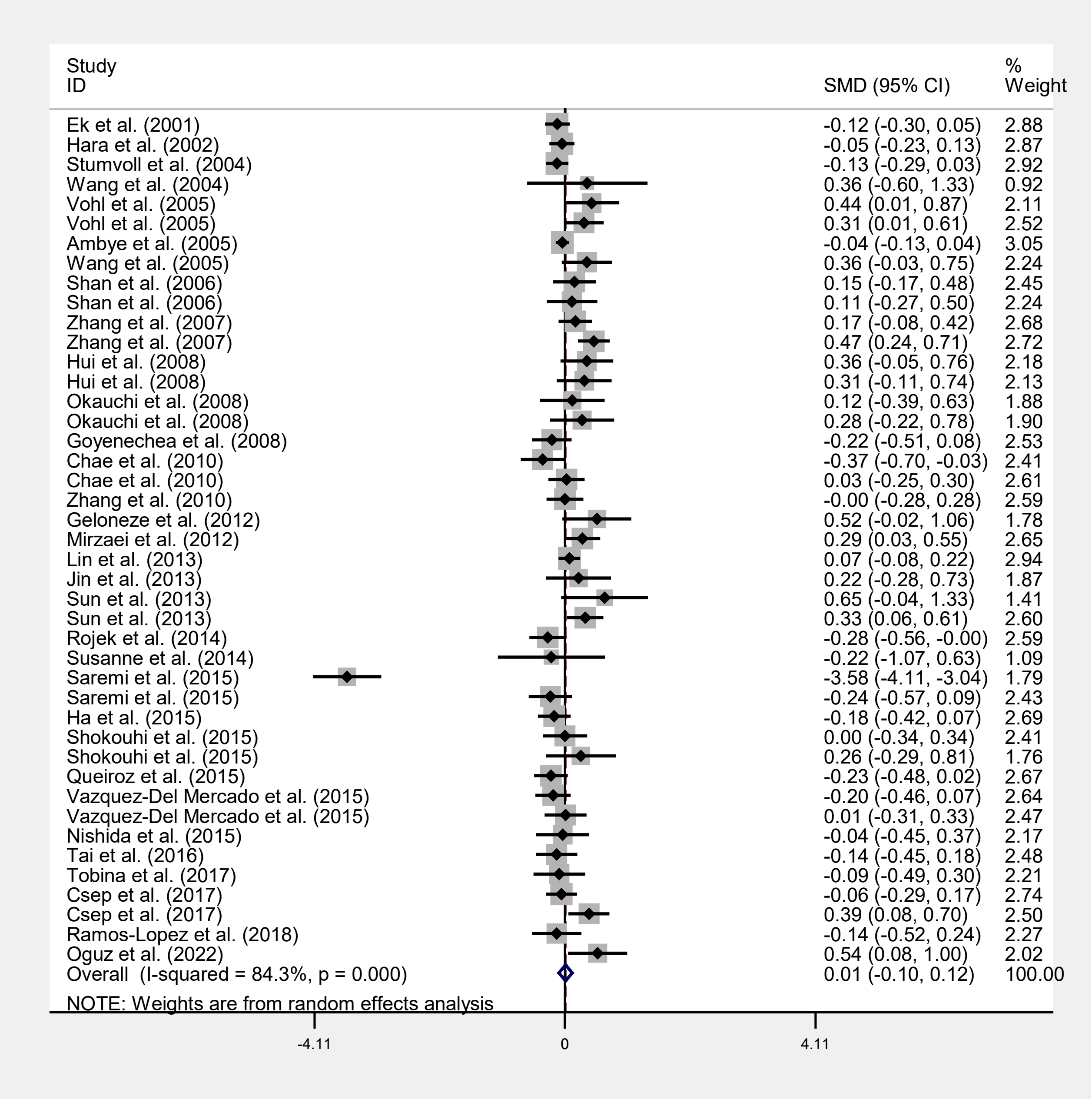

Figure S11. Forest plot of the association analysis between the PGC1α rs8192678 polymorphism and blood levels of glucose (GLU).

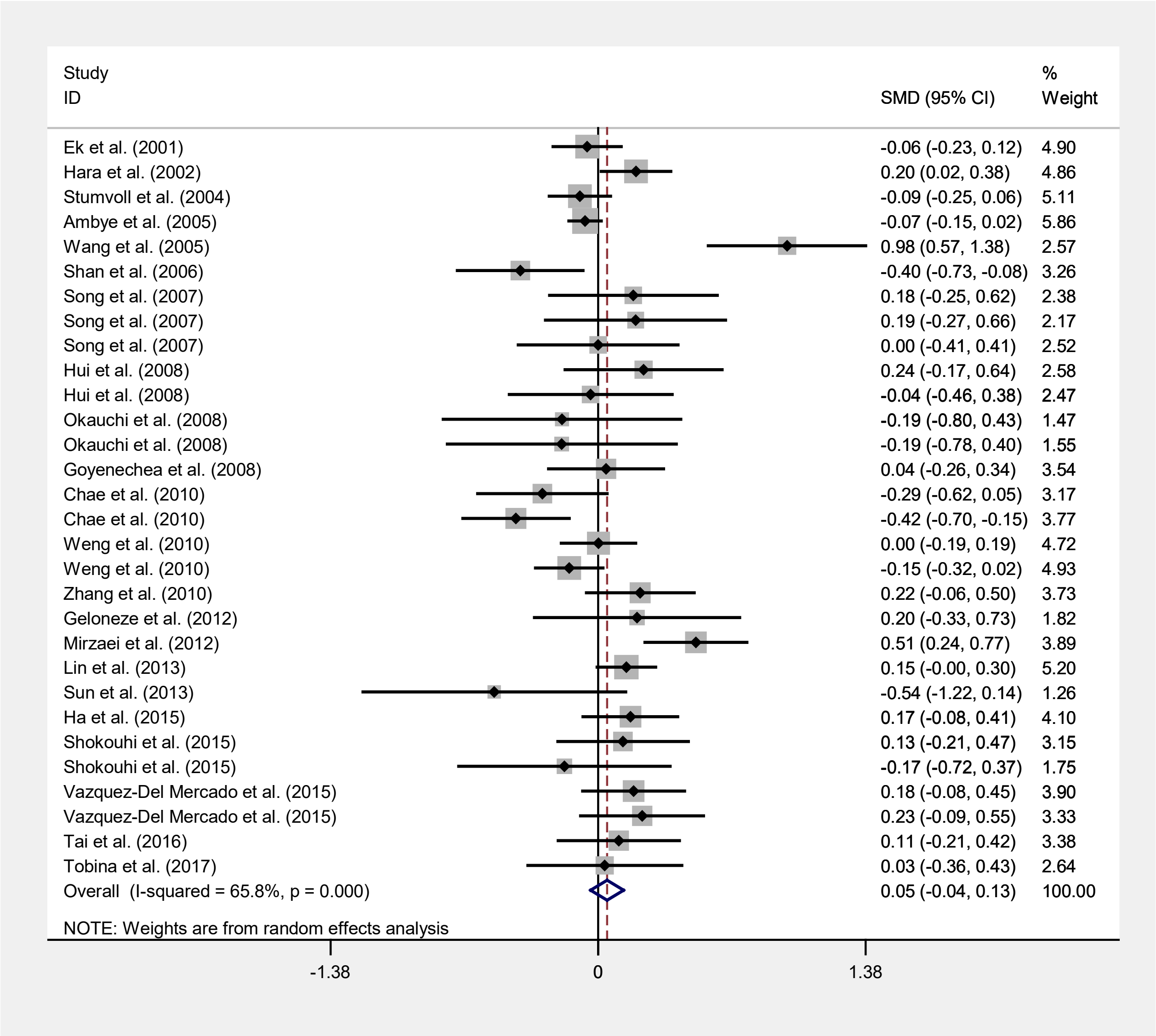

Figure S12. Forest plot of the association analysis between the PGC1α rs8192678 polymorphism and blood levels of insulin (INS).

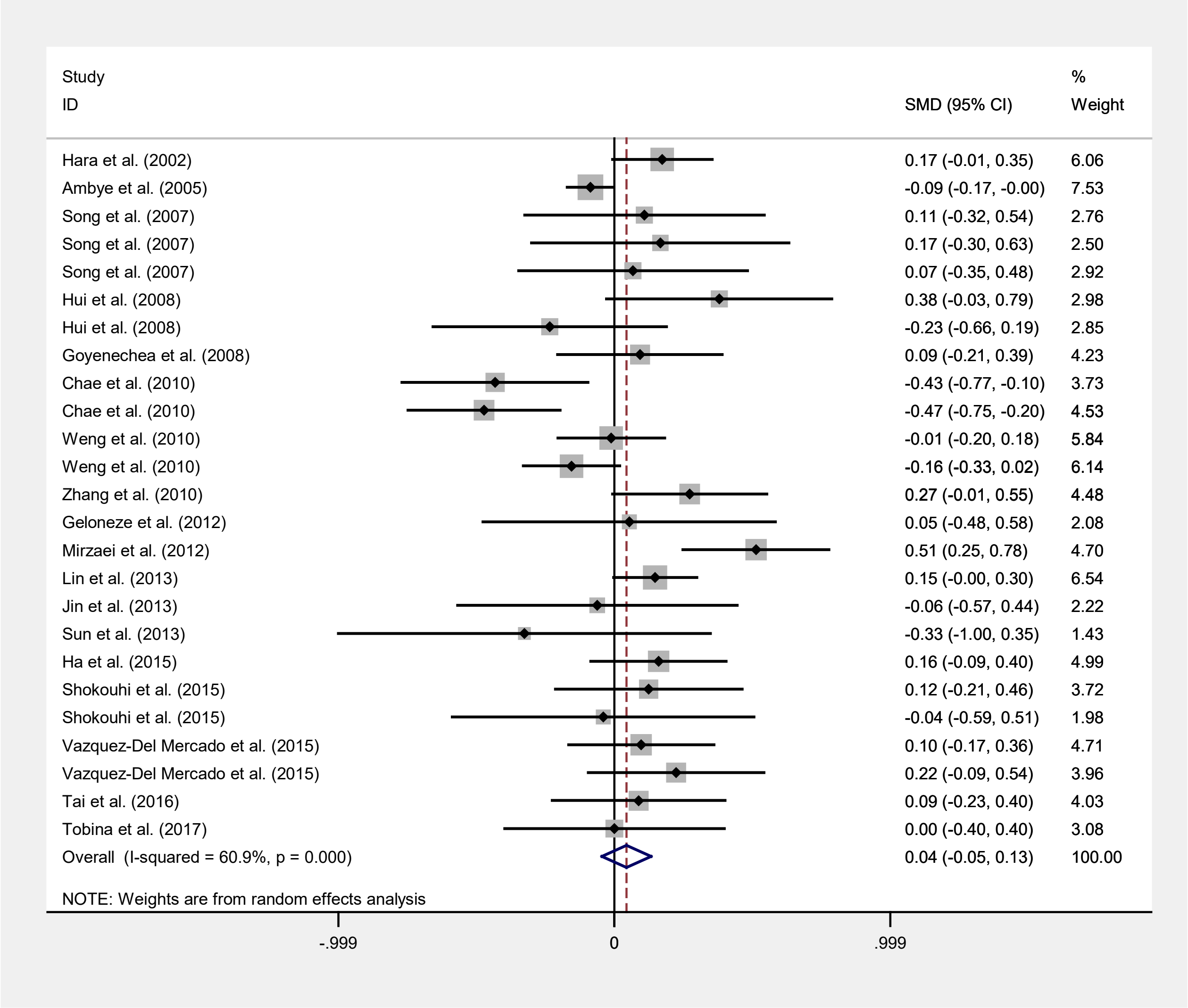

Figure S13. Forest plot of the association analysis between the PGC1α rs8192678 polymorphism and homeostasis model assessment of insulin resistance (HOMA-IR).

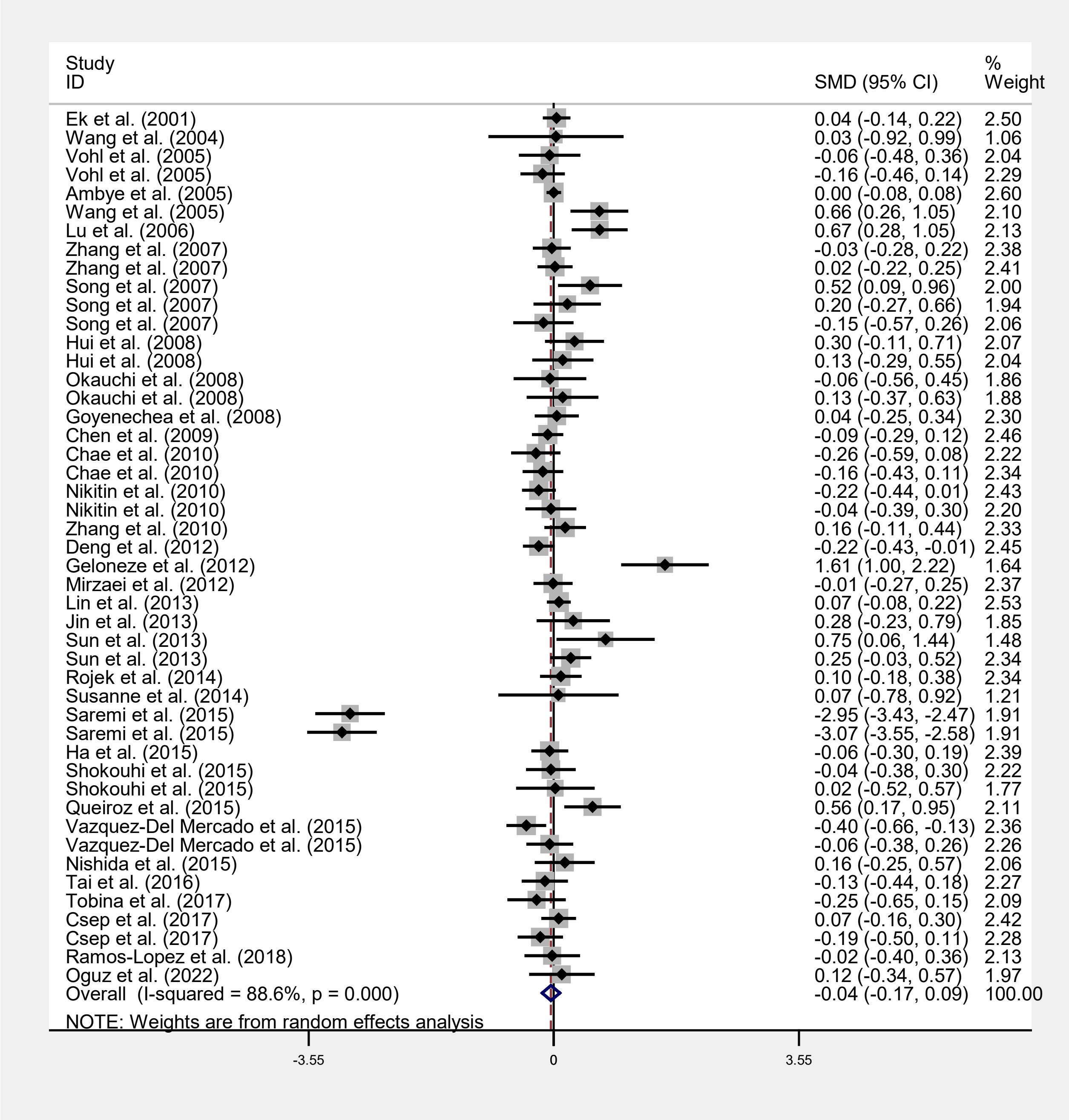

Figure S14. Forest plot of the association analysis between the PGC1α rs8192678 polymorphism and blood levels of triglycerides (TG).

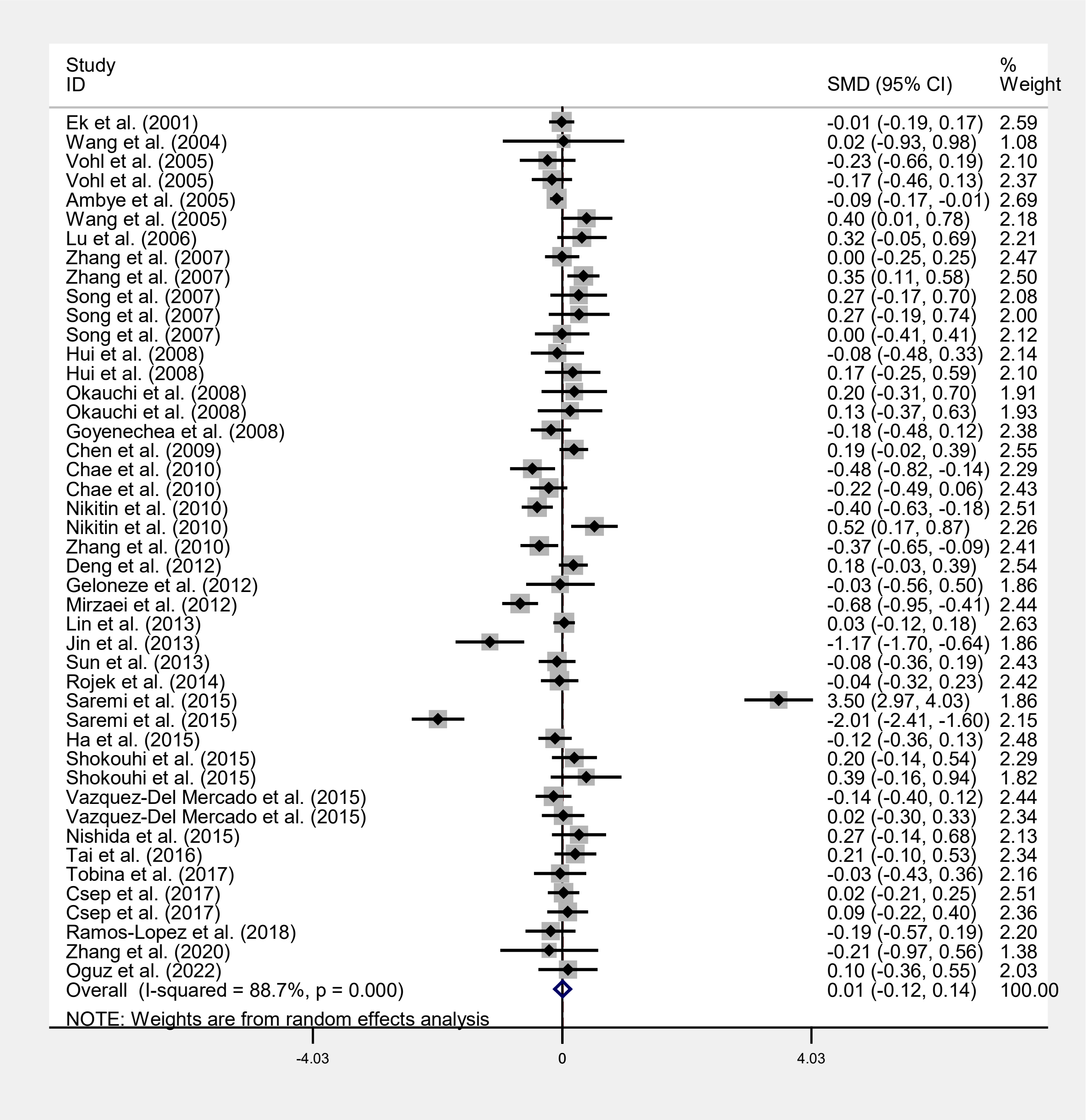

Figure S15. Forest plot of the association analysis between the PGC1α rs8192678 polymorphism and blood levels of total cholesterol (TC).

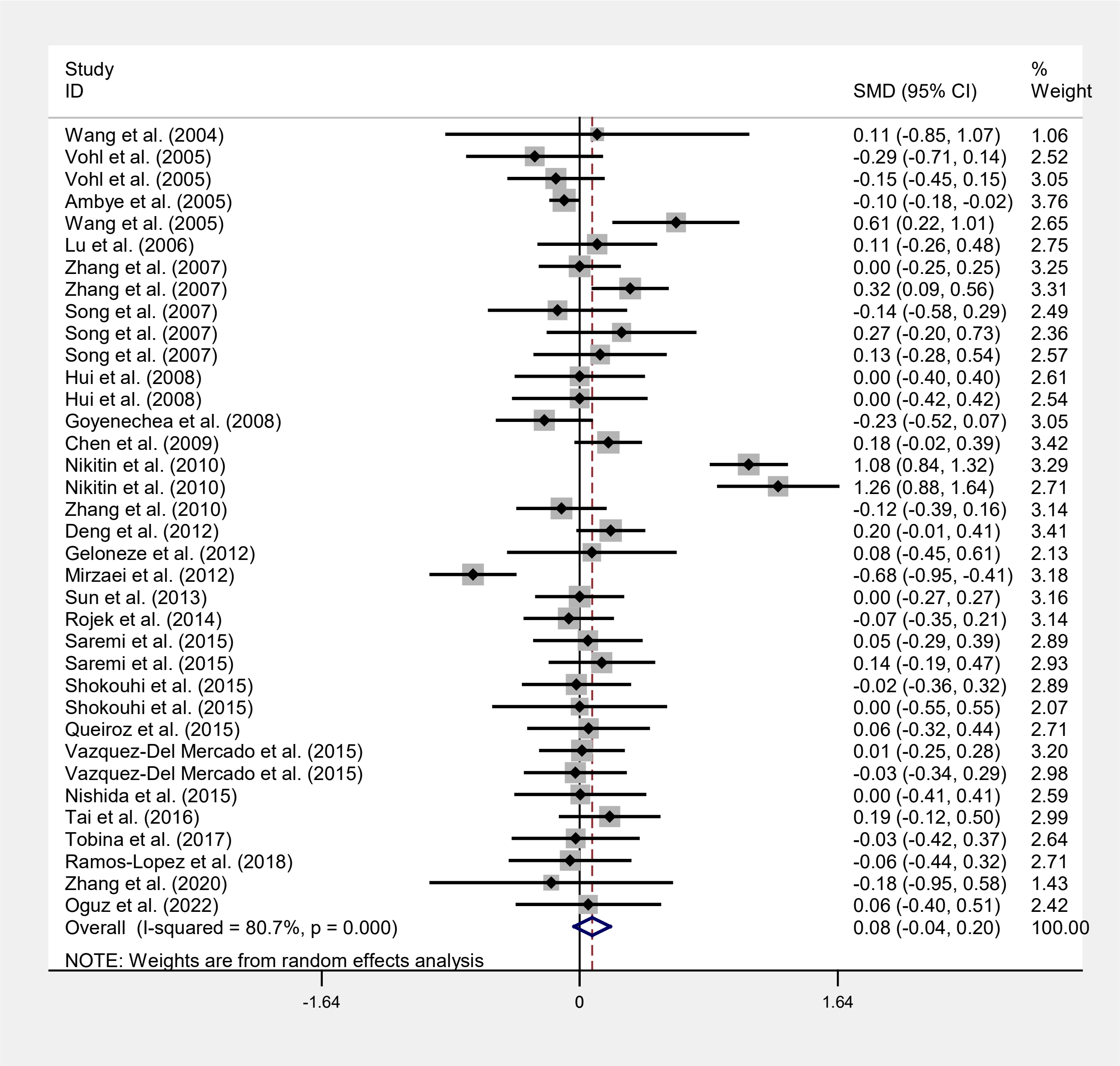

Figure S16. Forest plot of the association analysis between the PGC1α rs8192678 polymorphism and blood levels of low-density lipoprotein cholesterol (LDL-C).

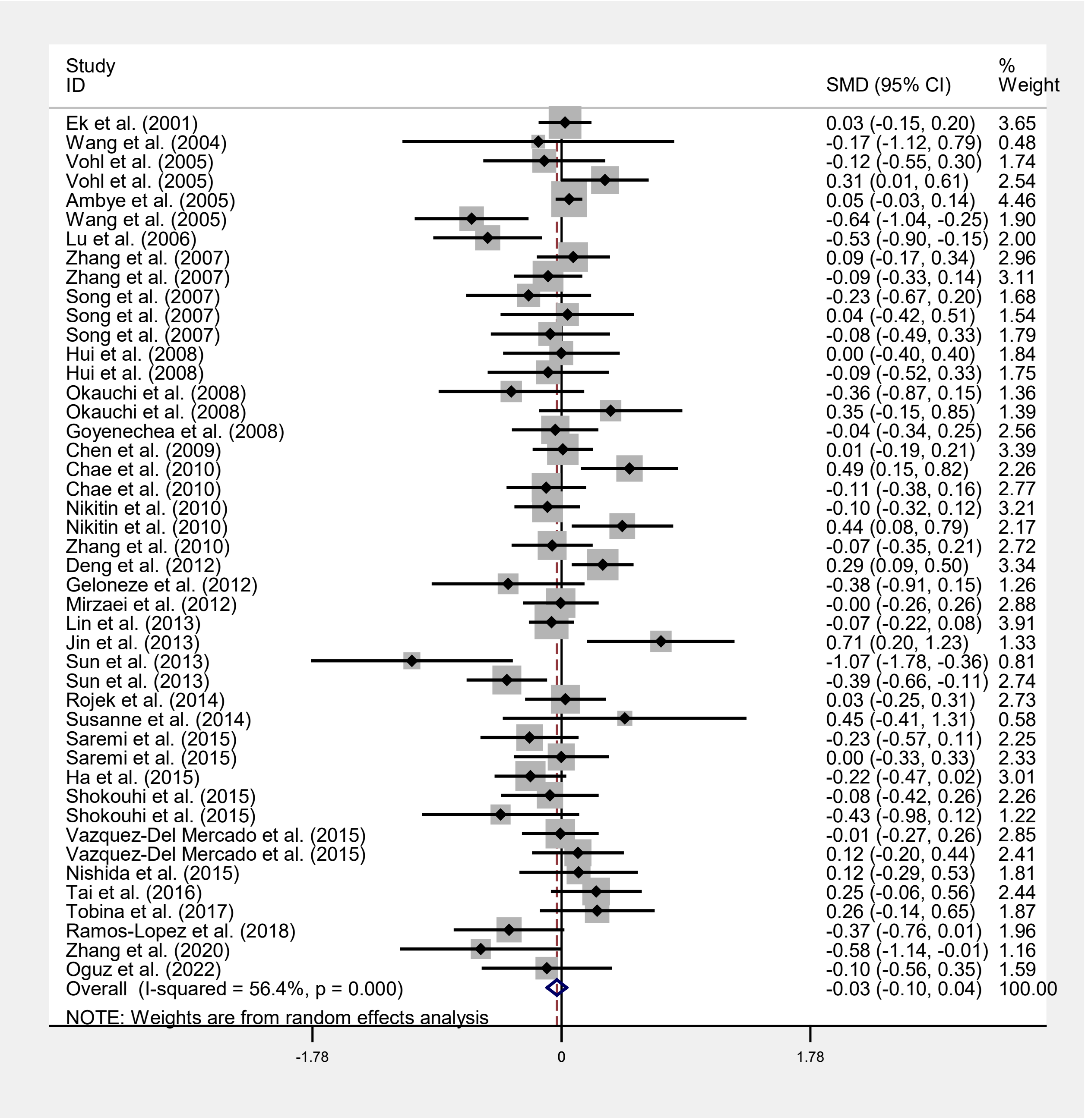

Figure S17. Forest plot of the association analysis between the PGC1α rs8192678 polymorphism and blood levels of high-density lipoprotein cholesterol (HDL-C).
