# Supplementary material for: The rs17782313 polymorphism near MC4R gene confers a high risk of obesity and hyperglycemia, while PGC1α rs8192678 polymorphism is weakly correlated with glucometabolic disorder: a systematic review and meta-analysis
Source: Front Endocrinol (Lausanne). 2023 Aug 9;14:1210455. doi: 10.3389/fendo.2023.1210455 (PMC10445758; doi:10.3389/fendo.2023.1210455)
Supplement: Supplementary file 2 [file Table_1.docx]

**Supplementary Tables S1-S9**

**Table S1.** Reference list of the studies included in the meta-analysis.

**Table S2.** Characteristics of the studies included in the meta-analysis for the MC4R rs17782313 polymorphism.

**Table S3.** Original data of the obesity indexes by genotypes of the MC4R rs17782313 polymorphism.

**Table S4.** Original data of the indexes of insulin resistance by genotypes of the MC4R rs17782313 polymorphism.

**Table S5.** Original data of blood lipids by genotypes of the MC4R rs17782313 polymorphism.

**Table S6.** Characteristics of the studies included in the meta-analysis for the PGC1α rs8192678 polymorphism.

**Table S7.** Original data of the obesity indexes by genotypes of the PGC1α rs8192678 polymorphism.

**Table S8.** Original data of the indexes of insulin resistance by genotypes of the PGC1α rs8192678 polymorphism.

**Table S9.** Original data of blood lipids by genotypes of the PGC1α rs8192678 polymorphism.

**Table S1. Reference list of the studies included in the meta-analysis.**

| **NO** | **Studies included in the meta-analysis for the MC4R rs17782313 polymorphism** |
| --- | --- |
| [1] | Haupt A, Thamer C, Heni M et al. Impact of variation near MC4R on whole-body fat distribution, liver fat, and weight loss. Obesity (Silver Spring). 2009; 17(10): 1942-5. doi: 10.1038/oby.2009.233. |
| [2] | Zobel DP, Andreasen CH, Grarup N et al. Variants near MC4R are associated with obesity and influence obesity-related quantitative traits in a population of middle-aged people: studies of 14,940 Danes. Diabetes. 2009; 58(3): 757-64. doi: 10.2337/db08-0620. |
| [3] | Tabara Y, Osawa H, Guo H et al. Prognostic significance of FTO genotype in the development of obesity in Japanese: the J-SHIPP study. Int J Obes (Lond). 2009; 33(11): 1243-8. doi: 10.1038/ijo.2009.161. |
| [4] | Petry CJ, Lopez-Bermejo A, Diaz M et al. Association between a common variant near MC4R and change in body mass index develops by two weeks of age. Horm Res Paediatr. 2010; 73(4): 275-80. doi: 10.1159/000284392. |
| [5] | Liu G, Zhu H, Lagou V et al. Common variants near melanocortin 4 receptor are associated with general and visceral adiposity in European- and African-American youth. J Pediatr. 2010; 156(4): 598-605. e1. doi: 10.1016/j.jpeds.2009.10.037. |
| [6] | Hasselbalch AL, Angquist L, Christiansen L et al. A variant in the fat mass and obesity-associated gene (FTO) and variants near the melanocortin-4 receptor gene (MC4R) do not influence dietary intake. J Nutr. 2010; 140(4): 831-4. doi: 10.3945/jn.109.114439. |
| [7] | Tschritter O, Haupt A, Preissl H et al. An Obesity Risk SNP (rs17782313) near the MC4R Gene Is Associated with Cerebrocortical Insulin Resistance in Humans. J Obes. 2011; 2011: 283153. doi: 10.1155/2011/283153. |
| [8] | Orkunoglu-Suer FE, Harmon BT, Gordish-Dressman H et al. MC4R variant is associated with BMI but not response to resistance training in young females. Obesity (Silver Spring). 2011; 19(3): 662-6. doi: 10.1038/oby.2010.180. |
| [9] | Vogel CI, Boes T, Reinehr T et al. Common variants near MC4R: exploring gender effects in overweight and obese children and adolescents participating in a lifestyle intervention. Obes Facts. 2011; 4(1): 67-75. doi: 10.1159/000324557. |
| [10] | Huang W, Sun Y, Sun J. Combined effects of FTO rs9939609 and MC4R rs17782313 on obesity and BMI in Chinese Han populations. Endocrine. 2011; 39(1): 69-74. doi: 10.1007/s12020-010-9413-6. |
| [11] | Tao L, Zhang Z, Chen Z et al. A Common variant near the melanocortin 4 receptor is associated with low-density lipoprotein cholesterol and total cholesterol in the Chinese Han population. Mol Biol Rep. 2012; 39(6): 6487-93. doi: 10.1007/s11033-012-1476-4. |
| [12] | Valette M, Poitou C, Le Beyec J et al. Melanocortin-4 receptor mutations and polymorphisms do not affect weight loss after bariatric surgery. PLoS One. 2012; 7(11): e48221. doi: 10.1371/journal.pone.0048221. |
| [13] | Corella D, Ortega-Azorin C, Sorli JV et al. Statistical and biological gene-lifestyle interactions of MC4R and FTO with diet and physical activity on obesity: new effects on alcohol consumption. PLoS One. 2012; 7(12): e52344. doi: 10.1371/journal.pone.0052344. |
| [14] | Marcadenti A, Fuchs FD, Matte U et al. Effects of FTO RS9939906 and MC4R RS17782313 on obesity, type 2 diabetes mellitus and blood pressure in patients with hypertension. Cardiovasc Diabetol. 2013; 12: 103. doi: 10.1186/1475-2840-12-103. |
| [15] | Czerwensky F, Leucht S, Steimer W. Association of the common MC4R rs17782313 polymorphism with antipsychotic-related weight gain. J Clin Psychopharmacol. 2013; 33(1): 74-9. doi: 10.1097/JCP.0b013e31827772db. |
| [16] | Jaaskelainen A, Schwab U, Kolehmainen M et al. Meal frequencies modify the effect of common genetic variants on body mass index in adolescents of the northern Finland birth cohort 1986. PLoS One. 2013; 8(9): e73802. doi: 10.1371/journal.pone.0073802. |
| [17] | Sull JW, Lee M, Jee SH. Replication of genetic effects of MC4R polymorphisms on body mass index in a Korean population. Endocrine. 2013; 44(3): 675-9. doi: 10.1007/s12020-013-9909-y. |
| [18] | Mutombo PB, Yamasaki M, Hamano T et al. MC4R rs17782313 gene polymorphism was associated with glycated hemoglobin independently of its effect on BMI in Japanese: the Shimane COHRE study. Endocr Res. 2014; 39(3): 115-9. doi: 10.3109/07435800.2013.844163. |
| [19] | Katsuura-Kamano S, Uemura H, Arisawa K et al. A polymorphism near MC4R gene (rs17782313) is associated with serum triglyceride levels in the general Japanese population: the J-MICC Study. Endocrine. 2014; 47(1): 81-9. doi: 10.1007/s12020-014-0306-y. |
| [20] | Arrizabalaga M, Larrarte E, Margareto J et al. Preliminary findings on the influence of FTO rs9939609 and MC4R rs17782313 polymorphisms on resting energy expenditure, leptin and thyrotropin levels in obese non-morbid premenopausal women. J Physiol Biochem. 2014; 70(1): 255-62. doi: 10.1007/s13105-013-0300-5. |
| [21] | Acosta A, Camilleri M, Shin A et al. Association of melanocortin 4 receptor gene variation with satiation and gastric emptying in overweight and obese adults. Genes Nutr. 2014; 9(2): 384. doi: 10.1007/s12263-014-0384-8. |
| [22] | Yang J, Gao Q, Gao X et al. Melanocortin-4 receptor rs17782313 polymorphisms are associated with serum triglycerides in older Chinese women. Asia Pac J Clin Nutr. 2016; 25(1): 213-9. doi: 10.6133/apjcn.2016.25.1.18. |
| [23] | Martins MC, Trujillo J, Farias DR et al. Association of the FTO (rs9939609) and MC4R (rs17782313) gene polymorphisms with maternal body weight during pregnancy. Nutrition. 2016; 32(11-12): 1223-30. doi: 10.1016/j.nut.2016.04.009. |
| [24] | Rotter I, Skonieczna-Żydecka K, Kosik-Bogacka D et al. Relationships between FTO rs9939609, MC4R rs17782313, and PPARγ rs1801282 polymorphisms and the occurrence of selected metabolic and hormonal disorders in middle-aged and elderly men - a preliminary study. Clin Interv Aging. 2016; 11: 1723-1732. doi: 10.2147/CIA.S120253. |
| [25] | Vega JA, Salazar G, Hodgson MI et al. Melanocortin-4 Receptor Gene Variation Is Associated with Eating Behavior in Chilean Adults. Ann Nutr Metab. 2016; 68(1): 35-41. doi: 10.1159/000439092. |
| [26] | Illangasekera YA, Kumarasiri RP, Fernando DJ et al. Association of FTO and near MC4R variants with obesity measures in urban and rural dwelling Sri Lankans. Obes Res Clin Pract. 2016; 10 Suppl 1: S117-S124. doi: 10.1016/j.orcp.2016.02.003. |
| [27] | Tang N, Chen YD, Zeng T et al. Relationship between SNP rs17782313 variation in MC4R gene and obesity in zhuang nationality children. Journal of Molecular Diagnosis and Therapy, 2016, 8(02): 94-98. |
| [28] | Brodowski J, Szkup M, Jurczak A et al.Searching for the relationship between the parameters of metabolic syndrome and the rs17782313 (T>C) polymorphism of the MC4R gene in postmenopausal women. Clin Interv Aging. 2017; 12: 549-555. doi: 10.2147/CIA.S129874. |
| [29] | Leonska-Duniec A, Jastrzębski Z, Zarębska A et al. Impact of the Polymorphism Near MC4R (rs17782313) on Obesity- and Metabolic-Related Traits in Women Participating in an Aerobic Training Program. J Hum Kinet. 2017; 58: 111-119. doi: 10.1515/hukin-2017-0073. |
| [30] | Song JY, Song QY, Wang S et al. Physical Activity and Sedentary Behaviors Modify the Association between Melanocortin 4 Receptor Gene Variant and Obesity in Chinese Children and Adolescents. PLoS One. 2017; 12(1): e0170062. doi: 10.1371/journal.pone.0170062. |
| [31] | Rana S, Rahmani S, Mirza S. MC4R variant rs17782313 and manifestation of obese phenotype in Pakistani females. RSC Adv. 2018; 8(30): 16957-16972. doi: 10.1039/c8ra00695d. |
| [32] | Almeida SM, Furtado JM, Mascarenhas P et al. Association between LEPR, FTO, MC4R, and PPARG-2 polymorphisms with obesity traits and metabolic phenotypes in school-aged children. Endocrine. 2018; 60(3): 466-478. doi: 10.1007/s12020-018-1587-3. |
| [33] | Liu BY, Wang Y, Liu C et al. Association of melanocortin-4 receptor gene rs17782313 polymorphism with weight loss after bariatric surgery in obese patients. Journal of Chongqing Medical University, 2018, 43(12): 1643-1646. |
| [34] | Batarfi AA, Filimban N, Bajouh OS et al. MC4R variants rs12970134 and rs17782313 are associated with obese polycystic ovary syndrome patients in the Western region of Saudi Arabia. BMC Med Genet. 2019; 20(1): 144. doi: 10.1186/s12881-019-0876-x. |
| [35] | Adamska-Patruno E, Goscik J, Czajkowski P et al. The MC4R genetic variants are associated with lower visceral fat accumulation and higher postprandial relative increase in carbohydrate utilization in humans. Eur J Nutr. 2019; 58(7): 2929-2941. doi: 10.1007/s00394-019-01955-0. |
| [36] | Zhang Y, Ren H, Wang Q et al. Chinese Antipsychotics Pharmacogenomics Consortium. Testing the role of genetic variation of the MC4R gene in Chinese population in antipsychotic-induced metabolic disturbance. Sci China Life Sci. 2019; 62(4): 535-543. doi: 10.1007/s11427-018-9489-x. |
| [37] | Sull JW, Kim G, Jee SH. Association of MC4R (rs17782313) with diabetes and cardiovascular disease in Korean men and women. BMC Med Genet. 2020; 21(1): 160. doi: 10.1186/s12881-020-01100-3. |
| [38] | Hammad MM, Abu-Farha M, Hebbar P et al. MC4R Variant rs17782313 Associates With Increased Levels of DNAJC27, Ghrelin, and Visfatin and Correlates With Obesity and Hypertension in a Kuwaiti Cohort. Front Endocrinol (Lausanne). 2020; 11: 437. doi: 10.3389/fendo.2020.00437. |
| [39] | Mohammadi M, Khodarahmi M, Kahroba H et al. Dietary patterns interact with the variations of 18q21. 23 rs17782313 locus on regulation of hypothalamic-pituitary axis hormones and cardio-metabolic risk factors in obesity. Eat Weight Disord. 2020; 25(5): 1447-1459. doi: 10.1007/s40519-020-00855-1. |
| [40] | Khodarahmi M, Kahroba H, Jafarabadi MA et al. Dietary quality indices modifies the effects of melanocortin-4 receptor (MC4R) rs17782313 polymorphism on cardio-metabolic risk factors and hypothalamic hormones in obese adults. BMC Cardiovasc Disord. 2020; 20(1): 57. doi: 10.1186/s12872-020-01366-8. |
| [41] | Garavito P, Mosquera-Heredia MI, Fang L et al. Polymorphisms of leptin-melanocortin system genes associated with obesity in an adult population from Barranquilla. Biomedica. 2020; 40(2): 257-269. English, Spanish. doi: 10.7705/biomedica.4827. |
| [42] | Szkup M, Brodowski J, Jurczak A et al. Seeking genetic determinants of selected metabolic disorders in women aged 45-60. Ann Agric Environ Med. 2020; 27(3): 407-412. doi: 10.26444/aaem/112579. |
| [43] | Rebelos E, Honka MJ, Ekblad L et al. The Obesity Risk SNP (rs17782313) near the MC4R Gene Is Not Associated with Brain Glucose Uptake during Insulin Clamp-A Study in Finns. J Clin Med. 2021; 10(6): 1312. doi: 10.3390/jcm10061312. |
| [44] | Farooq S, Rana S, Siddiqui AJ et al. Association of metabolites with obesity based on two gene variants, MC4R rs17782313 and BDNF rs6265. Biochim Biophys Acta Mol Basis Dis. 2021; 1867(7): 166144. doi: 10.1016/j.bbadis.2021.166144. |
| [45] | Raskiliene A, Smalinskiene A, Kriaucioniene V et al. Associations of MC4R, LEP, and LEPR Polymorphisms with Obesity-Related Parameters in Childhood and Adulthood. Genes (Basel). 2021; 12(6): 949. doi: 10.3390/genes12060949. |
| [46] | Inandiklioglu N, Yaşar A. Association between rs1421085 and rs9939609 Polymorphisms of Fat Mass and Obesity-Associated Gene with High-Density Lipoprotein Cholesterol and Triglyceride in Obese Turkish Children and Adolescents. J Pediatr Genet. 2021; 10(1): 9-15. doi: 10.1055/s-0040-1713154. |
| [47] | Adamska-Patruno E, Bauer W, Bielska D et al. An Association between Diet and MC4R Genetic Polymorphism, in Relation to Obesity and Metabolic Parameters-A Cross Sectional Population-Based Study. Int J Mol Sci. 2021; 22(21): 12044. doi: 10.3390/ijms222112044. |
| [48] | Rahati S, Qorbani M, Naghavi A et al. Association and interaction of the MC4R rs17782313 polymorphism with plasma ghrelin, GLP-1, cortisol, food intake and eating behaviors in overweight/obese Iranian adults. BMC Endocr Disord. 2022; 22(1): 234. doi: 10.1186/s12902-022-01129-w. |
| [49] | Alizadeh S, Pooyan S, Mirzababaei A et al. Interaction of MC4R rs17782313 variants and dietary carbohydrate quantity and quality on basal metabolic rate and general and central obesity in overweight/obese women: a cross-sectional study. BMC Endocr Disord. 2022; 22(1): 121. doi: 10.1186/s12902-022-01023-5. |
| [50] | Hosseininasab D, Mirzababaei A, Abaj F et al. Are there any interactions between modified Nordic-style diet score and MC4R polymorphism on cardiovascular risk factors among overweight and obese women? A cross-sectional study. BMC Endocr Disord. 2022; 22(1): 221. doi: 10.1186/s12902-022-01132-1. |
| **NO** | **Studies included in the meta-analysis for the PGC1α rs8192678 polymorphism** |
| [1] | Ek J, Andersen G, Urhammer SA et al. Mutation analysis of peroxisome proliferator-activated receptor-gamma coactivator-1 (PGC-1) and relationships of identified amino acid polymorphisms to Type II diabetes mellitus. Diabetologia. 2001; 44(12): 2220-6. doi: 10.1007/s001250100032. |
| [2] | Hara K, Tobe K, Okada T et al. A genetic variation in the PGC-1 gene could confer insulin resistance and susceptibility to Type II diabetes. Diabetologia. 2002; 45(5): 740-3. doi: 10.1007/s00125-002-0803-z. |
| [3] | Stumvoll M, Fritsche A, tHart LM et al. The Gly482Ser variant in the peroxisome proliferator-activated receptor gamma coactivator-1 is not associated with diabetes-related traits in non-diabetic German and Dutch populations. Exp Clin Endocrinol Diabetes. 2004; 112(5): 253-7. doi: 10.1055/s-2004-817972. |
| [4] | Wang XH, Yan KJ, Zhang ZB et al.The association of peroxisome proliferator-activated receptor-γ coactivator-1 gene Gly482Ser polymorphism with type 2 diabetes mellitus. Journal of Medical Science Yanbian University, 2004; 27(2): 83-89. |
| [5] | Ambye L, Rasmussen S, Fenger M et al. Studies of the Gly482Ser polymorphism of the peroxisome proliferator-activated receptor gamma coactivator 1alpha (PGC-1alpha) gene in Danish subjects with the metabolic syndrome. Diabetes Res Clin Pract. 2005; 67(2): 175-9. doi: 10.1016/j.diabres.2004.06.013. |
| [6] | Vohl MC, Houde A, Lebel S et al. Effects of the peroxisome proliferator-activated receptor-gamma co-activator-1 Gly482Ser variant on features of the metabolic syndrome. Mol Genet Metab. 2005; 86(1-2): 300-6. doi: 10.1016/j.ymgme.2005.07.002. |
| [7] | Wang YB, Yu YC, Li Z et al. Study on the relationship between polymorphisms of peroxisome proliferators-activated receptor-gamma coactivator-1alpha gene and type 2 diabetes in Shanghai Hans in China. Chin J Med Genet. 2005; 22(4): 453-6. |
| [8] | Wang Y, Wu X, Cao Y et al. Polymorphisms of the peroxisome proliferator-activated receptor-gamma and its coactivator-1alpha genes in Chinese women with polycystic ovary syndrome. Fertil Steril. 2006; 85(5): 1536-40. doi: 10.1016/j.fertnstert.2005.10.047. |
| [9] | Lu WS, Cheng H, Huang Q et al. Screening the PGC-1α gene for SNPs and analyzing PGC-1α Gly482Ser mutation in Chinese population. Chin J Diabetes, 2006; 14(3): 192-194. |
| [10] | Shan L, Wang C, Ji LN et al. Association of PGC-1α gene Gly482Ser polymorphism with type 2 diabetes mellitus. Chin J Diabetes, 2006; 14(2): 103-104. |
| [11] | Zhang SL, Lu WS, Yan L et al. Association between peroxisome proliferator-activated receptor-gamma coactivator-1alpha gene polymorphisms and type 2 diabetes in southern Chinese population: role of altered interaction with myocyte enhancer factor 2C. Chin Med J (Engl). 2007 ; 120(21): 1878-85. |
| [12] | Song J, Jia WP, Fang QC et al. Relationg between peroxisome proliferator-activated receptor-γ coactivator-1α Gly482Ser variant and glucose and lipid metabolism in Chinese subjects and patients. Shangha iMed J, 2007; 30(7): 485-489. |
| [13] | Ke L, Wang Y, Wu XK et al. Polymorphisms of the peroxisome proliferator-activated receptor-γand its coactivator-1 genes in Chinese women with polycystic ovary syndrome.Journal of Reproductive Medicine, 2007; 16(4) : 236-241. |
| [14] | Hui Y, Yu-Yuan L, Yu-Qiang N et al. Effect of peroxisome proliferator-activated receptors-gamma and co-activator-1alpha genetic polymorphisms on plasma adiponectin levels and susceptibility of non-alcoholic fatty liver disease in Chinese people. Liver Int. 2008; 28(3): 385-92. doi: 10.1111/j.1478-3231.2007.01623.x. |
| [15] | Okauchi Y, Iwahashi H, Okita K et al. PGC-1alpha Gly482Ser polymorphism is associated with the plasma adiponectin level in type 2 diabetic men. Endocr J. 2008; 55(6): 991-7. doi: 10.1507/endocrj.k08e-070. |
| [16] | Goyenechea E, Crujeiras AB, Abete I et al. Enhanced short-term improvement of insulin response to a low-caloric diet in obese carriers the Gly482Ser variant of the PGC-1alpha gene. Diabetes Res Clin Pract. 2008; 82(2): 190-6. doi: 10.1016/j.diabres.2008.08.011. |
| [17] | Ingelsson E, Bennet L, Ridderstrale M et al. The PPARGC1A Gly482Ser polymorphism is associated with left ventricular diastolic dysfunction in men. BMC Cardiovasc Disord. 2008; 8: 37. doi: 10.1186/1471-2261-8-37. |
| [18] | Hui YC, Guan YF, LI Z et al.Association of Peroxisome proliferator activated receptor-γcoactivator- 1α(PGC- 1α)Gly482Ser polymorphism with type 2 diabetes mellitusin Chinese women.Journal of Da lian Medical University, 2009; 31(3): 295-298. |
| [19] | Chen NF, Yan WL, Huang JF et al. PGC-1α gene polymorphisms are Associated with the Lipid Levels in Northern Han Chinese population.Molecular Gardiology of China, 2009; 9(2): 88-93. |
| [20] | Weng SW, Lin TK, Wang PW et al. Gly482Ser polymorphism in the peroxisome proliferator-activated receptor gamma coactivator-  -1alpha gene is associated with oxidative stress and abdominal obesity. Metabolism. 2010; 59(4): 581-6. doi: 10.1016/j.metabol.2009.08.021. |
| [21] | Chae SJ, Kim JJ, Choi YM et al. Peroxisome proliferator-activated receptor-gamma and its coactivator-1alpha gene polymorphisms in Korean women with polycystic ovary syndrome. Gynecol Obstet Invest. 2010; 70(1): 1-7. doi: 10.1159/000279309. |
| [22] | Zhang KH, Huang Q, Dai XP et al. Effects of the peroxisome proliferator activated receptor-γ coactivator-1α (PGC-1α) Thr394Thr and Gly482Ser polymorphisms on rosiglitazone response in Chinese patients with type 2 diabetes mellitus. J Clin Pharmacol. 2010; 50(9): 1022-30. doi: 10.1177/0091270009355159. |
| [23] | Nikitin AG, Chistiakov DA, Minushkina LO et al. Association of the CYBA, PPARGC1A, PPARG3, and PPARD gene variants with coronary artery disease and metabolic risk factors of coronary atherosclerosis in a Russian population. Heart Vessels. 2010; 25(3): 229-36. doi: 10.1007/s00380-009-1159-9. |
| [24] | Chiu LL, Chen TW, Hsieh SS et al. ACE I/D, ACTN3 R577X, PPARD T294C and PPARGC1A Gly482Ser polymorphisms and physical fitness in Taiwanese late adolescent girls. J Physiol Sci. 2012; 62(2): 115-21. doi: 10.1007/s12576-011-0189-0. |
| [25] | Geloneze SR, Geloneze B, Morari J et al. PGC1α gene Gly482Ser polymorphism predicts improved metabolic, inflammatory and vascular outcomes following bariatric surgery. Int J Obes (Lond). 2012; 36(3): 363-8. doi: 10.1038/ijo.2011.176. |
| [26] | Mirzaei K, Hossein-nezhad A, Emamgholipour S et al. An exonic peroxisome proliferator-activated receptor-γ coactivator-1α variation may mediate the resting energy expenditure through a potential regulatory role on important gene expression in this pathway. J Nutrigenet Nutrigenomics. 2012; 5(2): 59-71. doi: 10.1159/000337352. |
| [27] | Deng DY, Yuan WL, Feng Q et al. Association of PGC- 1α gene polymorphism with impaired glucose regulation. Chin J Lab Diagn, 2012; 16(11): 2047-2052. |
| [28] | Pang J, Yan XD, Mo XY et al.Gly482Ser variability analysis of peroxisome proliferator-activated receptor-γ coactivator-1α( PGC-1α) gene in Guangxi Han population with gestational diabetes mellitus. Shandong Medical Journal, 2012; 52(39): 21-23. |
| [29] | Lin YC, Chang PF, Chang MH et al. A common variant in the peroxisome proliferator-activated receptor-γ coactivator-1α gene is associated with nonalcoholic fatty liver disease in obese children. Am J Clin Nutr. 2013; 97(2): 326-31. doi:10.3945/ajcn.112.046417. |
| [30] | Jin J, Ding G, Bao H et al. Correlation between PPAR Gene Polymorphisms and Primary Nephrotic Syndrome in Children. PPAR Res. 2013;2013:927915. doi: 10.1155/2013/927915. |
| [31] | Sun L, Zheng CG, Lv ZP et al.Association of peroxisome proliferator-activated receptor gamma coactivator-1 Gly482Ser with apolipo protein and the long evity and metabolic traits of Hans in Guangxi Yongfu. Chinese Journal of Geriatrics, 2013; 32( 03 ): 300-304. |
| [32] | Sun Y, Liu HJ, Cai Q et al. Correlation between Gly482Ser polymorphism of PGC-1α gene and metabolic syndrome in pilots. Acad J Chinese PLA Med Sch, 2013; 34(9): 913-918. |
| [33] | Rojek A, Cielecka-Prynda M, Przewlocka-Kosmala M et al. Impact of the PPARGC1A Gly482Ser polymorphism on left ventricular structural and functional abnormalities in patients with hypertension. J Hum Hypertens. 2014; 28(9): 557-63. doi: 10.1038/jhh.2014.26. |
| [34] | Albuquerque D, Nobrega C, Rodriguez-Lopez R et al. Association study of common polymorphisms in MSRA, TFAP2B, MC4R, NRXN3, PPARGC1A, TMEM18, SEC16B, HOXB5 and OLFM4 genes with obesity-related traits among Portuguese children. J Hum Genet. 2014; 59(6): 307-13. doi: 10.1038/jhg.2014.23. |
| [35] | Susanne RD, Lyudmyla K, Igor, et al. Does Genetic Variation in PPARGC1A Affect Exercise- Induced Changes in Ventilatory Thresholds and Metabolic Syndrome? Journal of Exercise Physiology. 2014; 17(2): 1-18. |
| [36] | Saremi L, Saremi M, Lotfipanah S, et al. Relationship between PPARGC1A Gene Polymorphisms with the Increased Risk of Coronary Artery Disease among Patients with Type 2 Diabetes Mellitus in Iran. Acta Endocrinologica (Bucharest). 2015; 11(1): 13-7. |
| [37] | Shokouhi S, Haghani K, Borji P et al. Association between PGC-1alpha gene polymorphisms and type 2 diabetes risk: a case-control study of an Iranian population. Can J Diabetes. 2015; 39(1): 65-72. doi: 10.1016/j.jcjd.2014.05.003. |
| [38] | Nishida Y, Iyadomi M, Higaki Y et al. Association between the PPARGC1A polymorphism and aerobic capacity in Japanese middle-aged men. Intern Med. 2015; 54(4): 359-66. doi:10.2169/internalmedicine.54.3170. |
| [39] | Steinbacher P, Feichtinger RG, Kedenko L et al. The single nucleotide polymorphism Gly482Ser in the PGC-1α gene impairs exercise-induced slow-twitch muscle fibre transformation in humans. PLoS One. 2015 ; 10(4): e0123881. doi:10.1371/  journal.pone.0123881. |
| [40] | Vazquez-Del Mercado M, Guzman-Ornelas MO et al. The 482Ser of PPARGC1A and 12Pro of PPARG2 Alleles Are Associated with Reduction of Metabolic Risk Factors Even Obesity in a Mexican-Mestizo Population. Biomed Res Int. 2015; 2015: 285491. doi: 10.1155/2015/285491. |
| [41] | Ha CD, Cho JK, Han T et al. Relationship of PGC-1α gene polymorphism with insulin resistance syndrome in Korean children. Asia Pac J Public Health. 2015; 27(2): NP544-51. doi: 10.1177/1010539513477685. |
| [42] | Queiroz EM, Candido AP, Castro IM et al. IGF2, LEPR, POMC, PPARG, and PPARGC1 gene variants are associated with obesity-related risk phenotypes in Brazilian children and adolescents. Braz J Med Biol Res. 2015 ; 48(7): 595-602. doi: 10.1590/1414-431X20154155. |
| [43] | Tai CM, Huang CK, Tu HP et al. Interactions of a PPARGC1α Variant and a PNPLA3 Variant Affect Nonalcoholic Steatohepatitis in Severely Obese Taiwanese Patients. Medicine (Baltimore). 2016; 95(12): e3120. doi: 10.1097/MD.0000000000003120. |
| [44] | Csep K, Szigeti E, Vitai M, Koranyi L et al. The PPARGC1α - Gly482Ser polymorphism (rs8192678) and the metabolic syndrome in a central romanian population. Acta Endocrinol (Buchar). 2017; 13(2): 161-167. doi: 10.4183/aeb.2017.161. |
| [45] | Tobina T, Mori Y, Doi Y et al. Peroxisome proliferator-activated receptor gamma co-activator 1 gene Gly482Ser polymorphism is associated with the response of low-density lipoprotein cholesterol concentrations to exercise training in elderly Japanese. J Physiol Sci. 2017; 67(5): 595-602. doi: 10.1007/s12576-016-0491-y. |
| [46] | Ramos-Lopez O, Riezu-Boj JI, Milagro FI et al. Association of the Gly482Ser PPARGC1A gene variant with different cholesterol outcomes in response to two energy-restricted diets in subjects with excessive weight. Nutrition. 2018; 47: 83-89. doi: 10.1016/j.nut.2017.10.008. |
| [47] | Reddy TV, Govatati S, Deenadayal M et al. Polymorphisms in the TFAM and PGC1-α genes and their association with polycystic ovary syndrome among South Indian women. Gene. 2018; 641: 129-136. doi: 10.1016/j.gene.2017.10.010. |
| [48] | Zehsaz F, Abbasi Soltani H, Hazrati R et al. Association between the PPARa and PPARGCA gene variations and physical performance in non-trained male adolescents. Mol Biol Rep. 2018; 45(6): 2545-2553. doi: 10.1007/s11033-018-4422-2. |
| [49] | Zhang Q, Liu SS, Sun BB et al. Association of peroxisome proliferator- activated receptor-γ coactivator-1alpha rs8192678 single nucleotide polymorphisms with the risk of nonalcoholic fatty liver disease. J Clin Hepatol, 2020; 36(9): 2035-2039. |
| [50] | Bailen M, Tabone M, Bressa C et al. Unraveling Gut Microbiota Signatures Associated with PPARD and PARGC1A Genetic Polymorphisms in a Healthy Population. Genes (Basel). 2022; 13(2): 289. doi: 10.3390/genes13020289. |
| [51] | Oguz O , Gheybi A , Dogan Z, et al. Investigation of GHRL (rs4684677), FTO (rs8044769) and PGC1Α (rs8192678) polymorphisms in type 2 diabetic Turkish population. Turkish Journal of Biochemistry. 2022; 47(5): 564-70. |

**Table S2.** Characteristics of the studies included in the meta-analysis for the MC4R rs17782313 polymorphism.

| **Authors, reference** | **Publication year** | **Ethnicity** | **Gender** | **Subjects** | **Age**  **(Mean**±SD **or age range**) | **Outcomes** |
| --- | --- | --- | --- | --- | --- | --- |
| Haupt et al. [1] | 2009 | European Caucasian | M/F | General subjects | 45.82±1.88 | BMI/WC/GLU/INS |
| Zobel et al. [2] | 2009 | European Caucasian | M/F | General subjects | 46±8 | BMI/WC/WHR/TG/TC/HDL-C/GLU/INS/HOMA-IR |
| Tabara et al. [3] | 2009 | East Asian | F | T2DM patients/general subjects | 61±14 | BMI |
| Petry et al. [4] | 2010 | European Caucasian | M/F | General subjects | 0.75±0.03 | BMI/GLU/INS |
| Liu et al. [5] | 2010 | European Caucasian | M/F | General subjects | 8-12 | BMI/WC/GLU/INS/HOMA-IR |
| Hasselbalch et al. [6] | 2010 | European Caucasian | M/F | General subjects | 18-67 | BMI/WC |
| Tschritter et al. [7] | 2011 | European Caucasian | M/F | Obesity patients | 35.06±12.3 | BMI/WCGLU |
| Orkunoglu-Suer et al. [8] | 2011 | South American | F | General subjects | 23.12±5.48 | BMI |
| Vogel et al. [9] | 2011 | European Caucasian | M/F | Overweight/obesity patients | 10.69±2.98 | WC/TG/TC/LDL-C/HDL-C/GLU/INS/HOMA-IR |
| Huang et al. [10] | 2011 | East Asian | M/F | Obesity/T2DM patients | 50-70 | BMI/WC/TG/TC/LDL-C/GLU/INS/HOMA-IR |
| Tao et al. [11] | 2012 | East Asian | M/F | T2DM/obesity patients/general subjects | 60.1±9.51 | BMI/TC/LDL-C/HDL-C |
| Valette et al. [12] | 2012 | European Caucasian | M/F | Obesity patients | 45.87±9.45 | BMI/TC/GLU |
| Corella et al. [13] | 2012 | European Caucasian | M/F | T2DM/hypertension/  dyslipidemia patients | 66.9±6.21 | BMI/WC |
| Marcadenti et al. [14] | 2013 | South American | M/F | Hypertension patients | 59.97±11.15 | BMI/WC |
| Czerwensky et al. [15] | 2013 | European Caucasian | M/F | Psychotic patients | 40.1±14.7 | BMI |
| Jaaskelainen et al. [16] | 2013 | European Caucasian | M/F | General subjects | 16 | BMI |
| Sull et al. [17] | 2013 | East Asian | M/F | General subjects | 42.9±7.8 | BMI/WC |
| Mutombo et al. [18] | 2014 | East Asian | M/F | T2DM/dyslipidemia/  hypertension/CVD patients | 66.03±13.06 | BMI/TG/LDL-C/HDL-C/GLU/HOMA-IR |
| Katsuura-Kamano et al. [19] | 2014 | East Asian | M/F | General subjects | 55.4±7.55 | BMI/TG/TC |
| Arrizabalaga et al. [20] | 2014 | European Caucasian | F | Obesity patients | 36.79±7.03 | BMI/TG/TC/GLU/INS/HOMA-IR |
| Acosta et al. [21] | 2014 | South American | M/F | Overweight/obesity patients | 37.7±11.2 | BMI |
| Yang et al. [22] | 2016 | East Asian | M/F | General subjects | 64.62±12.8 | BMI/WC/TG/TC/LDL-C/HDL-C/GLU |
| Martins et al. [23] | 2016 | South American | F | General subjects | 20-40 | BMI |
| Rotter et al. [24] | 2016 | European Caucasian | M | T2DM/hypertension/MetS /overweight/obesity patients | 62±6.4 | BMI/WC/TG/TC/LDL-C/HDL-C |
| Vega et al. [25] | 2016 | South American | M/F | Overweight/obesity patients/general subjects | 39.1±6.6 | BMI |
| Illangasekera et al. [26] | 2016 | European Caucasian | M/F | T2DM/hyperlipidemia patients/general subjects | 47.38±11.53 | BMI/WC/WHR/TG/TC/LDL-C/HDL-C/GLU |
| Tang et al. [27] | 2016 | East Asian | M/F | Obesity patients/general subjects | 13.25±3.37 | BMI/TG/TC/LDL-C/HDL-C/GLU |
| Brodowski et al. [28] | 2017 | European Caucasian | F | General subjects | 58.5±6.6 | BMI/WC/TG/TC/LDL-C/HDL-C/GLU/HOMA-IR |
| Leonska-Duniec et al. [29] | 2017 | European Caucasian | F | General subjects | 21±1 | BMI/TG/TC/LDL-C/HDL-C/GLU |
| Song et al. [30] | 2017 | East Asian | M/F | Overweight/obesity patients/general subjects | 12.67±2.77 | BMI |
| Rana et al. [31] | 2018 | South Asian | M/F | Overweight/obesity patients | 29.18±0.37 | BMI/WC/WHR/GLU/HOMA-IR |
| Almeida et al. [32] | 2018 | European Caucasian | M/F | Non-overweight/obesity subjects | 9.79±0.60 | BMI/WC/WHR/TG/TC/LDL-C/HDL-C/GLU/INS/HOMA-IR |
| Liu et al. [33] | 2018 | East Asian | M/F | Obesity patients | 36.14±11.45 | BMI/WC/WHR/TG/HDL-C/LDL-C/GLU |
| Batarfi et al. [34] | 2019 | West Asian | F | PCOS patients/general subjects | 21.5±6.72 | BMI |
| Adamska-Patruno et al. [35] | 2019 | European Caucasian | M/F | Overweight/obesity patients/general subjects | 40.22±0.47 | BMI/WHR |
| Zhang et al. [36] | 2019 | East Asian | M/F | Schizophrenia patients | 31.95±7.93 | BMI |
| Sull et al. [37] | 2020 | East Asian | M/F | CAD/T2DM patients/general subjects | 52.2±10.2 | BMI/GLU |
| Hammad et al. [38] | 2020 | West Asian | M/F | T2DM/obesity/hypertensive patients | 46.25±12.38 | BMI/TG/TC/LDL-C/HDL-C/GLU |
| Mohammadi et al. [39] | 2020 | West Asian | M/F | Obesity patients | 20-50 | TG/TC/LDL-C/HDL-C/GLU/INS/HOMA-IR |
| Khodarahmi et al. [40] | 2020 | West Asian | M/F | Obesity patients | 38.28±7.56 | BMI/WC/TG/TC/LDL-C/HDL-C/GLU/INS/HOMA-IR |
| Garavito et al. [41] | 2020 | South American | M/F | Obesity patients/general subjects | 35.3±19.95 | BMI/TG/TC/LDL-C/HDL-C/INS |
| Szkup et al. [42] | 2020 | European Caucasian | F | Coronary thrombosis/obesity /hyperglycemia/T2DM patients | 54.3±4.2 | BMI/WC/WHR/TG/TC/LDL-C/HDL-C/GLU/INS/HOMA-IR |
| Rebelos et al. [43] | 2021 | European Caucasian | M/F | T2DM/overweight patients/general subjects | 54.53±14.66 | WC/WHR |
| Farooq et al. [44] | 2021 | South Asian | M/F | Overweight/obesity patients/general subjects | 28.89±8.85 | BMI/LDL-C/HDL-C/GLU/INS |
| Raskiliene et al. [45] | 2021 | European Caucasian | M/F | Obesity/hyperglycemia /T2DM/MetS patients/general subjects | 48-49 | BMI/WC |
| Inandiklioglu et al. [46] | 2021 | European Caucasian | M/F | Obesity patients | 10.42±3.54 | TG/TC/LDL-C/HDL-C/GLU/INS/HOMA-IR |
| Adamska-Patruno et al. [47] | 2021 | European Caucasian | M/F | General subjects | 42.1±14.5 | BMI/WHR |
| Rahati et al. [48] | 2022 | West Asian | M/F | Overweight/obesity patients | 36.67±8.47 | WC/WHR |
| Alizadeh et al. [49] | 2022 | West Asian | F | Overweight/obesity patients | 36.24±8.23 | BMI/WC/WHR/TG/TC/LDL-C/HDL-C/GLU/INS/HOMA-IR |
| Hosseininasab et al. [50] | 2022 | West Asian | F | Overweight/obesity patients | 36.56±8.39 | BMI/WC/WHR/TG/TC/LDL-C/HDL-C/GLU/HOMA-IR |

MC4R, melanocortin 4 receptor; SD, standard deviation; M, male; F, female; BMI, body mass index; WC, waist circumference; WHR, waist-to-hip ratio; GLU, glucose; INS, insulin; HOMA-IR, homeostasis model assessment of insulin resistance; TG, triglyceride; TC, total cholesterol; LDL-C, low-density lipoprotein cholesterol; HDL-C, high-density lipoprotein cholesterol; T2DM, type 2 diabetes mellitus; PCOS, polycystic ovary syndrome; CAD, coronary artery disease; MetS, metabolic syndrome; CVD, cardiovascular disease.

**Table S3.** Original data of the obesity indexes by genotypes of the MC4R rs17782313 polymorphism.

| **Authors, reference** | **Subjects** | **N** | | **BMI, kg/m^2^** | | **WC, cm** | | **WHR** | |
| --- | --- | --- | --- | --- | --- | --- | --- | --- | --- |
|  |  | TT | TC+  CC | TT | TC+CC | TT | TC+CC | TT | TC+CC |
| Haupt et al. [1] | General subjects | 172 | 171 | 29.2±0.4 | 30.14±1.08 | 95.6±1.0 | 97.93±1.48 | - | - |
| Zobel et al. [2] | General subjects | 3291 | 2516 | 26.1±4.5 | 26.4±4.53 | 86±13 | 87±13.14 | 0.85±0.09 | 0.86±0.09 |
| Tabara et al. [3] | Female T2DM patients/general subjects | 1036 | 549 | 23.2±3.2 | 23.56±2.69 | - | - | - | - |
|  | Male T2DMpatients/  general subjects | 800 | 421 | 23.5±3.1 | 23.58±2.94 | - | - | - | - |
| Petry et al. [4] | General subjects | 193 | 119 | 12.8±1.42 | 12.84±1.53 | - | - | - | - |
| Liu et al. [5] | General subjects | 934 | 717 | 22.5±4.67 | 23.28±5.11 | 75.0±10.14 | 76.56±11.6 | - | - |
| Hasselbalch et al. [6] | General subjects | 849 | 547 | 24.1±3.4 | 24.69±3.69 | 83.0±10.3 | 84.21±11.02 | - | - |
| Tschritter et al. [7] | Obesity patients | 30 | 21 | 27.1±4.93 | 28.4±4.58 | 92±16.43 | 95±13.75 | - | - |
| Orkunoglu-Suer et al. [8] | General subjects | 278 | 174 | 23.41±0.26 | 24.7±0.33 | - | - | - | - |
| Vogel et al. [9] | Overweight/obesity patients | 229 | 268 | - | - | 88.69±14.26 | 88.99±13.79 | - | - |
| Huang et al. [10] | Obesity patients | 596 | 1174 | 25.2±4.9 | 27.77±5.24 | 86.9±16.7 | 89.15±16.66 | - | - |
|  | T2DM patients | 912 | 879 | - | - | - | - | - | - |
| Tao et al. [11] | T2DM/obesity patients/general subjects | 1589 | 907 | 24.95±3.4 | 25.18±3.42 | - | - | - | - |
|  | Obesity patients/general subjects | 1298 | 743 | 24.69±3.38 | 24.9±3.25 | - | - | - | - |
| Valette et al. [12] | Obesity patients | 14 | 7 | 48.8±6.3 | 49.0±7.6 | - | - | - | - |
| Corella et al. [13] | T2DM/hypertension/  dyslipidemia patients | 4336 | 2883 | 29.9±3.8 | 30.13±3.84 | 100.3±10.4 | 100.68±10.83 | - | - |
| Marcadenti et al. [14] | Female hypertension patients | 80 | 51 | 30.1±5.4 | 31.5±5.7 | 98.5±11.1 | 102.3±12.2 | - | - |
|  | Male hypertension patients | 66 | 20 | 28.9±4.4 | 30.1±4.1 | 102.5±11.1 | 105.1±11.9 | - | - |
| Czerwensky et al. [15] | Psychotic patients | 198 | 147 | 25.9±5.38 | 24.84±5.12 | - | - | - | - |
| Jaaskelainen et al. [16] | Female general subjects | 1653 | 796 | 21.1±3.11 | 21.6±3.53 | - | - | - | - |
|  | Male general subjects | 1531 | 685 | 21.1±2.99 | 21.18±3.77 | - | - | - | - |
| Sull et al. [17] | General subjects | 1317 | 964 | 23.29±2.96 | 23.59±3.08 | 79.98±9.31 | 80.3±9.67 | - | - |
| Mutombo et al. [18] | T2DM/dyslipidemia/  hypertension/CVD patients | 718 | 424 | 22.5±3.0 | 22.93±3.33 | - | - | - | - |
| Katsuura-Kamano et al. [19] | General subjects | 1268 | 767 | 22.9±3.63 | 22.94±3.27 | - | - | - | - |
| Arrizabalaga et al. [20] | Obesity patients | 53 | 24 | 34.3±2.99 | 33.2±2.3 | - | - | - | - |
| Acosta et al. [21] | Overweight/obesity patients | 94 | 84 | 33.47±5.62 | 33.36±5.05 | - | - | - | - |
| Yang et al. [22] | Female general subjects | 809 | 466 | 23.0±3.3 | 23.25±3.38 | 81.0±10.0 | 80.48±10.01 | - | - |
|  | Male general subjects | 426 | 311 | 23.2±3.3 | 23.41±3.43 | 83.3±9.5 | 83.41±9.36 | - | - |
| Martins et al. [23] | General subjects | 87 | 49 | 24.3±4.9 | 25±3.9 | - | - | - | - |
| Rotter et al. [24] | T2DM/hypertension/MetS /overweight/obesity patients | 173 | 99 | 27.75±4.32 | 28.62±4.66 | 99.77±10.86 | 103.91±12.61 | - | - |
| Vega et al. [25] | Overweight/obesity patients/general subjects | 234 | 92 | 30.1±6.0 | 31.12±7.17 | - | - | - | - |
| Illangasekera et al. [26] | T2DM/hyperlipidemia patients/general subjects | 233 | 295 | 24.6±4.1 | 25.4±4.2 | 88.8±11.1 | 90.0±10.5 | 0.92±0.07 | 0.93±0.07 |
| Tang et al. [27] | Obesity patients/general subjects | 194 | 91 | 23.33±5.50 | 23.67±5.45 | - | - | - | - |
| Brodowski et al. [28] | General subjects | 227 | 118 | 27.2±4.6 | 27.7±4.0 | 87.8±11.3 | 88.4±9.4 | - | - |
| Leonska-Duniec et al. [29] | General subjects | 123 | 78 | 21.7±2.4 | 21.5±2.3 | - | - | - | - |
| Song et al. [30] | Overweight/obesity patients/general subjects | 1202 | 828 | 23.70±4.90 | 23.99±4.64 | - | - | - | - |
| Rana et al. [31] | Overweight/obesity patients | 90 | 180 | 25.76±6.42 | 28.97±8.18 | 91.44±17.07 | 100.27±20.57 | 0.89±0.092 | 0.93±0.1 |
| Almeida et al. [32] | Overweight/obesity patients | 76 | 38 | 23.92±2.75 | 22.82±2.36 | 79.5±9.07 | 75.55±8.47 | 0.93±0.07 | 0.92±0.06 |
|  | Non-overweight/obesity controls | 132 | 80 | 16.74±1.37 | 16.8±1.72 | 61.3±4.76 | 61.8±5.82 | 0.87±0.05 | 0.85±0.06 |
| Liu et al. [33] | Female obesity patients | 25 | 26 | 36.41±4.07 | 38.54±6.08 | 114.3±17.10 | 121.10±28.80 | 0.95±0.09 | 1.01±0.13 |
|  | Male obesity patients | 16 | 14 | 38.41±6.19 | 39.96±7.38 | 135.33±20.95 | 129.33±15.76 | 0.98±0.06 | 1.00±0.07 |
| Batarfi et al. [34] | PCOS patients | 49 | 46 | 23.5±7.1 | 25.74±6.83 | - | - | - | - |
|  | Non-PCOS controls | 56 | 38 | 23±5.8 | 21.63±8.27 | - | - | - | - |
| Adamska-Patruno et al. [35] | Overweight/obesity patients/general subjects | 584 | 343 | 27.90±0.27 | 28.59±0.66 | - | - | 0.92±0.00 | 0.93±0.01 |
| Zhang et al. [36] | Schizophrenia patients  (drug-naive patients) | 354 | 213 | 21.29±3.12 | 21.36±3.26 | - | - | - | - |
|  | Schizophrenia patients  (medicated patients) | 913 | 511 | 22.38±3.66 | 22.86±3.63 | - | - | - | - |
| Sull et al. [37] | CAD/T2DM patients/  general subjects | 2428 | 1831 | 24.3±2.9 | 24.54±3.03 | - | - | - | - |
| Hammad et al. [38] | T2DM/obesity/hypertensive patients | 203 | 79 | 29.39±5.12 | 30.52±5.12 | - | - | - | - |
| Khodarahmi et al. [40] | Obesity patients | 63 | 78 | 34.5±3.6 | 34.37±3.25 | 108.9±10.0 | 107.62±9.23 | - | - |
| Garavito et al. [41] | Obesity patients | 83 | 26 | 33.04±5.4 | 33.33±3.5 | - | - | - | - |
|  | Non-obesity controls | 116 | 39 | 22.6±2.5 | 22.62±2.82 | - | - | - | - |
| Szkup et al. [42] | Coronary thrombosis/obesity /hyperglycemia/T2DM patients | 274 | 151 | 28.1±5.3 | 27.63±5.05 | 88±10.8 | 87.27±10.42 | 0.9±0.1 | 0.81±0.1 |
| Rebelos et al. [43] | T2DM/overweight patients/general subjects | 79 | 74 | - | - | 97±17 | 97.08±19 | 0.89±0.08 | 0.89±0.08 |
| Farooq et al. [44] | Overweight/obesity patients/general subjects | 183 | 359 | 25.605±6.083 | 27.03±7.08 | - | - | - | - |
| Raskiliene et al. [45] | Female obesity/  hyperglycemia/T2DM/MetS patients/general subjects | 190 | 89 | 25.4±6.0 | 26.4±9.0 | 81.1±15.1 | 85.0±17.3 | - | - |
|  | Male obesity/  hyperglycemia/T2DM/MetS patients/general subjects | 154 | 76 | 26.1±5.9 | 28.2±6.7 | 95.0±17.2 | 99.0±17.8 | - | - |
| Adamska-Patruno et al. [47] | General subjects | 504 | 305 | 28.1±6.5 | 29.08±6.85 | - | - | 0.92±0.088 | 0.94±0.09 |
| Rahati et al. [48] | Overweight patients | 71 | 150 | - | - | 93.6±7.6 | 94.88±6.83 | 0.92±0.1 | 0.93±0.09 |
|  | Obesity patients | 29 | 153 | - | - | 102.7±8 | 104.51±7.32 | 0.95±0.1 | 0.97±0.01 |
| Alizadeh et al. [49] | Overweight/obesity patients | 153 | 129 | 31.06±4.66 | 30.78±3.90 | 99.16±10.31 | 98.53±9.81 | 0.93±0.05 | 1.64±8.00 |
| Hosseininasab et al. [50] | Overweight/obesity patients | 152 | 128 | 31.06±4.66 | 30.78±3.90 | 99.16±10.31 | 98.53±9.81 | 0.93±0.05 | 1.64±8.04 |

MC4R, melanocortin 4 receptor; SD, standard deviation; BMI, body mass index; WC, waist circumference; WHR, waist-to-hip ratio;T2DM, type 2 diabetes mellitus; PCOS, polycystic ovary syndrome; CAD, coronary artery disease; MetS, metabolic syndrome; CVD, cardiovascular disease.

**Table S4.** Original data of the glucometabolic disorder indexes by genotypes of the MC4R rs17782313 polymorphism.

| **Authors, reference** | **Subjects** | **N** | | **GLU, mg/dL** | | **INS, μU/mL** | | **HOMA-IR** | |
| --- | --- | --- | --- | --- | --- | --- | --- | --- | --- |
|  |  | TT | TC+CC | TT | TC+CC | TT | TC+CC | TT | TC+CC |
| Haupt et al. [1] | General subjects | 172 | 171 | 93.6±1.8 | 95.4±1.79 | 171.57±7.18 | 177.68±19.26 | - | - |
| Zobel et al. [2] | General subjects | 3291 | 2516 | 99±14.4 | 100.54±13.96 | 6.03±4.02 | 6.09±3.97 | 10.5±8.2 | 10.67±7.75 |
| Petry et al. [4] | General subjects | 193 | 119 | 75.6±19.08 | 77.4±18.54 | 5.46±4.58 | 5.99±4.76 |  |  |
| Liu et al. [5] | General subjects | 449 | 350 | 91.8±9.72 | 91.8±7.72 | 14.04±6.75 | 13.96±7.98 | 1.8±1.07 | 1.71±1.26 |
| Tschritter et al. [7] | Obesity patients | 30 | 21 | 88.2±9.9 | 91.8±8.28 | - | - | - | - |
| Vogel et al. [9] | Overweight/obesity patients | 393 | 405 | - | - | 1.12±0.31 | 1.13±0.3 | 3.59±3.47 | 3.65±2.84 |
| Huang et al. [10] | T2DM patients | 912 | 879 | 100.08±1.98 | 99.65±2.44 | 12.29±0.11 | 11.82±0.43 | 3.38±4.11 | 3.7±3.79 |
| Valette et al. [12] | Obesity patients | 14 | 7 | 113.4±30.6 | 129.6±48.6 | - | - | - | - |
| Mutombo et al. [18] | T2DM/dyslipidemia/  hypertension/CVD patients | 718 | 424 | 94.8±12.1 | 96.22±16.11 | - | - | 0.91±1.34 | 1.18±2.35 |
| Arrizabalaga et al. [20] | Obesity patients | 53 | 24 | 90±8 | 90±7 | 9.0±5.6 | 8.3±2.9 | 2.04±1.28 | 1.88±0.78 |
| Yang et al. [22] | Female general subjects | 809 | 466 | 96.6±12.4 | 97.1±17.88 | - | - | - | - |
|  | Male general subjects | 426 | 311 | 100±23.5 | 101±21.36 | - | - | - | - |
| Illangasekera et al. [26] | T2DM/hyperlipidemia patients/general subjects | 233 | 295 | 91.5±35.0 | 100.4±49.0 | - | - | - | - |
| Tang et al. [27] | Obesity patients/general subjects | 194 | 91 | 79.02±10.62 | 80.43±12.26 | - | - | - | - |
| Brodowski et al. [28] | General subjects | 227 | 118 | 90.3±13.8 | 89.3±13.0 | - | - | 2.4±1.7 | 2.2±1.0 |
| Leonska-Duniec et al. [29] | General subjects | 123 | 78 | 76.81±9.2 | 79.8±11.0 | - | - | - | - |
| Rana et al. [31] | Overweight/obesity patients | 90 | 180 | 101.63±13.85 | 104.56±11.09 | - | - | 5.92±3.71 | 6.13±3.55 |
| Almeida et al. [32] | Overweight/obesity patients | 76 | 38 | 79.3±11.0 | 80.83±9.34 | 11.72±21.80 | 7.71±6.99 | 2.30±4.50 | 1.66±1.63 |
|  | Non-overweight/obesity  controls | 132 | 80 | 77.3±10.0 | 77.13±10.2 | 5.88±5.37 | 4.97±3.19 | 1.18±1.19 | 0.99±0.74 |
| Liu et al. [33] | Female obesity patients | 25 | 26 | 128.7±50.76 | 144.54±53.28 | - | - | - | - |
|  | Male obesity patients | 16 | 14 | 141.3±61.92 | 140.4±60.66 | - | - | - | - |
| Sull et al. [37] | CAD/T2DM patients/  general subjects | 2428 | 1831 | 96.0±21.3 | 98.31±24.11 | - | - | - | - |
| Hammad et al. [38] | T2DM/obesity/hypertensive patients | 203 | 79 | 102.69±22.50 | 105.46±22.14 | - | - | - | - |
| Mohammadi et al. [39] | Obesity patients | 114 | 174 | 93.70±13.42 | 95.58±32.15 | 16.78±8.81 | 16.24±10.29 | 3.53±2.01 | 4.11±3.09 |
| Khodarahmi et al. [40] | Female obesity patients | 28 | 44 | 92.00±12.57 | 90.64±21.01 | 13.55±19.11 | 17.47±5.36 | 3.17±4.59 | 4.21±4.81 |
|  | Male obesity patients | 35 | 34 | 93±21.83 | 93.24±32.67 | 11.40±12.08 | 10.93±12.25 | 2.70±3.42 | 3.11±3.57 |
| Garavito et al. [41] | Obesity patients | 83 | 26 | - | - | 3.96±9.2 | 8.75±11.69 | - | - |
|  | Non-obesity controls | 116 | 39 | - | - | 4.2±7.4 | 3.05±7.04 | - | - |
| Szkup et al. [42] | Coronary thrombosis/obesity /hyperglycemia/T2DM patients | 274 | 151 | 96.1±32 | 92.96±26.46 | 11.1±6.8 | 11.2±6.49 | 2.9±3 | 2.76±2.57 |
| Farooq et al. [44] | Overweight/obesity patients/general subjects | 183 | 359 | 98.852±13.121 | 102.95±18.26 | 21.663±11.626 | 21.6±12.85 | - | - |
| Inandiklioglu et al. [46] | Obesity patients | 50 | 50 | 89.02±8.01 | 88.18±7.72 | 13.64±10.28 | 13.73±9.36 | 3.11±2.90 | 3.04±2.32 |
| Alizadeh et al. [49] | Overweight/obesity patients | 153 | 129 | 87.94±10.21 | 86.24±8.46 | 1.24±0.22 | 1.19±0.24 | - | - |
| Hosseininasab et al. [50] | Overweight/obesity patients | 152 | 128 | 87.94±10.21 | 86.24±8.46 | - | - | 3.36±1.36 | 3.27±1.17 |

MC4R, melanocortin 4 receptor; SD, standard deviation; GLU, glucose; INS, insulin; HOMA-IR, homeostasis model assessment of insulin resistance; T2DM, type 2 diabetes mellitus; PCOS, polycystic ovary syndrome; CAD, coronary artery disease; MetS, metabolic syndrome; CVD, cardiovascular disease.

**Table S5.** Original data of the dyslipidemia indexes by genotypes of the MC4R rs17782313 polymorphism.

| **Authors, reference** | **Subjects** | **N** | | **TG, mg/dL** | | **TC, mg/dL** | | **LDL-C, mg/dL** | | **HDL-C, mg/dL** | |
| --- | --- | --- | --- | --- | --- | --- | --- | --- | --- | --- | --- |
|  |  | TT | TC+  CC | TT | TC+CC | TT | TC+CC | TT | TC+CC | TT | TC+CC |
| Zobel et al. [2] | General subjects | 3291 | 2516 | 115.11±141.67 | 115.11±94.08 | 212.69±42.54 | 212.69±42.01 | - | - | 54.14±15.47 | 54.69±15.53 |
| Vogel et al. [9] | Overweight/obesity patients | 403 | 420 | 173.55±19.48 | 174.56±18.68 | 85.85±3.09 | 86.16±3.09 | 77.34±5.03 | 77.03±5.19 | 64.97±3.87 | 65.19±3.8 |
| Huang et al. [10] | T2DM patients | 912 | 879 | 202.77±169.12 | 192.19±123.76 | 170.15±58.39 | 160.73±61.37 | 120.26±77.73 | 121.3±68.14 | - | - |
| Tao et al. [11] | T2DM/obesity patients  /general subjects | 1589 | 907 | - | - | 193.74±36.35 | 188.91±36.42 | 118.33±30.55 | 114.27±29.69 | 46.02±11.6 | 45.39±11.05 |
|  | Obesity patients/  general subjects | 1298 | 743 | - | - | 175.95±34.8 | 173.73±34.16 | 110.6±28.62 | 108.59±28.1 | 47.56±11.6 | 47.3±12.03 |
| Valette et al. [12] | Obesity patients | 14 | 7 | - | - | 174.02±30.94 | 174.02±30.94 | - | - | - | - |
| Mutombo et al. [18] | T2DM/dyslipidemia/  hypertension/CVD patients | 718 | 424 | 105.0±60.7 | 105.35±53.33 | - | - | 120.3±27.8 | 120.36±25.86 | 61.2±15.2 | 59.78±14.53 |
| Katsuura-Kamano et al. [19] | General subjects | 1268 | 767 | 93.3±60.86 | 97.98±57.56 | 206.5±41.79 | 204.47±37.66 | - | - | 64.1±19.98 | 63.23±17.56 |
| Arrizabalaga et al. [20] | Obesity patients | 53 | 24 | 101±6 | 91±9 | 194±33 | 186±29 | - | - | - | - |
| Yang et al. [22] | Female general subjects | 809 | 466 | 106±55.8 | 109.85±60.33 | 207±32.4 | 213.15±32.78 | 126±28.1 | 129.45±29 | 99.4±54.7 | 91.49±52.21 |
|  | Male general subjects | 426 | 311 | 124±105 | 116.31±84.5 | 198±33.2 | 199.11±30.74 | 113±28.2 | 116±27.36 | 97.1±52.2 | 100.36±50.17 |
| Rotter et al. [24] | T2DM/hypertension/MetS/overweight/obesity patients | 173 | 99 | 142.59±83.86 | 146.95±75.57 | 211.24±57.03 | 211.57±59.93 | 140.29±53.71 | 140.93±57.36 | 42.61±12.9 | 42.42±13.17 |
| Illangasekera et al. [26] | T2DM/hyperlipidemia patients/general subjects | 233 | 295 | 128.5±65.7 | 128.2±66.4 | 221.8±46.4 | 217.5±46.3 | 143.4±42.4 | 138.8±41.2 | 53.4±11.8 | 53.6±11.9 |
| Tang et al. [27] | Obesity patients/  general subjects | 194 | 91 | 93.86±69.95 | 83.71±41.38 | 162.8±30.94 | 169.02±33.17 | 95.13±26.68 | 98.25±26.93 | 55.3±11.21 | 57.11±15.76 |
| Brodowski et al. [28] | General subjects | 227 | 118 | 118.7±52.1 | 131.3±72.8 | 218.8±41.4 | 227.1±44.8 | 133.5±37.7 | 144.9±36.3 | 60.3±15.7 | 58.9±15.2 |
| Leonska-Duniec et al. [29] | General subjects | 123 | 78 | 80.0±34.5 | 76.4 ± 25.8 | 170±27 | 167±24 | 89.5±21.5 | 86.1±21.0 | 64.0±12.2 | 66.1±13.7 |
| Almeida et al. [32] | Overweight/obesity patients | 76 | 38 | 81.3±35.8 | 76.87±32.7 | 170.1±30.0 | 171.79±27.6 | 98.9±27.0 | 98.7±24.16 | 50.0±8.6 | 51.52±9.97 |
|  | Non-overweight/obesity controls | 132 | 80 | 53.1±20.3 | 60.1±22.43 | 170.0±32.8 | 173.04±29.55 | 86.4±24.1 | 89.9±23.02 | 59.7±12.0 | 54.92±8.78 |
| Liu et al. [33] | Female obesity patients | 25 | 26 | 155.84±61.1 | 161.15±58.44 | - | - | 76.57±27.84 | 105.57±50.27 | 73.09±35.19 | 59.55±25.14 |
|  | Male obesity patients | 16 | 14 | 209.85±113.34 | 311.68±214.28 | - | - | 117.56±65.35 | 151.2±77.34 | 47.95±27.07 | 38.67±20.11 |
| Hammad et al. [38] | T2DM/obesity/hypertensive patients | 203 | 79 | 102.80±51.36 | 115.11±54.01 | 192.69±43.70 | 195.98±40.99 | 119.45±36.35 | 122.82±38.28 | 46.79±12.76 | 46.09±11.99 |
| Mohammadi et al. [39] | Obesity patients | 114 | 174 | 117.78±56.47 | 119.9±54.79 | 183.20±30.91 | 193.2±36.32 | 114.89±27.23 | 124.04±35.29 | 44.75±9.44 | 45.64±9.2 |
| Khodarahmi et al. [40] | Female obesity patients | 28 | 44 | 111.75±46.33 | 101.5±39.29 | 197.71±33.88 | 182.83±33.01 | 126.9±29.60 | 115.05±30.05 | 48.46±10.27 | 47.49±9.05 |
|  | Male obesity patients | 35 | 34 | 122±129.54 | 121.65±79.78 | 188.4±28.39 | 186.59±34.95 | 120.46±26.31 | 115.16±29.43 | 41.11±6.64 | 43.71±9.22 |
| Garavito et al. [41] | Obesity patients | 83 | 26 | 141±129 | 146.14±92.13 | 197±69 | 195.38±35.44 | 124±57.2 | 124.28±45.92 | 41±15 | 48.31±17.5 |
|  | Non-obesity controls | 116 | 39 | 121.5±102 | 127.41±67.22 | 192±58.5 | 175.05±65.44 | 120±52.8 | 107.91±49.72 | 40.5±15 | 42.49±16.92 |
| Szkup et al. [42] | Coronary thrombosis/obesity /hyperglycemia/T2DM patients | 274 | 151 | 112.9±57.4 | 126.43±74.89 | 215.8±40 | 223.3±48.34 | 136.7±64.8 | 147.78±62.38 | 63.3±16.9 | 62.37±17 |
| Farooq et al. [44] | Overweight/obesity patients/general subjects | 183 | 359 | - | - | - | - | 92.27±36.31 | 88.71±36.48 | 30.830±9.33 | 29.98±9.34 |
| Inandiklioglu et al. [46] | Obesity patients | 50 | 50 | 131.84±76.71 | 101.82±82.75 | 165.88±25.62 | 160.92±24.23 | 96.23±23.99 | 88.58±18.71 | 42.84±6.64 | 44.45±10.23 |
| Alizadeh et al. [49] | Overweight/obesity patients | 153 | 129 | 127.89±74.23 | 114.52±64.32 | 185.19±34.12 | 184.83±38.08 | 95.81±23.23 | 93.84±25.24 | 46.35±9.94 | 47.07±11.98 |
| Hosseininasab et al. [50] | Overweight/obesity patients | 152 | 128 | 127.89±74.23 | 114.52±64.32 | 185.19±34.12 | 184.83±38.08 | 95.81±23.23 | 93.84±25.24 | 46.35±9.94 | 47.07±11.98 |

MC4R, melanocortin 4 receptor; SD, standard deviation; TG, triglyceride; TC, total cholesterol; LDL-C, low-density lipoprotein cholesterol; HDL-C, high-density lipoprotein cholesterol; T2DM, type 2 diabetes mellitus; PCOS, polycystic ovary syndrome; CAD, coronary artery disease; MetS, metabolic syndrome; CVD, cardiovascular disease.

**Table S6.** Characteristics of the studies included in the meta-analysis for the PGC1α rs8192678 polymorphism.

| **Authors, reference** | **Publication year** | **Ethnicity** | **Gender** | **Subjects** | **Age**  **(Mean**±SD **or age range**) | **Outcomes** |
| --- | --- | --- | --- | --- | --- | --- |
| Ek et al. [1] | 2001 | European Caucasian | M/F | General subjects | 57.18±9.73 | BMI/TG/TC/HDL-C/GLU/INS |
| Hara et al. [2] | 2002 | East Asian | M/F | Non-diabetic subjects | 67.9±6.85 | BMI/GLU/INS/HOMA-IR |
| Stumvoll et al. [3] | 2004 | European Caucasian | M/F | General subjects | 35.02±12.73 | BMI/WHR/GLU/INS |
| Wang et al. [4] | 2004 | East Asian | M/F | T2DM patients | 55.59±11.24 | BMI/TG/TC/LDL-C/HDL-C/GLU |
| Ambye et al. [5] | 2005 | European Caucasian | M/F | General subjects | 41-71 | BMI/WC/TG/TC/LDL-C/HDL-C/GLU/INS/HOMA-IR |
| Vohl et al. [6] | 2005 | American Caucasian | M/F | T2DM/obesity patients | 42.54±10.2 | BMI/WC/TG/TC/LDL-C/HDL-C/GLU |
| Wang et al. [7] | 2005 | East Asian | M/F | General subjects | Not reported | BMI/TG/TC/LDL-C/HDL-C/GLU/INS |
| Wang et al. [8] | 2006 | East Asian | F | PCOS patients | 26.27±3.79 | BMI |
| Lu et al. [9] | 2006 | East Asian | M/F | T2DM patients | 62±7 | TG/TC/LDL-C/HDL-C |
| Shan et al. [10] | 2006 | East Asian | M/F | T2DM patients/control subjects | Not reported | BMI/WHR/GLU/INS |
| Zhang et al. [11] | 2007 | East Asian | M/F | T2DM patients/control subjects | 62.4±5.37 | BMI/TG/TC/LDL-C/HDL-C/GLU |
| Song et al. [12] | 2007 | East Asian | M/F | T2DM/overweight/obesity patients/control subjects | 44.69±13.11 | BMI/WHR/TG/TC/LDL-C/HDL-C/INS/HOMA-IR |
| Ke et al. [13] | 2007 | East Asian | F | PCOS patients | 26.1±4.0 | BMI |
| Hui et al. [14] | 2008 | East Asian | M/F | NAFLD patients/control subjects | 50.5±11.36 | BMI/WHR/TG/TC/LDL-C/HDL-C/GLU/INS/HOMA-IR |
| Okauchi et al. [15] | 2008 | East Asian | M/F | T2DM patients | 61.89±10.31 | BMI/TG/TC/HDL-C/GLU/INS |
| Goyenechea et al. [16] | 2008 | European Caucasian | M/F | Obesity patients | 35.03±6.04 | BMI/WC/TG/TC/LDL-C/HDL-C/GLU/INS/HOMA-IR |
| Ingelsson et al. [17] | 2008 | European Caucasian | M/F | General subjects | 50.15±12.04 | BMI |
| Hui et al. [18] | 2009 | East Asian | M/F | T2DM patients/control subjects | 62.39±10.62 | BMI |
| Chen et al. [19] | 2009 | East Asian | M/F | Hypertension/T2DM patients | 57.27±9.46 | TG/TC/LDL-C/HDL-C |
| Weng et al. [20] | 2010 | East Asian | M/F | T2DM/obesity patients/control subjects | 55.63±10.56 | BMI/WC/WHR/INS/HOMA-IR |
| Chae et al. [21] | 2010 | East Asian | F | PCOS patients/control subjects | 27.3±3.97 | BMI/WHR/TG/TC/HDL-C/GLU/INS/HOMA-IR |
| Zhang et al. [22] | 2010 | East Asian | M/F | T2DM patients | 49.28±9.63 | BMI/WHR/TG/TC/LDL-C/HDL-C/GLU/INS/HOMA-IR |
| Nikitin et al. [23] | 2010 | European Caucasian | M/F | CAD patients/control subjects | 58.1±8.4 | BMI/TG/TC/LDL-C/HDL-C |
| Chiu et al. [24] | 2012 | East Asian | F | General subjects | 16-18 | BMI |
| Geloneze et al. [25] | 2012 | South American | M/F | Obesity patients | 37.2±9.94 | BMI/WC/WHR/TG/TC/LDL-C/HDL-C/GLU/INS/HOMA-IR |
| Mirzaei et al. [26] | 2012 | West Asian | M/F | Obesity patients/control subjects | 36.94±11.61 | BMI/TG/TC/LDL-C/HDL-C/GLU/INS/HOMA-IR |
| Deng et al. [27] | 2012 | East Asian | M/F | IGR patients | 54.01±10.92 | BMI/TG/TC/LDL-C/HDL-C |
| Pang et al. [28] | 2012 | East Asian | F | GMD patients/control subjects | 29.54±4.02 | BMI |
| Lin et al. [29] | 2013 | East Asian | M/F | Obesity patients | 11.12±2.13 | BMI/WC/WHR/TG/TC/HDL-C/GLU/INS/HOMA-IR |
| Jin et al. [30] | 2013 | East Asian | M/F | PNS patients | 0.67-13.08 | BMI/TG/TC/HDL-C/GLU/HOMA-IR |
| Sun et al. [31] | 2013 | East Asian | M/F | General subjects | 94.7±3.3 | BMI/WHR/TG/TC/LDL-C/HDL-C/GLU |
| Sun et al. [32] | 2013 | East Asian | M/F | MetS patients | 22-55 | BMI/WC/TG/HDL-C/GLU/INS/HOMA-IR |
| Rojek et al. [33] | 2014 | European Caucasian | M/F | Hypertensive patients | 59.63±9.57 | BMI/TG/TC/LDL-C/HDL-C/GLU |
| Albuquerque et al. [34] | 2014 | European Caucasian | M/F | General subjects | 6-12 | BMI/WC |
| Susanne et al. [35] | 2014 | European Caucasian | M | Overweight/obesity patients | 58.3±5.71 | BMI/WC/TG/HDL-C/GLU |
| Saremi et al. [36] | 2015 | West Asian | M/F | CAD patients/control subjects | 52.6±9.59 | BMI/TG/TC/LDL-C/HDL-C/GLU |
| Shokouhi et al. [37] | 2015 | West Asian | M/F | T2DM patients/control subjects | 54.14±10.44 | BMI/WHR/TG/TC/LDL-C/HDL-C/GLU/INS/HOMA-IR |
| Nishida et al. [38] | 2015 | East Asian | M | General subjects | 47.15±4.17 | BMI/WC/TG/TC/LDL-C/HDL-C/GLU |
| Steinbacher et al. [39] | 2015 | European Caucasian | M | General subjects | 58.66±1.87 | BMI/WC |
| Vazquez-Del Mercado et al. [40] | 2015 | South American | M/F | Overweight/obesity patients/control subjects | >18 | BMI/WC/WHR/TG/TC/LDL-C/HDL-C/GLU/INS/HOMA-IR |
| Ha et al. [41] | 2015 | East Asian | M/F | General subjects | 10.13±1.78 | BMI/WC/TG/TC/HDL-C/GLU/INS/HOMA-IR |
| Queiroz et al. [42] | 2015 | South American | M/F | General subjects | 7-14 | TG/LDL-C/GLU |
| Tai et al. [43] | 2016 | East Asian | M/F | Obesity patients | 30.31±9.71 | BMI/TG/TC/LDL-C/HDL-C/GLU/INS/HOMA-IR |
| Csep et al. [44] | 2017 | European Caucasian | M/F | MetS patients/control subjects | 58.37±13.25 | TG/TC/GLU |
| Tobina et al. [45] | 2017 | East Asian | M/F | General subjects | 71.2±6.53 | BMI/TG/TC/LDL-C/HDL-C/GLU/INS/HOMA-IR |
| Ramos-Lopez etal. [46] | 2018 | European Caucasian | M/F | Obesity patients | 46.76±9.78 | BMI/WC/TG/TC/LDL-C/HDL-C/  GLU |
| Reddy et al. [47] | 2018 | South Asian | F | PCOS patients | 28.36±5.82 | BMI |
| Zehsaz et al. [48] | 2018 | West Asian | M | General subjects | 13.24±0.29 | BMI |
| Zhang et al. [49] | 2020 | East Asian | M/F | NAFLD patients/control subjects | 43.87±10.52 | BMI/TC/LDL-C/HDL-C |
| Bailen et al. [50] | 2022 | European Caucasian | M/F | General subjects | 33.54±7.61 | BMI |
| Oguz et al. [51] | 2022 | European Caucasian | M/F | T2DM patients | 56.58±10.23 | BMI/TG/TC/LDL-C/HDL-C/GLU |

PGC1α, peroxisome proliferator-activated receptor alpha; SD, standard deviation; M, male; F, female; BMI, body mass index; WC, waist circumference; WHR, waist-to-hip ratio; GLU, glucose; INS, insulin; HOMA-IR, homeostasis model assessment of insulin resistance; TG, triglyceride; TC, total cholesterol; LDL-C, low-density lipoprotein cholesterol; HDL-C, high-density lipoprotein cholesterol; T2DM, type 2 diabetes mellitus; PCOS, polycystic ovary syndrome; NAFLD, nonalcoholic fatty liver disease; CAD, coronary artery disease; IGR, impaired glucose regulation; GMD, gestational diabetes mellitus; PNS, primary nephrotic syndrome; MetS, metabolic syndrome.

**Table S7.** Original data of the obesity indexes by genotypes of the PGC1α rs8192678 polymorphism.

| **Authors, reference** | **Subjects** | **N** | | **BMI, kg/m^2^** | | **WC, cm** | | **WHR** | |
| --- | --- | --- | --- | --- | --- | --- | --- | --- | --- |
|  |  | GG | AA+AG | GG | AA+AG | GG | AA+AG | GG | AA+AG |
| Ek et al. [1] | General subjects | 243 | 248 | 25.3±3.61 | 25.86±3.97 | - | - | - | - |
| Hara et al. [2] | Non-diabetic subjects | 178 | 359 | 23.9±3.34 | 23.95±3.33 | - | - | - | - |
| Stumvoll et al. [3] | General subjects | 267 | 359 | 25.75±6.15 | 25.25±6.2 | - | - | 0.84±0.12 | 0.84±0.12 |
| Wang et al. [4] | T2DM patients | 6 | 14 | 23.87±1.56 | 23.62±2.52 | - | - | - | - |
| Ambye et al. [5] | General subjects | 931 | 1324 | 25.8±4.1 | 25.84±4.17 | 87.3±12.0 | 87.2±12.45 | - | - |
| Vohl et al. [6] | T2DM/obesity patients | 37 | 51 | 51.0±9.1 | 53.1±10.32 | 145.1±18.1 | 145.04±19.22 | - | - |
|  | Obesity patients | 70 | 112 | 53.1±9.3 | 53.16±10.5 | 139.6±18.9 | 140.5±19.48 | - | - |
| Wang et al. [7] | General subjects | 41 | 70 | 24.76±2.95 | 25.63±4.46 | - | - | - | - |
| Wang et al. [8] | PCOS patients | 24 | 93 | 22.9±4.3 | 22.24±3.3 | - | - |  |  |
| Shan et al. [10] | T2DM patients | 16 | 42 | 20.94±1.32 | 20.85±1.53 | - | - | 0.80±0.08 | 0.85±0.1 |
|  | Non-T2DM controls | 36 | 86 | 21.12±1.26 | 21.06±1.25 | - | - | 0.79±0.06 | 0.78±0.07 |
| Zhang et al. [11] | T2DM patients | 97 | 166 | 24.3±2.42 | 23.98±2.6 | - | - | - | - |
|  | Non-T2DM controls | 144 | 138 | 23.98±3.17 | 23.25±3.16 | - | - | - | - |
| Song et al. [12] | T2DM/overweight/obesity patients | 29 | 70 | 28.48±2.69 | 28.30±2.51 | - | - | 0.98±0.11 | 0.93±0.08 |
|  | Overweight/obesity patients | 24 | 68 | 29.19±3.09 | 29.44±3.3 | - | - | 0.91±0.05 | 0.90±0.08 |
|  | Non-T2DM/overweight/obesity controls | 33 | 70 | 21.39±1.84 | 21.66±2.18 | - | - | 0.82±0.06 | 0.82±0.08 |
| Ke et al. [13] | PCOS patients | 24 | 93 | 22.9±4.3 | 22.24±3.3 | - | - | - | - |
| Hui et al. [14] | NAFLD patients | 41 | 55 | 25.1±2.3 | 24.6±1.7 | - | - | 0.9±0.1 | 0.9±0.1 |
|  | Non-NAFLD controls | 33 | 63 | 24.5±2.4 | 24.3±1.9 | - | - | 0.8±0.1 | 0.8±0.1 |
| Okauchi et al. [15] | Male T2DM patients | 21 | 53 | 23.0±3.5 | 25.15±6.27 | - | - | - | - |
|  | Female T2DM patients | 21 | 60 | 25.80±3.8 | 25.06±5.31 | - | - | - | - |
| Goyenechea et al. [16] | Obesity patients | 73 | 107 | 31.5±3.5 | 31±2.69 | 97.1±10.5 | 96.3±7.96 | - | - |
| Ingelsson et al. [17] | Male general subjects | 214 | 285 | 26.9±3.4 | 27.2±3.41 | - | - | - | - |
|  | Female general subjects | 229 | 304 | 26.8±5.1 | 27.32±5.09 | - | - | - | - |
| Hui et al. [18] | T2DM patients | 28 | 112 | 24.6±3.4 | 24.18±3.32 | - | - | - | - |
|  | Non-T2DM controls | 30 | 58 | 25.7±4.0 | 25.35±3.74 | - | - | - | - |
| Weng et al. [20] | T2DM patients | 91 | 185 | 25.6±3.6 | 26.34±3.72 | - | - | 0.923±0.065 | 0.92±0.07 |
|  | Obesity patients | 155 | 312 | 27.7±2.5 | 27.83±2.33 | - | - | 0.915±0.07 | 0.93±0.07 |
|  | Non-obesity controls | 185 | 397 | 22.2±1.8 | 22.4±1.92 | - | - | 0.881±0.074 | 0.88±0.07 |
|  | T2DM/obesity patients/general subjects | 431 | 894 | - | - | 85.2 ± 9.7 | 85.65±10.47 | - | - |
| Chae et al. [21] | PCOS patients | 46 | 138 | 23.0±5.5 | 21.87±4.53 | - | - | 0.8±0.1 | 0.8±0.1 |
|  | Non-PCOS controls | 72 | 184 | 20.3±2.5 | 20.24±3.02 | - | - | 0.7±0.1 | 0.7±0.1 |
| Zhang et al. [22] | T2DM patients | 70 | 171 | 25.23±3.15 | 24.85±3.21 | - | - | 0.89±0.05 | 0.9±0.07 |
| Nikitin et al. [23] | CAD patients | 135 | 178 | 27.9±5.0 | 28.75±5.21 | - | - | - | - |
|  | Non-CAD controls | 54 | 78 | 29.8±6.0 | 28.86±6.72 | - | - | - | - |
| Chiu et al. [24] | General subjects | 41 | 129 | 22.0±3.20 | 21.42±3.22 | - | - | - | - |
| Geloneze et al. [25] | Obesity patients | 26 | 29 | 45±6 | 44±5 | 123±11 | 121±11 | 0.91±0.08 | 0.93±0.05 |
| Mirzaei et al. [26] | Obesity patients/controls | 106 | 123 | 30.33±6.99 | 30.18±6.1 | - | - | - | - |
| Deng et al. [27] | IGR patients | 156 | 205 | 24.81±3.29 | 23.88±3.41 | - | - | - | - |
| Pang et al. [28] | GMD patients | 22 | 62 | 21.64±2.1 | 20.98±2.48 | - | - | - | - |
|  | Non-GMD controls | 36 | 114 | 20.36±2.09 | 20.53±2.28 | - | - | - | - |
| Lin et al. [29] | Obesity patients | 249 | 532 | 26.9±3.7 | 27.1±3.68 | 88.3±10.1 | 88.9±11.1 | 0.900±0.061 | 0.897±0.056 |
| Jin et al. [30] | PNS patients | 18 | 90 | 16.98±0.54 | 17.06±0.43 |  |  | - | - |
| Sun et al. [31] | General subjects | 88 | 124 | 17.6±3.2 | 20.0±3.5 | - | - | 0.9±0.1 | 0.9±0.1 |
| Sun et al. [32] | MetS patients | 12 | 30 | 28.75±2.61 | 27.55±1.73 | 96.67±5.73 | 96.50±4.6 | - | - |
| Rojek et al. [33] | Hypertensive patients | 86 | 119 | 29.7±4.9 | 28.22±4.27 | - | - | - | - |
| Albuquerque et al. [34] | General subjects | 291 | 412 | 19.4±3.4 | 19.84±3.44 | 66.6 ± 7.5 | 67.8±7.86 | - | - |
| Susanne et al. [35] | Overweight/obesity patients | 8 | 16 | 27.2±1.9 | 27.75±3.09 | 101.4±4.7 | 101.3±8.3 | - | - |
| Saremi et al. [36] | CAD patients | 53 | 92 | 29.4±4.7 | 29.2±4.4 | - | - | - | - |
|  | General/control subjects | 81 | 64 | 24.6±3.5 | 24.58±3.4 | - | - | - | - |
| Shokouhi et al. [37] | T2DM patients | 127 | 46 | 28.91±5.04 | 28.52±4.41 | - | - | 0.94±0.065 | 0.93±0.07 |
|  | Non-T2DM controls | 159 | 14 | 26.93±4.38 | 26.71±3.99 | - | - | 0.91±0.06 | 0.91±0.08 |
| Nishida et al. [38] | General subjects | 32 | 80 | 25.9±2.7 | 25.55±2.24 | 90.4±5.9 | 90.18±5.72 | - | - |
| Steinbacher et al. [39] | General subjects | 13 | 15 | 28.2±5.05 | 27.4±12.01 | 101.7±12.67 | 100.4±7.75 | - | - |
| Vazquez-Del Mercado et al. [40] | Overweight/obesity patients | 112 | 110 | 29.73±3.87 | 29.79±4 | 97.08±11.7 | 96.04±10.98 | 0.91±0.13 | 0.89±0.12 |
|  | Non- overweight/obesity controls | 77 | 76 | 22.49±1.7 | 22.41±1.6 | 78.1±8.1 | 78.52±8.29 | 0.82±0.08 | 0.81±0.13 |
| Ha et al. [41] | General subjects | 97 | 189 | 17.8±3.1 | 18.15±3.05 | 63.8±9.0 | 64.15±8.72 | - | - |
| Tai et al. [43] | Obesity patients | 59 | 118 | 40.6±7.9 | 42.53±7.27 | - | - | - | - |
| Tobina et al. [45] | General subjects | 34 | 85 | 24.1±2.5 | 23.7±3.48 | - | - | - | - |
| Ramos-Lopez et al. [46] | Obesity patients | 50 | 57 | 31.9±3.5 | 31.6±3.8 | 104.3±10.4 | 102.8±10.5 | - | - |
| Reddy et al. [47] | PCOS subjects | 38 | 80 | 22.68±6.60 | 22.97±5.54 | - | - | - | - |
| Zehsaz et al. [48] | General subjects | 206 | 376 | 19.09±4.22 | 18.74±3.71 | - | - | - | - |
| Zhang et al. [49] | NAFLD patients | 107 | 7 | 26.49±2.66 | 27.73±3.47 | - | - | - | - |
| Bailen et al. [50] | General subjects | 70 | 6 | 23.94±3.53 | 25.40±1.42 | - | - | - | - |
| Oguz et al. [51] | T2DM patients | 25 | 75 | 28.66±3.95 | 31.84±6.23 | - | - | - | - |

PGC1α, peroxisome proliferator-activated receptor alpha; BMI, body mass index; WC, waist circumference; WHR, waist-to-hip ratio; T2DM, type 2 diabetes mellitus; PCOS, polycystic ovary syndrome; NAFLD, nonalcoholic fatty liver disease; CAD, coronary artery disease; IGR, impaired glucose regulation; GMD, gestational diabetes mellitus; PNS, primary nephrotic syndrome; MetS, metabolic syndrome.

**Table S8.** Original data of the indexes of insulin resistance by genotypes of the PGC1α rs8192678 polymorphism.

| **Authors, reference** | **Subjects** | **N** | | **GLU, mg/dL** | | **INS, μU/mL** | | **HOMA-IR** | |
| --- | --- | --- | --- | --- | --- | --- | --- | --- | --- |
|  |  | GG | AA+AG | GG | AA+AG | GG | AA+AG | GG | AA+AG |
| Ek et al. [1] | General subjects | 243 | 248 | 92.88±8.36 | 91.8±8.95 | 5.78±2.88 | 5.61±3.1 | - | - |
| Hara et al. [2] | Non-diabetic subjects | 178 | 359 | 93.78±9.61 | 93.27±10.81 | 5.41±2.74 | 5.95±2.75 | 1.48±0.8 | 1.62±0.81 |
| Stumvoll et al. [3] | General subjects | 267 | 359 | 88.84±9.58 | 87.54±10.42 | 7.38±5.3 | 6.93±4.38 | - | - |
| Wang et al. [4] | T2DM patients | 6 | 14 | 201.47±59.07 | 229.45±82.49 | - | - | - | - |
| Ambye et al. [5] | General subjects | 931 | 1324 | 86.4±9 | 86.01±9.03 | 5.33±4.38 | 5.05±3.94 | 1.4±1.2 | 1.3±1.12 |
| Vohl et al. [6] | T2DM/obesity patients | 37 | 51 | 132.48±42.66 | 154.2±54.01 | - | - | - | - |
|  | Obesity patients | 70 | 112 | 95.94±12.42 | 100.31±15.11 | - | - | - | - |
| Wang et al. [7] | General subjects | 41 | 70 | 85.68±11.34 | 89.28±9.18 | 3.81±2.70 | 6.9±3.40 | - | - |
| Shan et al. [10] | T2DM patients | 52 | 128 | 149.3±47.75 | 160.32±79.01 | 2.53±0.63 | 2.32±0.47 | - | - |
|  | Non-T2DM controls | 36 | 86 | 81.72±27 | 84.01±16.19 | - | - | - | - |
| Zhang et al. [11] | T2DM patients | 97 | 166 | 125.82±30.06 | 133.02±48.24 | - | - | - | - |
|  | Non-T2DM controls | 144 | 138 | 82.08±11.34 | 87.66±12.24 | - | - | - | - |
| Song et al. [12] | T2DM/overweight/obesity patients | 29 | 70 | - | - | 13.88±9.42 | 15.71±10.29 | 4.80±3.55 | 5.21±3.85 |
|  | Overweight/obesity patients | 24 | 68 | - | - | 13.22±8.92 | 14.97±9.07 | 2.87±2.01 | 3.19±1.9 |
|  | Non-T2DM/overweight/obesity controls | 33 | 70 | - | - | 6.68±4.83 | 6.68±5.35 | 1.34±0.92 | 1.41±1.09 |
| Hui et al. [14] | NAFLD patients | 41 | 55 | 93.6±18 | 100.8±21.6 | 8.5±4.8 | 9.7±5.3 | 1.9±1.2 | 2.4±1.4 |
|  | Non-NAFLD controls | 33 | 63 | 86.4±12.6 | 90±10.8 | 5.9±2.5 | 5.8±2.6 | 1.3±0.6 | 1.2±0.3 |
| Okauchi et al. [15] | Male T2DM patients | 21 | 53 | 165.3±45.3 | 171.24±50.81 | 8.5±6.5 | 7.42±5.51 | - | - |
|  | Female T2DM patients | 21 | 60 | 150.4±45.7 | 165.17±54.88 | 10.7±7.4 | 9.32±7.34 | - | - |
| Goyenechea et al. [16] | Obesity patients | 73 | 107 | 91.98±7.74 | 90.4±6.99 | 9±5.6 | 9.27±7.13 | 1.9±1.4 | 2.04±1.59 |
| Weng et al. [20] | Obesity patients | 155 | 312 | - | - | 16.3 ± 9.8 | 16.32±9.03 | 3.91±2.92 | 3.88±2.5 |
|  | Non-obesity controls | 185 | 397 | - | - | 12.1±6.6 | 11.19±5.81 | 2.81±1.7 | 2.57±1.47 |
| Chae et al. [21] | PCOS patients | 46 | 138 | 92.7±27.9 | 86.88±8.88 | 14.8±8.9 | 12.48±7.74 | 3.6±2.9 | 2.67±1.84 |
|  | Non-PCOS controls | 72 | 184 | 89.0±7.6 | 89.21±8.45 | 9.5±7.2 | 7.41±3.67 | 2.1±1.6 | 1.6±0.75 |
| Zhang et al. [22] | T2DM patients | 70 | 171 | 160.56±66.24 | 160.51±60.75 | 8.36±4.34 | 10.94±13.74 | 3.13±1.55 | 4.33±5.14 |
| Geloneze et al. [25] | Obesity patients | 26 | 29 | 87±11 | 98±27 | 15.5±11.6 | 17.8±11.3 | 4.99±4.11 | 5.21±4.10 |
| Mirzaei et al. [26] | Obesity patients/controls | 106 | 123 | 97.29±14.48 | 106.82±43.11 | 10.64±4.742 | 15.89±13.45 | 2.62±1.39 | 4.49±4.81 |
| Lin et al. [29] | Obesity patients | 249 | 532 | 85.9±8.5 | 86.7±11.9 | 15.4±13.4 | 18.4±23.1 | 3.27±2.79 | 3.97±5.44 |
| Jin et al. [30] | PNS patients | 18 | 90 | 87.3±2.16 | 87.81±2.31 | - | - | 0.76±0.17 | 0.75±0.16 |
| Sun et al. [31] | General subjects | 88 | 124 | 93.6±23.4 | 104.4±37.8 | - | - | - | - |
| Sun et al. [32] | MetS patients | 12 | 30 | 92.16±13.14 | 100.26±12.24 | 15.64±8.58 | 12.17±5.45 | 3.66±2.38 | 3.07±1.54 |
| Rojek et al. [33] | Hypertensive patients | 86 | 119 | 97.5±9.1 | 94.75±10.25 | - | - | - | - |
| Susanne et al. [35] | Overweight/obesity patients | 8 | 16 | 97.5±10.3 | 95.45±8.59 | - | - | - | - |
| Saremi et al. [36] | CAD patients | 53 | 92 | 90.2±2.2 | 82.1±2.3 | - | - | - | - |
|  | General/control subjects | 81 | 64 | 89.6±9.1 | 87.4±9 | - | - | - | - |
| Shokouhi et al. [37] | T2DM patients | 127 | 46 | 165.24±50.04 | 165.24±48.78 | 9.78±3.48 | 10.22±3.22 | 3.92±1.78 | 4.14±1.74 |
|  | Non-T2DM controls | 159 | 14 | 94.86±10.62 | 97.56±7.2 | 7.38±2.03 | 7.02±2.4 | 1.72±0.48 | 1.7±0.61 |
| Nishida et al. [38] | General subjects | 32 | 80 | 100.3±13.3 | 99.87±9.35 | - | - | - | - |
| Vazquez-Del Mercado et al. [40] | Overweight/obesity patients | 112 | 110 | 97.34±13.17 | 94.88±11.82 | 17.77±15.25 | 21.65±25.91 | 4.43±3.99 | 4.92±5.93 |
|  | Non-overweight/  obesity controls | 77 | 76 | 87 ± 9.3 | 87.11±12.83 | 11.3±12.9 | 16.82±31.81 | 2.40±2.89 | 3.52±6.48 |
| Ha et al. [41] | General subjects | 97 | 189 | 90.1±5.8 | 88.54±10.04 | 6.9 ± 3.1 | 7.57±4.4 | 1.54±0.73 | 1.69±1.03 |
| Queiroz et al. [42] | General subjects | 38 | 109 | 83.27±8.06 | 81.33±8.76 | - | - | - | - |
| Tai et al. [43] | Obesity patients | 59 | 118 | 108.3±34.9 | 104.07±29.28 | 24.7±20.6 | 27.06±22.79 | 6.3±5.3 | 6.77±5.44 |
| Csep et al. [44] | MetS patients | 134 | 162 | 124.01±42.49 | 121.57±42.71 | - | - | - | - |
|  | Non-MetS controls | 90 | 76 | 93.19±10.94 | 98.12±14.25 | - | - | - | - |
| Tobina et al. [45] | General subjects | 34 | 85 | 104±14 | 102.32±19.25 | 7.5±4.5 | 7.74±7.62 | 1.9±1.1 | 1.9±1.96 |
| Ramos-Lopez et al. [46] | Obesity patients | 50 | 57 | 96.4±8.7 | 94.8±13.6 | - | - | - | - |
| Oguz et al. [51] | T2DM patients | 25 | 75 | 158.4±54.54 | 189.36±58.64 | - | - | - | - |

PGC1α, peroxisome proliferator-activated receptor gamma coactivator-1alpha; GLU, glucose; INS, insulin; HOMA-IR, homeostasis model assessment of insulin resistance; T2DM, type 2 diabetes mellitus; NAFLD, nonalcoholic fatty liver disease; PCOS, polycystic ovary syndrome; PNS, primary nephrotic syndrome; MetS, metabolic syndrome.

**Table S9.** Original data of blood lipids by genotypes of the PGC1α rs8192678 polymorphism.

| **Authors, reference** | **Subjects** | **N** | | **TG, mg/dL** | | **TC, mg/dL** | | **LDL-C, mg/dL** | | **HDL-C, mg/dL** | |
| --- | --- | --- | --- | --- | --- | --- | --- | --- | --- | --- | --- |
|  |  | GG | AA+AG | GG | AA+AG | GG | AA+AG | GG | AA+AG | GG | AA+AG |
| Ek et al. [1] | General subjects | 243 | 248 | 111.57±62 | 114.21±69.38 | 229.73±40.13 | 229.38±44.76 | - | - | 56.47±15.55 | 56.9±17.22 |
| Wang et al. [4] | T2DM patients | 6 | 14 | 292.88±162.84 | 300.76±275.35 | 189.62±31.9 | 191.08±77.34 | 116.49±24.01 | 121.6±51.73 | 73.27±10.34 | 69.3±27.45 |
| Ambye et al. [5] | General subjects | 931 | 1324 | 123.96±88.55 | 123.96±86.67 | 239.7±42.54 | 235.89±42.52 | 158.55±38.67 | 154.68±38.66 | 54.14±15.47 | 54.98±15.55 |
| Vohl et al. [6] | T2DM/obesity patients | 37 | 51 | 207.2±126.62 | 200.77±85.4 | 189.1±38.28 | 180.38±36.39 | 107.12±30.16 | 97.57±35.25 | 44.86±14.31 | 43.31±11.32 |
|  | Obesity patients | 70 | 112 | 172.66±91.2 | 159.11±78.01 | 198.38±37.51 | 192.38±35.41 | 119.1±30.55 | 114.36±31.75 | 44.08±10.44 | 47.82±12.93 |
| Wang et al. [7] | General subjects | 41 | 70 | 99.17±55.78 | 146.1±78.81 | 176.34±23.59 | 189.1±36.35 | 99.77±20.88 | 115.24±27.46 | 60.71±13.92 | 52.98±10.83 |
| Lu et al. [9] | T2DM patients | 44 | 76 | 115.11±61.98 | 179.54±112.1 | 201.08±46.4 | 215.9±46.38 | 116.01±34.8 | 119.88±34.57 | 58.78±8.12 | 54.38±8.5 |
| Zhang et al. [11] | T2DM patients | 97 | 166 | 165.58±146.98 | 162.04±114.22 | 206.5±49.88 | 206.5±61.1 | 115.62±39.44 | 115.62±47.95 | 57.23±13.15 | 58.39±13.92 |
|  | Non-T2DM controls | 144 | 138 | 142.56±99.17 | 144.33±92.09 | 166.67±54.14 | 187.55±66.51 | 79.66±49.11 | 97.45±61.49 | 61.87±17.01 | 60.33±15.47 |
| Song et al. [12] | T2DM/overweight/obesity patients | 29 | 70 | 169.12±61.98 | 249.7±177.79 | 197.6±33.33 | 208.82±45.26 | 115.24±24.99 | 110.21±38.82 | 41.76±10.39 | 39.44±9.71 |
|  | Overweight/obesity patients | 24 | 68 | 147.87±91.07 | 171.78±131.44 | 172.85±36.01 | 185.23±47.83 | 104.8±34.1 | 114.08±35.05 | 45.24±7.59 | 45.63±9.57 |
|  | Non-T2DM/overweight/obesity controls | 33 | 70 | 98.28±71.23 | 89.43±51.87 | 174.02±44.41 | 174.02±42.08 | 97.06±33.32 | 101.32±32.38 | 54.91±17.75 | 53.75±12.97 |
| Hui et al. [14] | NAFLD patients | 41 | 55 | 283.34±115.11 | 327.62±168.24 | 224.29±146.95 | 216.55±42.54 | 139.21±30.94 | 139.21±19.34 | 77.34±38.67 | 77.34±30.94 |
|  | Non-NAFLD controls | 33 | 63 | 177.09±141.67 | 194.8±132.82 | 220.42±42.54 | 228.15±46.4 | 131.48±30.94 | 131.48±23.2 | 85.07±50.27 | 81.21±34.8 |
| Okauchi et al. [15] | Male T2DM patients | 21 | 53 | 157.5±99.1 | 152.71±81.84 | 192.7±41.1 | 201.37±45.4 | - | - | 51.5±17.9 | 46.53±12.03 |
|  | Female T2DM patients | 21 | 60 | 130.6±69.1 | 139.98±74.79 | 207.1±39 | 211.91±36.89 | - | - | 46.4±10.6 | 50.54±12.12 |
| Goyenechea et al. [16] | Obesity patients | 73 | 107 | 94.74±58.44 | 97.52±67.13 | 209.98±34.8 | 203.4±37.51 | 136.89±30.16 | 129.76±32.44 | 52.98±12.37 | 52.4±13.36 |
| Chen et al. [19] | Hypertension/T2DM patients | 134 | 293 | 173.55±92.26 | 165.4±91.56 | 203.4±40.28 | 210.96±40 | 125.68±35.77 | 131.96±34.01 | 44.47±8.91 | 44.57±11.03 |
| Chae et al. [21] | PCOS patients | 46 | 138 | 121.7±69.9 | 104.98±62.52 | 188.4±35.6 | 171.03±36.41 | - | - | 54.3±14.9 | 62.34±17.07 |
|  | Non-PCOS controls | 72 | 184 | 71.9±31.1 | 67.95±22.22 | 173.5±27.7 | 167.78±25.97 | - | - | 64.8±13 | 63.4±12.9 |
| Zhang et al. [22] | T2DM patients | 70 | 171 | 231.1±270.95 | 279.18±302.57 | 209.2±72.7 | 181.76±74.69 | 114.85±38.67 | 110±42.72 | 52.59±23.59 | 50.79±28.08 |
| Nikitin et al. [23] | CAD patients | 135 | 178 | 147.87±12.4 | 145.17±12.65 | 224.29±38.67 | 209.43±35.35 | 137.67±11.6 | 150.86±12.72 | 56.07±6.96 | 55.27±8.6 |
|  | Non-CAD controls | 54 | 78 | 128.39±7.97 | 128.05±8.08 | 177.88±30.94 | 195.73±36.34 | 103.64±7.73 | 114.06±8.58 | 63.03±6.19 | 65.98±7.15 |
| Geloneze et al. [25] | Obesity patients | 26 | 29 | 110±33 | 151±16 | 175±26 | 174±38 | 109±22 | 111±29 | 45±12 | 41±9 |
| Mirzaei et al. [26] | Obesity patients/controls | 106 | 123 | 122.03±56.71 | 121.43±54.9 | 181.67±29.79 | 162.04± 28.07 | 103.64±22.01 | 88.91±21.41 | 44.64±10.59 | 44.59±10.99 |
| Deng et al. [27] | IGR patients | 156 | 205 | 220.48±139.02 | 191.26±130.16 | 202.24±39.06 | 209.2±37.51 | 111.76±35.96 | 119.49±41.38 | 45.63±8.12 | 48.72±11.99 |
| Lin et al. [29] | Obesity patients | 249 | 532 | 94.3±44.5 | 97.9±51.0 | 156.7±32.0 | 157.8±35.4 | - | - | 47.1±10.8 | 46.3±11.3 |
| Jin et al. [30] | PNS patients | 18 | 90 | 245.27±30.99 | 256.45±41.14 | 402.94±34.42 | 378.16±17.54 | - | - | 54.91±4.25 | 58.18±4.67 |
| Sun et al. [31] | General subjects | 88 | 124 | 115.11±17.71 | 123.96±44.27 | 185.62±42.54 | 181.75±50.27 | 112.14±7.73 | 112.14±19.34 | 54.14±42.54 | 42.54±15.47 |
| Sun et al. [32] | MetS patients | 12 | 30 | 165.58±69.95 | 240.84±109.8 | - | - | - | - | 48.72±17.79 | 37.12±6.57 |
| Rojek et al. [33] | Hypertensive patients | 86 | 119 | 157±66 | 164.48±78.08 | 205±38 | 203.29±41.38 | 126±35 | 123.51±35.86 | 49±15 | 49.42±14.47 |
| Susanne et al. [35] | Overweight/obesity patients | 8 | 16 | 97.3±61.5 | 100.8±46.62 | - | - | - | - | 54.0±11.5 | 60.85±16.62 |
| Saremi et al. [36] | CAD patients | 53 | 92 | 110.1±2 | 104.2±2 | 42.2±1.8 | 48.5±1.8 | 88.1±32.2 | 89.9±35.5 | 52.4±11.9 | 49.7±11.9 |
|  | General/control subjects | 81 | 64 | 76.5±1.5 | 71.9±1.5 | 48±1.6 | 44.7±1.7 | 50.6±13.3 | 52.4±12 | 39.7±10.3 | 39.7±11.8 |
| Shokouhi et al. [37] | T2DM patients | 127 | 46 | 184.17±41.62 | 182.4±42.5 | 196.06±43.31 | 204.18±34.03 | 132.25±37.12 | 131.48±34.8 | 36.35±9.28 | 35.58±9.67 |
|  | Non-T2DM controls | 159 | 14 | 146.1±39.85 | 146.98±47.81 | 177.88±41.76 | 194.12±44.08 | 117.56±30.94 | 117.56±31.71 | 117.17±27.07 | 105.57±22.82 |
| Nishida et al. [38] | General subjects | 32 | 80 | 142.7±59.4 | 161.11±131.44 | 206.8±27.8 | 215.12±31.48 | 132.9±27.1 | 132.99±27.28 | 49.0±10.6 | 50.41±11.94 |
| Vazquez-Del Mercado et al. [40] | Overweight/obesity patients | 112 | 110 | 180.91±117.04 | 143.44±62.01 | 194.66±37.97 | 189.36±36.93 | 114.05±31.04 | 114.53±35.35 | 36.66±12.54 | 36.56±14.65 |
|  | Non-overweight/obesity controls | 77 | 76 | 117±70 | 113.21±58.54 | 172±32 | 172.58±41.95 | 106±29.4 | 105±41.44 | 39±17.6 | 41±16.24 |
| Ha et al. [41] | General subjects | 97 | 189 | 81.9±30.7 | 79.78±37.76 | 171.9±28.5 | 168.5±29.51 | - | - | 59.5±11.6 | 56.93±11.64 |
| Queiroz et al. [42] | General subjects | 61 | 47 | 65.7±28.3 | 95.0±72.5 | - | - | 84.2±28.7 | 85.7±24.3 | - | - |
| Tai et al. [43] | Obesity patients | 59 | 118 | 158.6±126.2 | 145.28±89.32 | 191.6±35.6 | 199.85±40.48 | 127.6±31.6 | 134.5±38.32 | 45.0±10.0 | 47.69±11.18 |
| Csep et al. [44] | MetS patients | 134 | 162 | 198.51±137.69 | 208.17±131.44 | 210.78±52.43 | 212.01±51.43 | - | - | - | - |
|  | Non-MetS controls | 90 | 76 | 107.61±73.85 | 96±36.3 | 190.71±38.97 | 193.97±33.02 | - | - | - | - |
| Tobina et al. [45] | General subjects | 34 | 85 | 119±88 | 103.78±46.81 | 212 ± 33 | 210.93± 29.68 | 130±31 | 129.27±27.66 | 58±12 | 61.64±15.03 |
| Ramos-Lopez et al. [46] | Obesity patients | 50 | 57 | 109.5±60.7 | 108.1±59.4 | 221.1±37.4 | 214.3±35.1 | 144.1±30.1 | 142.2±31.1 | 54.9±14.2 | 50.4±9.8 |
| Zhang et al. [49] | NAFLD patients | 107 | 7 | - | - | 215.39±31.71 | 208.82±26.3 | 128±23.59 | 123.74±19.72 | - | - |
|  | Non-NAFLD controls | 189 | 13 | - | - | - | - | - | - | 51.04±9.28 | 45.63±10.83 |
| Oguz et al. [51] | T2DM patients | 25 | 75 | 164.69±142.56 | 178.86±113.43 | 204.18±35.96 | 208.34±45.96 | 124.52±30.16 | 126.53±38.29 | 49.5±10.44 | 48.4±10.52 |

PGC1α, peroxisome proliferator-activated receptor gamma coactivator-1alpha; TG, triglycerides; TC, total cholesterol; LDL-C, low-density lipoprotein cholesterol; HDL-C, high-density lipoprotein cholesterol; T2DM, type 2 diabetes mellitus; NAFLD, nonalcoholic fatty liver disease; CAD, coronary artery disease; PCOS, polycystic ovary syndrome; MetS, metabolic syndrome; PNS, primary nephrotic syndrome; IGR, impaired glucose regulation.
